# Tailoring in eHealth lifestyle interventions targeting people with cardiometabolic conditions and lower socioeconomic position: A scoping review

**DOI:** 10.1371/journal.pdig.0001713

**Published:** 2026-09-11

**Authors:** Sandra Van Mellaert, Martha S. Kreuzberg, Iris ten Klooster, Bert-Jan F. van Beijnum, Jan N. van Rijn, Monique Tabak

**Affiliations:** 1 Department of Biomedical Signals and Systems, Faculty of Electrical Engineering, Mathematics, and Computer Science, University of Twente, Enschede, the Netherlands; 2 Department of Health, Psychology and Technology, Faculty of Behavioral Management & Social Sciences, University of Twente, Enschede, the Netherlands; 3 Leiden Institute of Advanced Computer Science, Leiden University, Leiden, the Netherlands; Maastricht University Cardiovascular Research Institute Maastricht: Universiteit Maastricht Cardiovascular Research Institute Maastricht, NETHERLANDS, KINGDOM OF THE

## Abstract

eHealth interventions can support healthy lifestyle change for preventing and managing cardiometabolic conditions. While most eHealth interventions are designed for the general population, these conditions are more prevalent in people from lower socioeconomic backgrounds. Tailoring interventions to the needs and characteristics of this population can increase adherence and engagement, thereby enhancing the overall intervention effectiveness. However, evidence on how to tailor eHealth interventions to users from low socioeconomic backgrounds remains limited. Therefore, this scoping review examines the tailoring approaches implemented within eHealth lifestyle interventions targeting people with cardiometabolic conditions and low socioeconomic position (SEP). We focus on what is being tailored, the tailoring variables, the algorithms used for tailoring, and how the tailoring approaches are evaluated. Using keywords related to low SEP, cardiometabolic conditions, eHealth interventions, lifestyle, and tailoring, we searched electronic databases including Scopus, Web of Science, PubMed, and PsycINFO. We identified 43 eligible articles, with 27 unique eHealth lifestyle interventions targeting primarily diet and exercise. The literature shows a variety of tailoring approaches, albeit with a trend towards tailoring to socioeconomic factors at the design stage. Most interventions (n = 26/27, 96%) used rule-based algorithms for tailoring, primarily through functions such as feedback selection (n = 17/27, 63%) or variable substitution (n = 16/27, 59%). Although evaluation of tailoring was missing from most studies (n = 15/27, 55%), the importance of sociocultural relevance, appropriate language, and health literacy-sensitive design was highlighted. These findings suggest that while tailoring is present in many interventions, the current approaches remain limited in the facilitating technology and dynamic adaptations to SEP-specific needs. Thus, future research should investigate the application of more advanced, but reproducible tailoring algorithms and rigorously evaluate the impact of different tailoring methods on intervention effectiveness. To synthesize our findings, we assembled a framework that encapsulates the key concepts from our review, in combination with envisioned future work.

## Author summary

eHealth interventions are increasingly used for lifestyle coaching to prevent or manage cardiometabolic diseases, such as diabetes. Tailoring these interventions can increase intervention adherence and eventually contribute to improved health outcomes for the users. People from low socioeconomic backgrounds are more vulnerable to cardiometabolic conditions and could especially benefit from such interventions. However, guidelines on how to tailor the lifestyle interventions to this group are lacking. Therefore, in this scoping review, we examined the tailoring approaches used within eHealth lifestyle interventions for this target group. We found that tailoring to socioeconomic factors mostly occurred at the design stage, using variables such as low literacy levels. The reviewed studies especially highlighted the importance of tailoring to the users’ sociocultural context, language and differing health literacy needs. We identified several gaps within the literature, including the limited use of non-rule-based algorithms and a general lack of evaluation of tailoring methods. Future research should further investigate the use of adaptive algorithms for tailoring and prioritize evaluation trial designs that can differentiate the impact of different tailoring methods. Based on our findings, we constructed a framework of key concepts identified in this review, together with envisioned future work, to guide tailoring in future interventions.

## Introduction

Chronic diseases have a globally high mortality rate amounting to 75% in 2021, with cardiometabolic conditions, such as cardiovascular diseases and diabetes, among the leading contributors [1]. The burden of cardiometabolic conditions is inherently tied to a healthy lifestyle [1,2], which is a crucial component in their prevention, management, and halting of exacerbations [3,4]. However, maintaining a healthy lifestyle is particularly challenging for people with lower socioeconomic position (SEP) – here defined as having low income, limited education and insecure employment [5]. Lower SEP is associated with poorer health literacy [6,7], which is a key predictor of health behaviors, influencing how people are able to obtain and use health-related knowledge, and consequently perceive and apply healthy living [6,8]. Additionally, social, economic and environmental barriers perpetuate a higher consumption of sugary and highly caloric foods over fruits and vegetables [9,10], and low levels of sports participation and adherence to physical activity guidelines [6,11,12]. These factors collectively increase vulnerability of people with lower SEP to cardiometabolic conditions, such as elevated blood glucose or obesity [1], metabolic syndrome [13], cardiovascular diseases [14,15], and diabetes [16].

Interventions for supporting healthy lifestyles and cardiometabolic health can be provided in a face-to-face setting [17,18] or through digital health (eHealth) technology [19,20]. In-person sessions, such as group exercise programs or counseling provided by (community) health workers can be time-demanding, laborious, costly for all involved parties [21], and not always effective [22]. In contrast, eHealth interventions are a cost-effective alternative [23] and can positively impact chronic disease management [19], resulting in symptom improvement, fewer hospitalizations, and better overall well-being, at reduced healthcare costs [24]. For example, they can deliver periodic reminders to users in everyday life, and thereby increase the saliency of the desired behavior [25].

However, most eHealth interventions are designed to target the general population and overlook the role that social determinants, such as socioeconomic position, area of residence, and healthcare access, play in the adherence to a healthy lifestyle [12,26,27]. Simply targeting lower SEP individuals with lifestyle interventions designed for high SEP populations is insufficient and leads to reduced effectiveness of the intervention [18,28]. eHealth interventions have a greater impact when they use tailoring, which can be defined as the adaptation of the intervention (elements), such as the timing of feedback messages, type of content, or its form, based on user-specific information, in order to increase the relevance of the intervention to the recipient [29].

Appropriately tailored intervention elements that address the cognitive, behavioral, and contextual circumstances of the user increase the likelihood of receptivity towards the desired behavior performance, and thus, adherence to the intervention [25,30]. This means that, specifically for people with cardiometabolic conditions and lower SEP, the social and environmental factors must be considered within lifestyle interventions to create meaningful support for behavior change. Otherwise, interventions miss the opportunity to improve usability [30,31], adherence [30], and health outcomes [32,33]. However, synthetized knowledge and guidelines for tailoring eHealth interventions to low-SEP populations are still lacking [34].

Therefore, in this scoping review, we aimed to investigate the existing tailoring approaches within eHealth lifestyle interventions targeting people with lower SEP and cardiometabolic conditions. More specifically, we will answer the following research questions (RQ):

RQ1: What are the tailoring approaches in terms of what is being tailored, at what level (in terms of granularity and dynamic adaptivity), and according to what tailoring variables (e.g., health condition, contextual information, low SEP)?
RQ2: What algorithms are used for executing the tailoring?
RQ3: How are tailoring approaches evaluated and at what phase of evaluation?

## Materials and methods

We chose to conduct a scoping review to identify research gaps [35,36] and to examine the existing evidence without requirements on quality assessment and restrictions of the included study designs [36]. We followed the framework of scoping review methodology defined by Arksey and O’Malley [36] and the Preferred Reporting Items for Systematic reviews and Meta-Analyses extension for Scoping Reviews (PRISMA-ScR) Checklist [37] (see **S1 Table**).

### Search strategy

To identify eligible studies, we formulated a search string using the Population, Context, Concept (PCC) framework and key concepts related to lower SEP, cardiometabolic conditions, eHealth interventions, lifestyle (regarding physical activity, nutrition, sleep and/or stress), and tailoring. For each concept, we selected the relevant keywords and their synonyms with the help of domain experts, an information specialist, existing literature, and the PubReMiner [38] tool. The search string (see **S1 Text**) was used to search the electronic databases of PubMed, Scopus, Web of Science, and PsycINFO on May 3^rd^, 2024, and for an update search on May 20^th^, 2025.

### Eligibility criteria

We structured the eligibility criteria (see Box 1) using relevant components of the PICO (Population, Intervention, Comparison, Outcome) framework [39]. Each PICO component was defined according to our review’s aims (e.g., requiring an intervention description), as well as the key concepts of the search string. Due to the exploratory nature of our review, we did not apply the framework’s criteria regarding comparisons or outcomes. Original peer-reviewed research or protocol papers, published in English, in or after the year 2000, and fulfilling the defined criteria were considered eligible.

**Box 1. Summary of eligibility criteria.**

**Type of publication**

- Peer-reviewed and accepted journal article, study protocol, conference paper, or a case study (i.e., original research), which can also be early access or a preprint, published in English, since 2000

**Type of study**

- Includes a description of the developed lifestyle eHealth intervention, or the study is an evaluation trial that connects to a protocol or another study that describes the intervention

**Type of intervention**

- eHealth intervention aiming for lifestyle modification in the areas of physical activity, nutrition, sleep, or stress (one or more)
- Implements a form of tailoring
- *Exclude*
  - Tailoring revolves around culture only
  - Primary focus on alcohol consumption or smoking, or on anxiety/depression/trauma treatment

**Population**

- The participant sample, or the design of the intervention includes adults (>= 18 years) with
  - Low SEP, described with regard to education level, income level, employment, place of residence, and/or low literacy (skills)
  - Cardiometabolic condition(s), or a clinical risk thereof, e.g., metabolic syndrome, diabetes, cardiovascular disease, pre-hypertension, hypertension, overweight/obesity

*Exclude*

- Low SEP is defined solely around race/ethnicity
- The participant sample includes individuals with psychological/neurological/ traumatic/mental disorders/disabilities, pregnant women, or women with gestational diabetes

### Study selection

Articles identified by the search string were exported into Covidence [40] for (automatic) de-duplication and to conduct the screening process. After de-duplication, the first author (SVM) screened the titles and abstracts of all the remaining articles, using the defined eligibility criteria. The second reviewer (MK) screened 20% of these articles (κ = 0.9), to ensure consistency. The reviewers met at the mid-point and at the end of the screening process to resolve any conflicts, following the procedure of Mark and Thomas [41] and Levac et al. [42]. Any additional doubts were consulted with the other authors until consensus. During the screening process, we amended the exclusion criteria once, to include articles that consider, but do not primarily focus on smoking or alcohol behaviors, which were initially excluded. SVM subsequently screened all full texts, with MK independently screening 20% (κ = 0.9) for verification. SVM checked the reference lists of the remaining papers for any additional eligible articles (backwards snowballing). Additionally, if the included articles contained a registration number, the corresponding register was searched for supplementary records that might have been missed during extraction. Records that described the same intervention (e.g., a trial and a protocol or a design paper) were merged in Covidence as one intervention.

### Data extraction

The data extraction form was partially based on the Cochrane review template and the TIDieR checklist [43] and aligned with our defined research questions, as outlined in **Table 1**. The extraction process was thoroughly discussed with the other co-authors and piloted by SVM and MK, to ensure clarity and consistency, and then conducted by SVM. For RQ1, we extracted details of the tailored components of each intervention and whether any tailoring was SEP-specific, the granularity of the tailoring (individual- or group-based), and how dynamically tailoring occurs. We also extracted the tailoring variables, i.e., the information collected about a user that is used as input to tailor the eHealth lifestyle intervention [44]. Additionally, we examined how the variables were collected and whether any were specific to SEP-related factors. For RQ2, we extracted details on the algorithms executing the tailoring. Lastly, for RQ3, we extracted the type of each study, the target and the included population, how the tailoring component was evaluated, and reported engagement with the (tailored) intervention components. We also extracted the evaluation phase of each study, following the framework of Jansen-Kosterink et al. [45], which aligns eHealth evaluations to technology readiness levels (TRL). Within merged studies where multiple records described the same intervention, we extracted the data simultaneously.

**Table 1:** Data extraction items per research question.

Topic	Extracted items
**General information**	• Title, Author, Publication year • Country in which the study was conducted • Lifestyle area(s) of focus (Diet, PA, Sleep, Stress) • Targeted low-SEP characteristics • Targeted health condition(s) • Name of the intervention / project • Role of technology in the intervention (digital only, blended care) • Mode of delivery
**Tailoring approaches (RQ1)**	• Tailoring areas: What is being tailored in the intervention (e.g., content, timing, representation) • Tailoring approaches and/or variables that explicitly address low-SEP factors of the target users Tailoring variables • General tailoring variables (unrelated to SEP factors) • Tailoring variables related to SEP factors • Source type of the tailoring variables (e.g., self-reported) • Methods used for obtaining the variables (e.g., questionnaires) Levels of tailoring • Granularity of tailoring • How dynamically the tailoring occurs
**Algorithms for tailoring (RQ2)**	• The function of the algorithm used for executing the tailoring • Type of algorithm (e.g., rule-based)
**Evaluation (RQ3)**	• Type of study • Phase of evaluation (Research, Development, Deployment*) or a protocol • The intended and included population, in terms of low-SEP characteristics and cardiometabolic conditions • Methods of evaluating the tailoring • Reported engagement with intervention (components)

PA, Physical activity; RQ, Research question

*According to Jansen-Kosterink et al. [45]

### Data synthesis

We used inductive coding to thematically cluster emerging themes in the extracted data regarding the tailoring variables and the algorithms used for the execution of tailoring. To assign the phase of evaluation, we used deductive coding to categorize each study with an evaluation component into one of the three: research, development or deployment [45]. This categorization was independently verified by the second reviewer (MK).

Lastly, we used a combination of inductive and deductive coding to analyze both the tailoring areas and the levels of tailoring. We thematically clustered the tailoring areas according to communication properties (content, timing, and representation [46]), but we also allowed for additional clusters that arose from the data. To analyze the levels of tailoring, we focused on two main concepts: the granularity of tailoring (i.e., how specific to an individual the intervention is) and dynamic adaptivity (i.e., how dynamically tailoring occurs). For the concept of granularity, we adopted the labels of group- and individual-based tailoring [29]. Using these labels, we categorized both the tailoring variables (input) and the output of each tailoring approach, as shown in **Table 2**. For dynamic adaptivity, we categorized the data according to whether tailoring occurs dynamically (tailoring variables are assessed each time before tailoring is activated) or statically (tailoring remains static after a baseline assessment, which can be repeated after a period of time) [47]. Furthermore, we inductively coded the extracted data into thematic clusters on how frequently the tailoring variables are collected (tailoring variable collection rate) and how frequently tailoring occurs in response to the collected variables (rate of tailoring). Any questions or uncertainties that emerged during the coding process were discussed with the other authors until consensus was reached.

**Table 2:** Definitions of labels for the granularity of the input (tailoring variables) used for tailoring and the tailored output that is provided to the recipient.

	Type of Granularity		Examples
*Input (tailoring variables)*	**Specific to an individual**	The input data is specific to an individual.	Body weight, step count, name
	**Part of a group**	The input data defines a characteristic relevant to a group of users.	Stage of behavioral change, gender
*Tailored output*	**Specific to an individual**	The output contains information that is specific to the individual, and it is unlikely other users will receive the same output.	Messages contain information specific to the individual, e.g.,: “Hello Bob, today you reached your goal of 5500 steps. Keep up the good work, ask Alice to join you for a walk tomorrow”.
	**Relevant to a group**	The output is specific but the same for all members of an identified group.	Messages are of a group-based nature, determined by, e.g., general rules, such as when a goal is reached, e.g.,: “Great job, you reached your physical activity goal for today”.

## Results

### Search results

**Fig 1:**
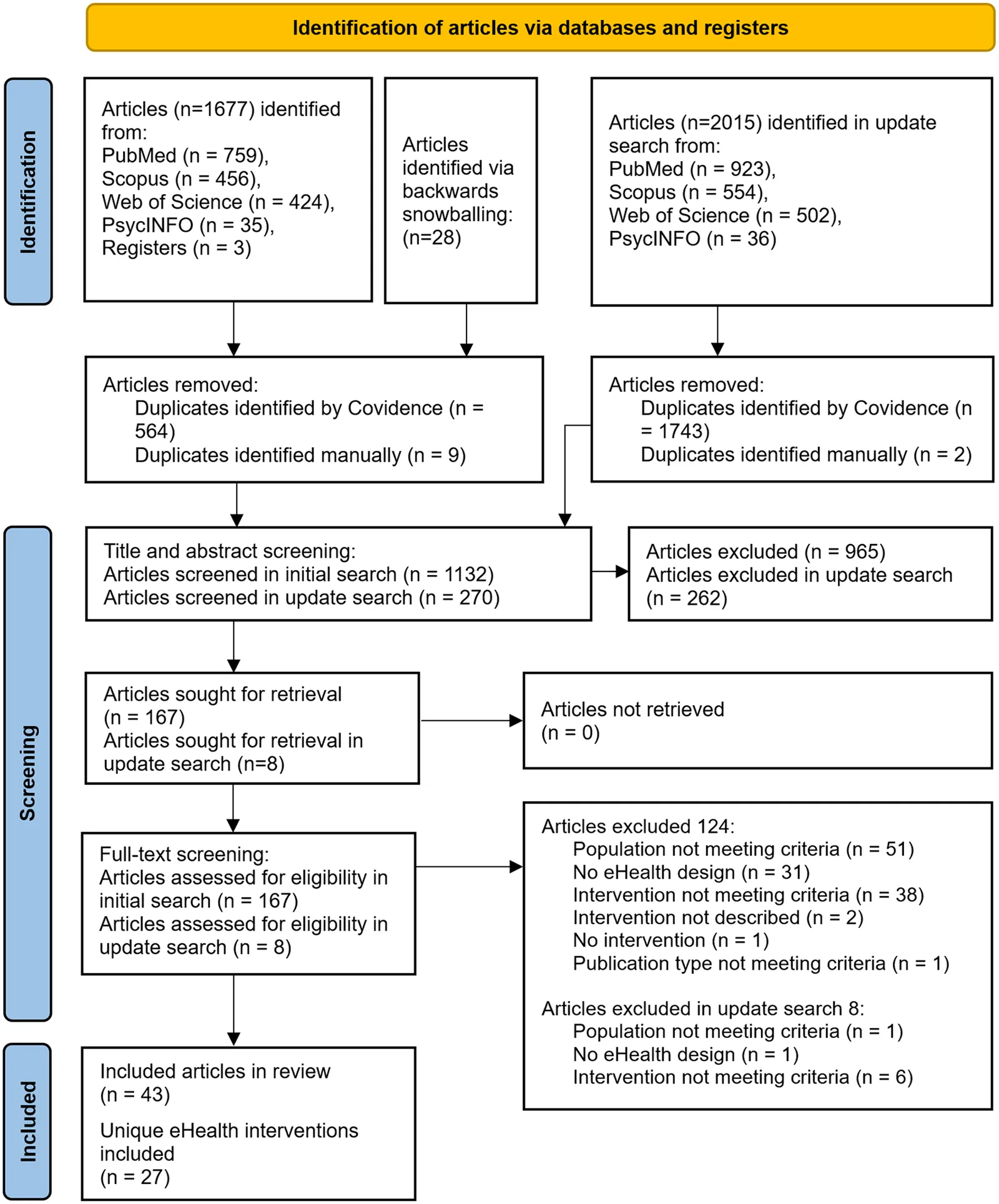
PRISMA flowchart outlining the article selection process. Adapted from: https://guides.lib.unc.edu/prisma.

We identified 1677 articles in the initial search, of which 564 articles were automatically marked as duplicates. Following the screening procedures (see Fig 1), 43 articles met eligibility criteria for inclusion in the review, describing 27 unique eHealth interventions (see **Table 3**). The update search did not identify additional eligible records.

**Table 3:** All included interventions, with the included papers and the intervention characteristics.

Intervention ID*	Papers included	Intervention name (if applicable)	Low-SEP descriptors of the (intended) target population	Targeted lifestyle area(s)	Mode of delivery (Blended care/ Digital only)	Evaluation phase**	Type of study
**eHealth interventions targeting overweight/obesity:**							
I-1	Dombrowski et al. 2020 [48]	Game of Stones	Area of residence, Education level, Ethnicity/race, Income, Low SEP, Other	PA, Diet	SMS (Digital only)	Development	Randomized feasibility trial
	Macaulay et al. 2022 [49]					–	RCT Protocol
I-2	Stein et al. 2019 [50]	Working for you	Income, Low SEP	PA, Diet	Automated phone calls (Blended care)	–	Protocol for a randomized trial
I-3	Kharmats et al. 2022 [51]	Monday-Focused Tailored Rapid Interactive Mobile Messaging intervention for Weight Management 2 (MTRIMM2)	Education level, Employment, Ethnicity/race	PA, Diet	Website (Digital only)	Development	RCT
I-4	Berger et al. 2019 [52]	In Balance	Income, Ethnicity/race, Medical vulnerability	PA, Diet, Sleep	SMS (Blended care)	–	RCT Protocol
I-5	Foley et al. 2012 [53]	the Shape Program	Income, Ethnicity/race, Medical vulnerability	PA, Diet	Mobile app (Blended care)	–	RCT Protocol
	Bennett et al. 2013 [54]					Development	RCT
I-6	Powell-Wiley et al. 2022 [55]	–	Area of residence, Ethnicity/race, Other	PA	Website (Digital only)	Research	Computer simulation
I-7	Foley et al. 2016 [56]	Track	Income, Employment, Area of residence, Ethnicity/race, Medical vulnerability	PA, Diet	SMS (Blended care)	–	RCT Protocol
	Bennett et al. 2018 [57]					Deployment	RCT
I-8	Walthouwer et al. 2013 [58]	Weight in the Balance	Education level	PA, Diet	Mobile app (Digital only)	–	RCT Protocol
	Walthouwer et al. 2015 [59]					Development	RCT
I-9	Clark et al. 2018 [60]	Addressing People and Place Microenvironments (APP-Me)	Income	PA, Diet	Web app; print + IVR (Blended care)	–	RCT Protocol
**eHealth interventions targeting T2DM:**							
I-10	Pathak et al. 2021 [61]	Diabetes and Mental Health Adaptive Notification Tracking and Evaluation (DIAMANTE) project	Income, Health literacy, Ethnicity/race	PA, Stress	SMS (Blended care)	Development	Crowdsourcing, feasibility, pilot, expert review
	Aguilera et al. 2020 [62]***					–	Clinical trial protocol
I-11	Schillinger et al. 2008 [63]	The Improving Diabetes Efforts Across Language and Literacy (IDEALL) project	(Health) Literacy, Medical vulnerability	PA, Diet, Stress	Mobile app (Blended care)	Development	Practical RCT
I-12	Wood et al. 2015 [64]	Diabetes 101	Income, Area of residence, Health literacy, Medical vulnerability, Other	PA, Diet, Stress	SMS; IVR (Digital only)	Development	Pilot
I-13	Alian et al. 2018 [65]	MobiDiaBT	Income, Education level, Area of residence, Health literacy, Ethnicity/race, Medical vulnerability, Other	PA, Diet	IVR (Digital only)	Research	Expert verification of simulated use-case scenarios
I-14	Ramirez et al. 2016 [66]	–	Income, Ethnicity/race	PA	SMS (Digital only)	Development	Qualitative study
	Ramirez & Wu 2017 [67]					Development	Randomized pilot
I-15	Gatwood et al. 2020 [68]	Management Of Diabetes in Everyday Life (MODEL) program	Area of residence, Ethnicity/race, Medical vulnerability	PA, Diet	SMS; IVR (Digital only)	Research	Qualitative
I-16	Mayberry et al. 2016 [69]	FAMS (Family-focused Add-on for Motivating Self-care)	Ethnicity/race, Low SEP	PA, Diet	Mobile app (Blended care)	Development	Feasibility study
	Nelson et al. 2018 [70]					–	RCT Protocol
I-17	Glasgow et al. 2011 [71]	Name of the website: My Path to Healthy Life” (“My Path,” for short) in English and “Mi Camino a la Vida Sana” (or “Mi Camino”) in Spanish	Health literacy, Digital literacy, Ethnicity/race	PA, Diet	Website (Blended care and digital only in different intervention arms)	Development	RCT
	Glasgow et al. 2012 [72]					Development	Pragmatic RCT
I-18	Azelton et al. 2021 [73]	Healthy at Home	Income, Medical vulnerability, Area of residence, Health literacy, Other	PA, Diet, Stress	Mobile app (Blended care)	Development	Pilot RCT
I-19	Karimi et al. 2020 [74]	EatSmart	Income, Education level, Ethnicity/race	Diet	Web app (Digital only)	–	Protocol
	Karimi et al. 2023 [75]					Development	Qualitative
I-20	Rowsell et al. 2015 [76]	Healthy Living with Diabetes (Diabetes Literacy project)	Health literacy, eHealth literacy	PA	SMS (Digital only)	Development	Qualitative study
	Muller et al. 2017 [77]					Development	International RCT
**eHealth interventions targeting (risk for) (pre-)hypertension:**							
I-21	Robles et al. 2022 [78]	myBPmyLife - myDataHelps app	Income, Ethnicity/race	PA, Diet	SMS; Website (Digital only)	Research	Qualitative
	Hellem et al. 2024 [79]					Research	Qualitative
	Golbus et al. 2024 [80]					–	RCT design and rationale
I-22	Hageman et al. 2014 [81]	Wellness for Women: DASHing towards Health	Area of residence, Medical vulnerability	PA, Diet	Mobile app (Blended care)	Development	RCT
**eHealth interventions targeting overweight/obesity, T2DM:**							
I-23	Ayre et al. 2019 [82]	Smart Snacking	Education level, Health literacy	Diet	SMS; IVR (Digital only)	–	RCT protocol
	Ayre et al. 2020 [83]					Development	Online RCT
**eHealth interventions targeting overweight/obesity, risk for CVD:**							
I-24	Tamura et al. 2020 [84]	Step It Up	Area of residence, Ethnicity/race, Income, Other	PA	SMS; Web app (Digital only)	–	RCT protocol
	Vijayakumar et al. 2022 [85]					Development	Pilot study
**eHealth interventions targeting overweight/obesity, hypertension:**							
I-25	Greaney et al. 2009 [86]	Be Fit, Be well (BFBW)	Income, Education level, Literacy, Numeracy, Medical vulnerability	PA, Diet	Web app (Blended care)	–	Design paper
	Bennett et al. 2012 [87]					Deployment	Randomized pragmatic trial
**eHealth interventions targeting CVD, risk for pre-hypertension:**							
I-26	Diez-Canseco et al. 2015 [88]	–	Income, Medical vulnerability, Other	PA, Diet	Web app, or print + IVR (Blended care)	Development	Feasibility & Acceptability study
	Rubinstein et al. 2016 [89]					Deployment	RCT
**eHealth interventions targeting pre-diabetes, T1DM, T2DM, (risk for) hypertension:**							
I-27	Schultz et al. 2022 [90]	GoalRx Prescription Intervention (GPI)	Income, Area of residence	PA	SMS (Blended care)	–	Protocol for an observational study

SEP, socioeconomic position; CVD, cardiovascular disease; T1DM, type 1 diabetes; T2DM, type 2 diabetes; PA, physical activity; SMS, Short Message Service; IVR, Interactive Voice Response; RCT, Randomized Controlled Trial.

* These IDs are used throughout the article to identify each intervention.

**According to Jansen-Kosterink et al. [45].

***Additional information on the reinforcement learning algorithm was taken from [91].

### General study information

**Fig 2:**
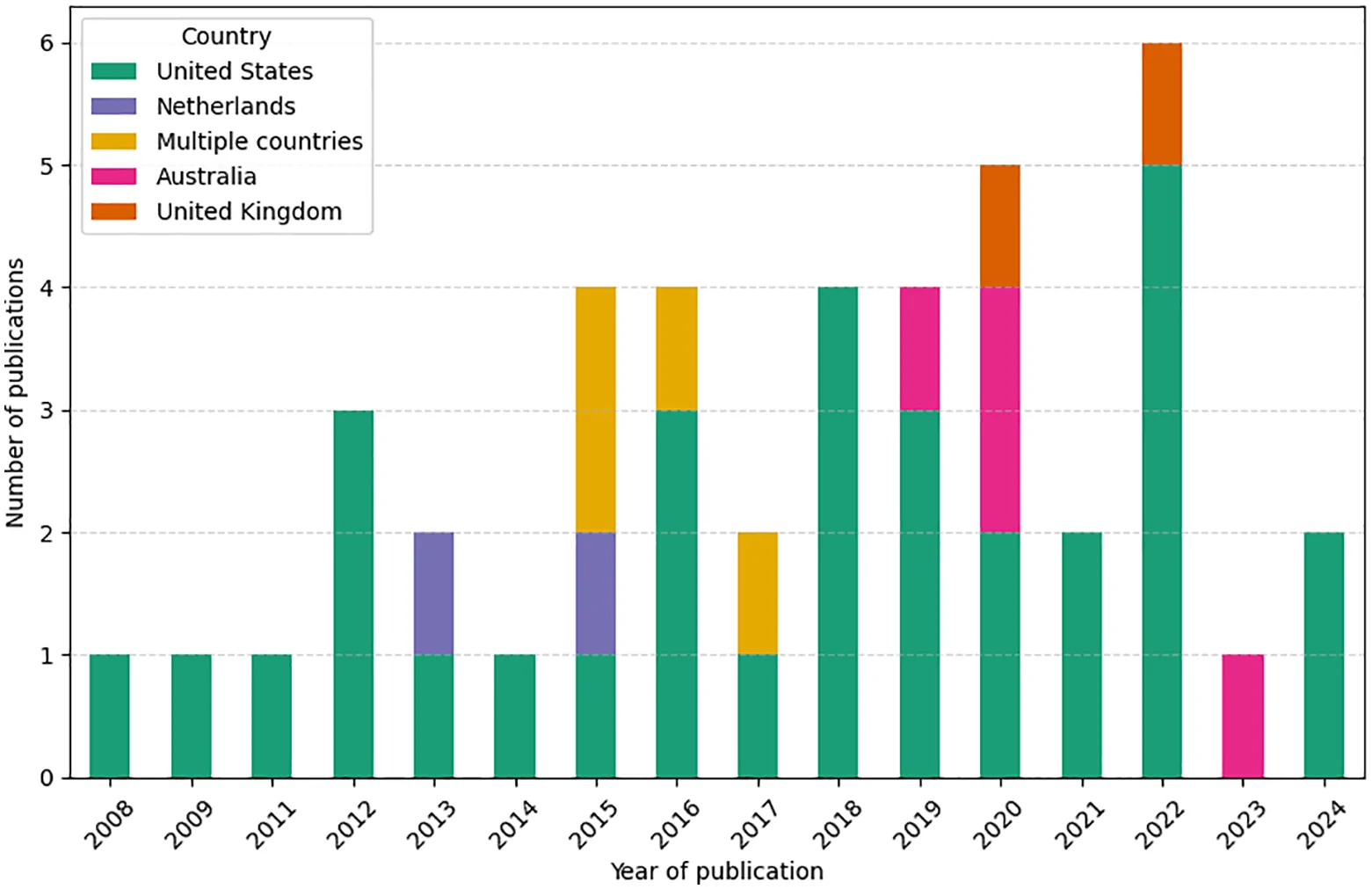
Distribution of publication years of all included records (n = 43). The color-coding identifies the countries where the studies were conducted.

The included papers were published between 2008 and 2024, most often originating from the United States (US) (n=31/43, 72%) (see Fig 2).

#### Targeted lifestyle areas.

Most of the eHealth interventions targeted two lifestyle areas, predominantly combining diet and physical activity (n = 15/27, 56%), while one (n = 1/27, 4%) combined physical activity with stress (I-10) (see **Table 3**). Five (n = 5/27, 18%) studies focused solely on physical activity (I-6, I-14, I-20, I-24, I-27), and two (n = 2/27, 7%) on diet (I-19, I-23). Some of the interventions combined three lifestyle areas, targeting diet and physical activity together with stress (n = 3/27, 11%) (I-11, I-12, I-18) or sleep (I-4) (n = 1/27, 4%).

#### Target population characteristics.

The US-based origin of most interventions was also reflected in the described (intended) target population characteristics related to low SEP. The majority used *Income* (n = 17/27, 63%) and *Ethnicity/race* (n = 15/27, 56%) (see **Table 3**). The third most common characteristic used (n = 11/27, 41%) was *Medical vulnerability*, often defined by insurance status, healthcare setting (e.g., low-resourced, medically underserved, or safety net center), or healthcare access. The *Other* category included determinants of social stability (I-6, I-18), or reduced available resources, such as limited opportunities for physical activity (I-24), or lack of transportation (I-12, I-13). The use of ethnicity/race and medical vulnerability may reflect the predominantly US-related contextual differences, such as race-based societal stratification or the lack of universal healthcare.

### Tailoring approaches

#### General tailoring areas.

The tailoring areas not specific to low-SEP factors fell into five main clusters: Content, Timing & frequency, Representation, Goal-setting, and Intervention settings.

**Content:** Most interventions (n = 26/27, 96%) tailored the content to the user. This could manifest in different forms, such as changing some aspects of an otherwise generic message (e.g., adding the user’s name (I-1, I-16, I-19, I-21)), selecting the most relevant *type* of content (e.g., type of feedback (I-2, I-3, I-4, I-5, I-7, I-8, I-11, I-14, I-17, I-18, I-20, I-25, I-27)), or generating content (e.g., a recommendation) to reflect the user’s current context (I-13). Additionally, the changes were often part of a feedback element, for example, to create a summary of goal progress (I-13, I-16) or insert the currently active goal description into a progress inquiry (I-4, I-16, I-27), so that appropriate feedback can be selected.

**Goal-setting:** Three (n = 3/27, 11%) interventions (I-2, I-14, I-24) implemented adaptations in a goal-setting feature where the algorithm either adaptively adjusted the user’s goal (I-24), or the users were provided with recommendations for increasing their current goals upon successful goal adherence (I-2, I-14). Additionally, five (n = 5/27, 19%) interventions incorporated prioritization (I-4, I-7) or recommendation of goals (I-8, I-20, I-27), based on, e.g., users’ preferences or health behaviors, typically at the start of the intervention.

**Timing & Frequency:** Numerous interventions (n = 12/27, 44%) tailored the timing of content delivery (e.g., of messages), typically with preferences (I-1, I-9, I-11, I-16, I-19, I-21), or triggers, such as the user’s location (I-24) as the input for tailoring. This was often (indirectly) tied to the frequency of receiving the intervention content, though sometimes the user could adjust the frequency directly by opting in to different frequency levels (I-1, I-9, I-15, I-16). In some cases, the timing was adapted jointly with content selection (I-10, I-24).

**Representation:** Several interventions (n = 7/27, 26%) tailored visualizations relating to goal progress or achievement (I-1, I-8, I-17, I-21, I-22, I-24, I-25), or medical data (e.g., blood pressure) (I-17, I-21, I-24). Other interventions (n = 3/27, 11%) allowed users to choose the modality for receiving the intervention (e.g., voice or text messages (I-14)), adapted images and videos according to the participant’s age, gender and culture (I-20), or used visual feedback representation (e.g., a stoplight representing blood glucose ranges (I-18)).

**Intervention settings:** Lastly, four interventions (n = 4/27, 15%) tailored various intervention settings, such as how some content is shown to the user (e.g., as optional or as compulsory based on an answer to a quiz question (I-12)), the proportion of messages that correspond with user preferences, which increased with low behavior adherence (I-15), or the level of provided support from outside the digital platform (from, e.g., health coaches) based on the user’s data (e.g., out of range values) (I-4, I-11).

#### General tailoring variables.

We identified nine clusters of variable types unrelated to low SEP that were used as input for tailoring, as shown in **Table 4**. The identified variables were largely user-reported, most frequently via an intake assessment. One intervention used Ecological Momentary Assessment (EMA) questionnaires that the users filled out for four weeks, prior to the start of the intervention, and during the intervention, the system acted according to the patterns found within the EMA data (I-9). Three interventions used connected scales that could automatically transfer data to the system (I-4, I-7, I-24); however, users still had to weigh themselves to produce the data.

**Table 4:** Tailoring variables (non-specific to low-SEP factors) used for tailoring the eHealth lifestyle interventions.

Variable type cluster	Included tailoring variables	Data collection	Source type
**Profiling** *Data collected before / at the start of the intervention that comprises the profile of the user*	**Demographic information** (I-1, I-8, I-10, I-15, I-16, I-21, I-19, I-20): name, age, gender, culture, language, income	Intake assessment	User-reported
	**Health profile** (I-10, I-13, I-15, I-21, I-27): medication, health needs and abilities, mobility	Intake assessment	User-reported (or system-derived)
	**Health behaviors** (I-4, I-7, I-8, I-10, I-13, I-15, I-17, I-20, I-27): (current) PA levels / patterns, dietary intake, other health behaviors, e.g., sleep, medication adherence	Intake assessment, interactive web features, online assessment	User-reported
**Health data** *Data collected during the intervention on the user’s health and goal/behavior status*	**Physiological (**I-1, I-2, I-3, I-4, I-7, I-8, I-10, I-13, I-17, I-18, I-21, I-22, I-24, I-25): Weight, BMI, blood pressure, (historical) steps, PA logs, baseline PA levels, low-sodium food choices (logs), glucose data (fasting blood glucose, HbA1c), cholesterol	Mobile/Web app, website, questionnaires via interactive SMS, IVR, in-person appointments, entered by third parties, e.g., health coaches	User-reported, or User-reported from a device (pedometer, blood pressure monitor)
	Steps, weight, blood pressure, blood glucose, sleep duration, heart rate	Fitbit, wireless (cellular) scale, blood pressure monitor, glucometer	Device-captured
	**Adherence** (I-2, I-4, I-5, I-7, I-8, I-11, I-14, I-15, I-16, I-21, I-27): (perceived) adherence/progress on goal, behavior, and/or action plans	Interactive SMS, IVR, web app	User-reported
		Mobile app	Device- captured
**User preferences** *Preferences indicated by the users upon the system’s inquiry / at the start / before the intervention* (I-1, I-6, I-9, I-11, I-13, I-14 I-15, I-16, I-17, I-19, I-21, I-24, I-27)	Preferences regarding timing of receiving messages, frequency of messages, frame of messages, modality, weight units PA preferences: PA type, location, resources, time of PA	Intake assessment	User-reported
**Contextual data** *Data describing the current context of the user* (I-3, I-6, I-9, I-10, I-13, I-21, I-24, I-27)	User’s location, weather, day of the week, day in the study, time of day, (currently) available local exercise facilities	Mobile app	Device-captured
	User’s location, grocery shopping day	Intake survey	User-reported
**System-derived data** *Data that the system can calculate/estimate based on other data (entered by, e.g., users, researchers, coaches)* (I-1, I-2, I-3, I-4, I-7, I-8, I-10, I-13, I-15, I-17, I-18, I-24, I-25)	Calculated goal/behavior progress, goal achievement, weight change, behavior prioritization, comparison to (national) PA guidelines	Online assessment, interactive SMS, IVR, web app, in-person appointments	User-reported
	Weight change, step average, missing tracking values, history of sent messages and their time frames	Wireless scale, Fitbit, intervention metadata	Device-captured
**Socio-behavioral** *Data relating to the social context of the user* (I-9, I-10, I-13)	Neighborhood scores, social connections and support, people that the users are frequently eating with	Intake survey, EMA questionnaires	User-reported
**Psychological** *Data relating to intrapersonal beliefs, psychological well-being, and/or determinants of behavior* (I-4, I-7, I-8, I-10, I-15, I-17, I-20, I-21, I-22, I-26)	Behavioral determinants (e.g., self-efficacy), stage of behavior and readiness for change, motivating factors, attitudes and barriers to reaching goals, weight-gain risk perception, perceived quality of life, loneliness, depression and anxiety scores	Intake survey or surveys repeated throughout the intervention	User-reported
**User action** *Data relating to actions of the user within interactive tools/features within the system* (I-3, I-4, I-9, I-12, I-13, I-20, I-23)	Feedback on messages, responses or requests in interactive tools	Like/dislike button on messages, quizzes, questionnaires, action planning or recommendation tools	User-reported
**Metadata** *Data that the system has access to because they have previously been entered (by users, researchers, coaches etc.) or stored by the system* (I-2, I-3, I-4, I-6, I-7, I-8, I-9, I-10, I-13, I-15, I-16, I-17, I-21, I-24, I-26, I-27)	Selected intervention options (e.g., goals, type of PA, targeted behavior, action plans), additional information (e.g., name of support person, PA coordinator number), geofence, health guidelines, learned patterns on frequent eating locations	Captured from the user’s answers to (intake) surveys / interactive features within the intervention, e.g., via interactive SMS, IVR, entered by third parties, e.g., researchers, health coaches, EMA questionnaires	User-reported
	Local PA centers metadata, restaurant information, historical data on sent messages and their timeframes	Database access	System-retrieved

BMI, body mass index; PA, physical activity; SMS, Short message service; IVR, Interactive voice response; EMA, Ecological Momentary Assessment.

#### General tailoring areas + variables.

With the nearly universal use of content tailoring, each variable type was employed in at least one intervention’s approach to tailor content, as shown in **Table 5**. Some variable types were noticeably more prevalent in certain tailoring areas; for example, to tailor timing & frequency, user preferences served as key input (n = 7/9, 78%), while representation tailoring (such as personalized graph visualizations) relied heavily on health data (n = 7/10, 70%). Notably, I-10 used a more computationally advanced approach for simultaneously tailoring content and timing, leveraging almost all variable types. These patterns highlight the versatility of content tailoring, as well as the dependency on the tailoring goal and technology, which may dictate the scope of included variables.

**Table 5:** The variables (not specific to low-SEP factors) each intervention used to tailor each of the tailoring areas.

Variables used	Content	Timing & Frequency	Content + Timing*	Representation	Goal-setting	Intervention settings	n**
**Profiling** - Demographics	I-1, I-8, I-15, I-16, I-19, I-21		I-10	I-20			3
**Profiling** - Health profile	I-13, I-15, I-21		I-10, I-13		I-27		3
**Profiling** - Health behaviors	I-8, I-13, I-15, I-17		I-10		I-4, I-7, I-8, I-20, I-27		3
**Health data** - physiological	I-1, I-2, I-3, I-7, I-8, I-13, I-17, I-18, I-24, I-25,		I-10	I-1, I-8, I-17, I-21, I-22, I-24, I-25	I-8, I-24	I-4	5
**Health data** - adherence	I-2, I-4, I-5, I-7, I-8, I-11, I-14, I-16, I-27			I-21	I-14	I-11, I-15	4
**Preferences**	I-1, I-13, I-15, I-6, I-17	I-1, I-9, I-11, I-15, I-16, I-19, I-21	I-24	I-14	I-27		5
**Contextual**	I-3, I-6, I-13, I-21	I-9	I-10, I-24		I-27		3
**System-derived**	I-1, I-2, I-3, I-8, I-13, I-17, I-18, I-25	I-17	I-10	I-1, I-18	I-2, I-4, I-7, I-24	I-4, I-15	6
**Socio-behavioral**	I-13	I-9	I-10				3
**Psychological**	I-8, I-15, I-17, I-21, I-22, I-26		I-10		I-4, I-7, I-8, I-20		3
**User action**	I-3, I-4, I-9, I-20, I-23	I-3	I-13			I-12	4
**Metadata**	I-2, I-3, I-4, I-6, I-7, I-8, I-13, I-15, I-16, I-17, I-21, I-26, I-27	I-9	I-10, I-13, I-24		I-7		4
n***	12	6	11	5	9	4	

*In some interventions, the content and the timing were tailored in a joint manner (I-10, I-13, I-24), which we added as a separate cluster here, as it was not possible to separate the variables.

** n = how many tailoring areas make use of a certain variable type.

*** n = how many variable types are used in each tailoring area

#### Tailoring approaches & variables specific to low-SEP factors.

**Fig 3:**
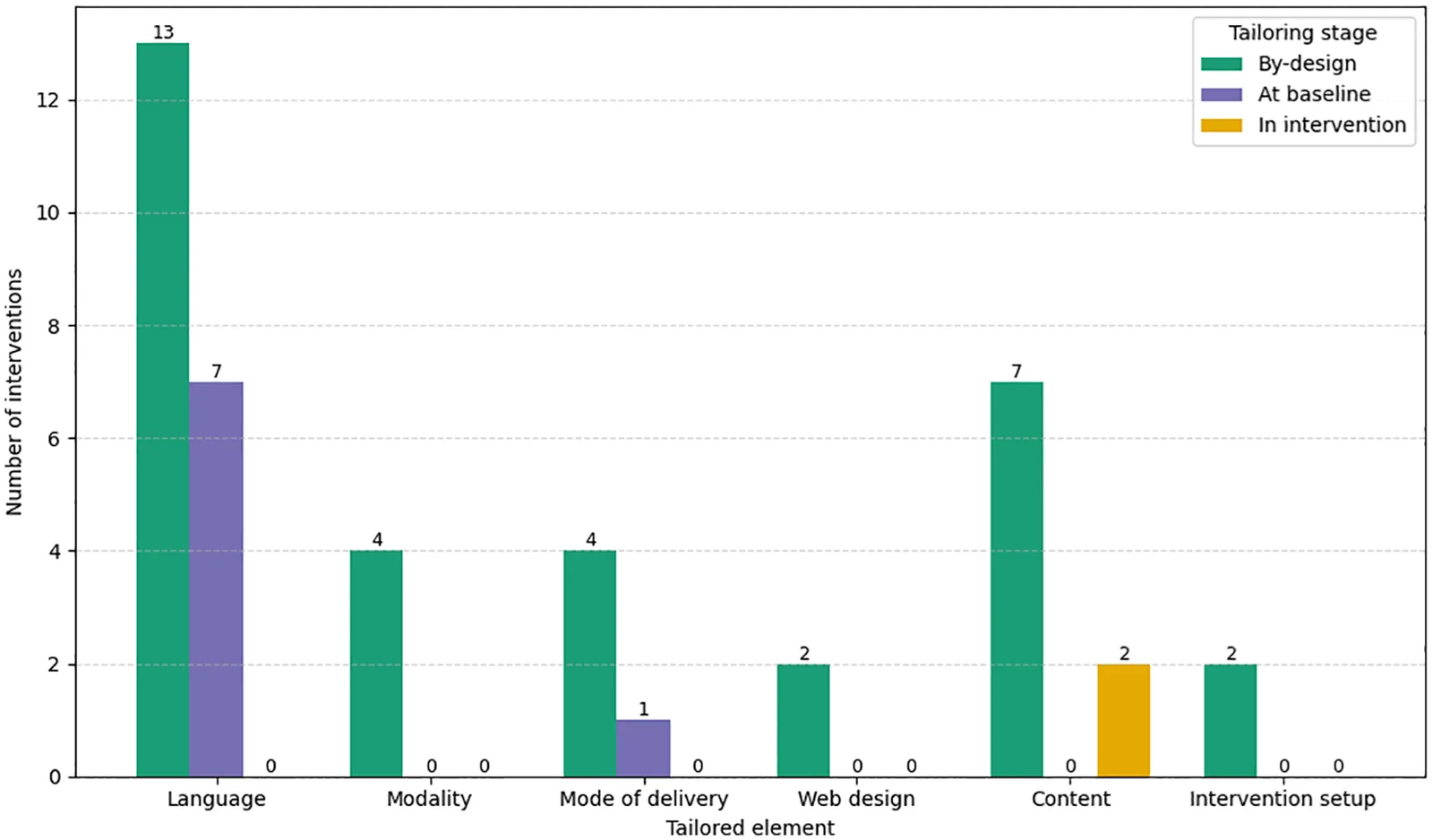
Visualization of Table 6: number of interventions tailoring low-SEP-related elements at different tailoring stages.

SEP-related variables were found to mostly determine the intervention’s design, e.g., to reduce requirements on literacy and numeracy skills. We identified six tailoring areas across twenty-four interventions (n = 24/27, 89%) that used SEP-related variables (e.g., health literacy or area of residence) to tailor the intervention (elements) (see **Table 6**). Most commonly, the tailoring was focused around translating or simplifying the language (n = 14/24, 58%) or enhancing sociocultural relatability via relevant content themes (n = 9/24, 38%) (see Fig 3).

**Table 6:** Overview of tailoring approaches and variables related to low-SEP factors.

Tailored element (n*)	Changes made	Stage of tailoring: Included tailoring variables
**Language (n = 14)** (I-4, I-10, I-11, I-13, I-14, I-15, I-16, I-17, I-18, I-20, I-21, I-23, I-25, I-26)	Translation into another language (e.g., Spanish (I-4, I-10, I-11, I-14, I-17, I-18, I-25)), simplifying the used language (I-10, I-16, I-20, I-23, I-26), adapting the length (I-16, I-20), the words and the tone of the content (I-15, I-16, I-21, I-26), to be suitable for the recipient’s language skills, but also culture or geographic area and community (I-13, I-15, I-21, I-26).	**At baseline:** Language (preferences) (I-4, I-10, I-11, I-14, I-17, I-18, I-25) **By-design tailored to:** Lower (health) literacy (I-11, I-13, I-16, I-20, I-23, I-26), lower reading level (I-10, I-16, I-20, I-21), culture/ethnicity and/or community (I-15, I-21, I-26)
**Modality (n = 4)** (I-4, I-5, I-8, I-18)	Using auditory or audio-visual modalities (solely or as an addition to text) to overcome literacy deficiencies (I-4, I-5, I-8). Visual materials (e.g., infographics (I-18)) were also used to support lower health literacy.	**By-design tailored to:** Low (health) literacy (I-4, I-5, I-8, I-18)
**Mode of delivery (n = 5)** (I-2, I-3, I-12, I-22, I-25)	Opting for SMS-messaging as technology accepted among the target population (I-2, I-3), native web app to circumvent Internet connection dependency (I-12), choosing web-based delivery to accommodate distance (I-22), or allowing the user to choose their resource-compatible mode of delivery (I-25).	**At baseline:** User preference on mode of delivery (I-25) **By-design tailored to:** Internet access, location, and/or technology acceptability (I-2, I-3, I-22, I-12)
**Web design (n = 2)** (I-19, I-20)	Simple web designs that are easy to navigate (I-19, I-20) and showing important information first (I-19).	**By-design tailored to:** Low digital literacy (I-19, I-20)
**Content (themes) (n = 9)** (I-6, I-8, I-10, I-12, I-13, I-19, I-21, I-24, I-27)	**Socioeconomic and cultural context:** Ensuring inter-personal relatability and cultural appropriateness (e.g., via provided visual materials, in terms of gender, appearance, cultural context or level of education (I-10, I-12, I-8), or via inclusion of certain types of PA, such as household chores (I-21)), taking into account costs for, e.g., food recommendations (I-13), focus on recommending PA resources that accommodate money and time available (I-27), or providing skill-training related to monetary spending strategies (I-19). **Location:** Recommendations based on availability of suggested food (I-13) and focus on increasing awareness of neighborhood PA resources (I-6, I-24).	**By-design tailored to:** Location and the socioeconomic, as well as cultural/ethnic background of the target population (I-8, I-10, I-12, I-13, I-19, I-21, I-27) **In-intervention tailoring:** *Contextual data*: Current location (I-6, I-24)
**Intervention setup (n = 2)** (I-12, I-23)	Inclusion of additional features or tools for additional support to health literacy (e.g., an action planning tool, additional BCTs (I-23), diabetes dictionary (I-12)).	**By-design tailored to:** Health literacy (I-12, I-23)

SMS, Short Message Service; PA, physical activity; BCTs, Behavioral Change Techniques.

*Number of interventions

In contrast to the overall design adaptations, seven (n = 7/24, 25%) interventions implemented tailoring at the start of the intervention (baseline), e.g., to let the participants choose their preferred language. Furthermore, two interventions (n = 2/24, 8%) used the participants’ current location for dynamic tailoring aimed at increasing the participants’ awareness of local exercise resources (I-6, I-24).

#### Levels of tailoring: Granularity + dynamic adaptivity.

**Fig 4:**
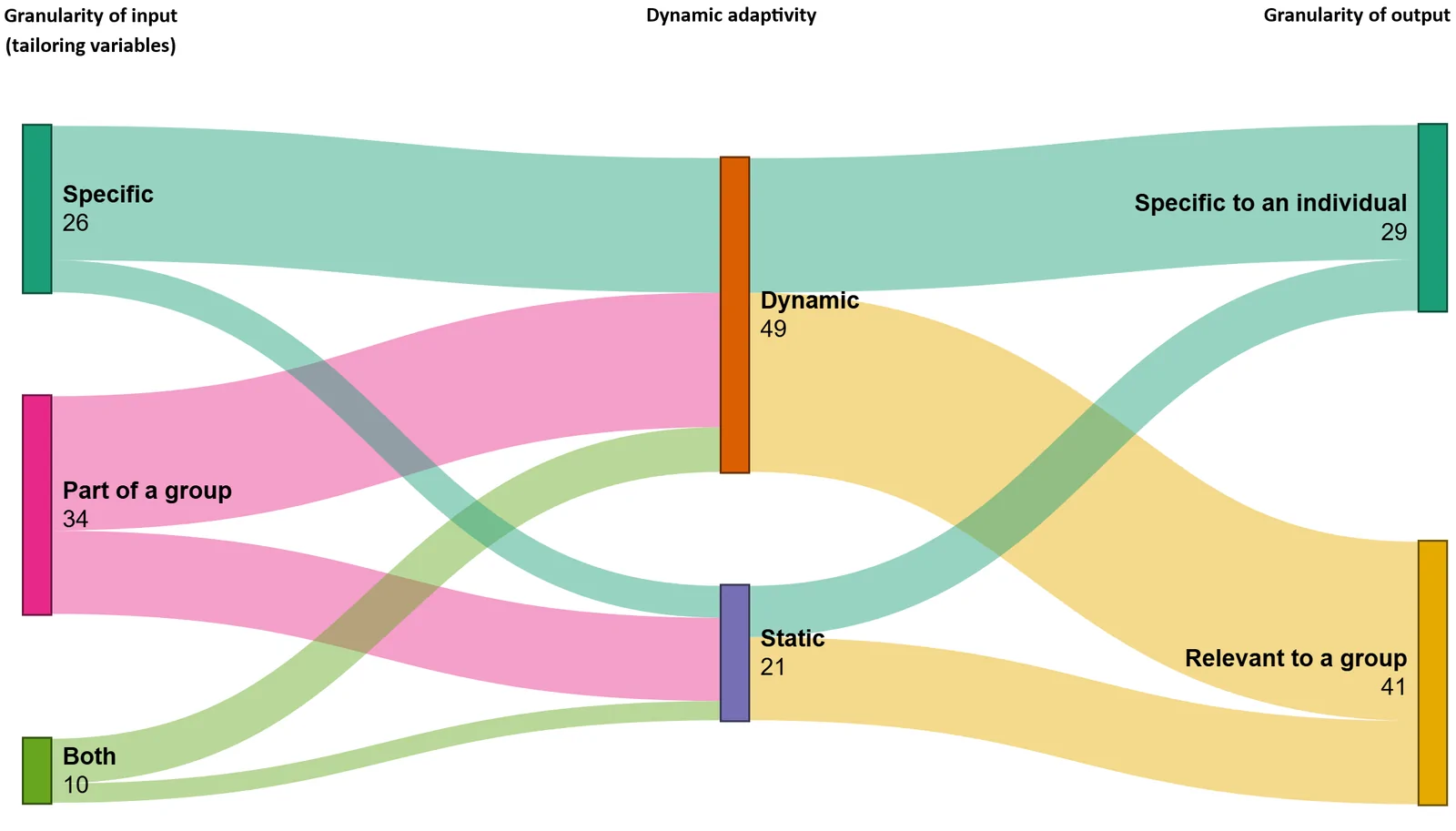
Tailoring approaches included within the interventions, mapped along the axes of granularity and dynamic adaptivity.

To further illustrate the levels of in-intervention tailoring adopted by the interventions, Fig 4 maps the input (tailoring variables) and output granularity, in relation to dynamic adaptivity (static or dynamic tailoring). Specific input was mostly fed into a specific output in the form of visual progress charts (I-1, I-8, I-17, I-21, I-22, I-24, I-25), through dynamic tailoring. In contrast, personal characteristics, such as name (I-15, I-16, I-19, I-21), enhanced the specificity of the output messages via static tailoring, being collected only once. Specific variables were sometimes further grouped into a more general input. For example, in I-4, the participants’ daily weight was relayed via a connected scale (specific input). At the end of the week, an average weight change was calculated and grouped into one of three zones. This zone then determined the coaching frequency (group-based output). Conversely, some interventions combined both types of variables in one tailoring approach (I-1, I-2, I-3, I-4, I-8, I-10, I-13, I-27), collecting, e.g., grouped variables at baseline and additional specific input (e.g., steps) continuously each day (I-10).

**Fig 5:**
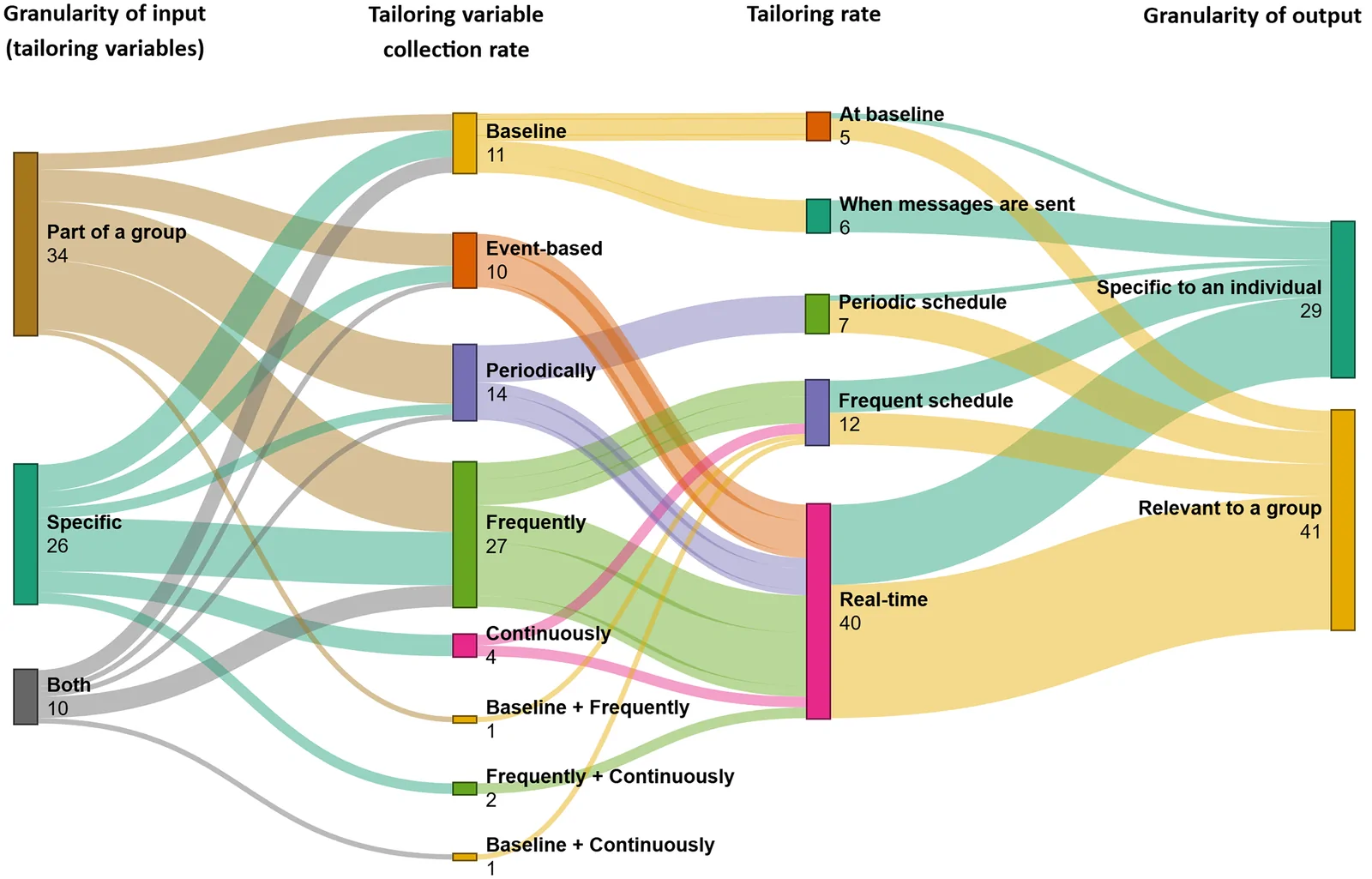
Tailoring approaches, mapped along the axes of granularity, the tailoring variable collection rate and the tailoring rate.

To further examine the aspect of dynamic adaptivity, our analyses of the variable collection and tailoring rates each produced five clusters, as shown in **Table 7**. While similar frequency patterns were found across the two rates, they were often un-matched, as shown in Fig 5.

**Table 7:** The identified sub-levels of how frequently the tailoring variables (input data) were collected across interventions and how frequently the output was tailored by the system in response to the collected variables.

Tailoring variable collection rate (n)	How frequently the tailoring variables are collected
**Baseline (13)**	Collecting input data using a single assessment at baseline.
**Event-based (10)**	Event-based collection rate, related to the user’s interaction with the intervention’s features, rather than a defined data collection pattern, e.g., giving a like to a message.
**Periodically (14)**	Collection on a periodic basis of longer time spans, such as monthly, or every couple of months.
**Frequently (30)**	More frequent collection, such as on a weekly or a daily basis, or at multiple points throughout the day. Could be combined with assessment at baseline.
**Continuously (7)**	Continuous collection, typically via devices (e.g., wearables) that can collect data on a minute-by-minute basis. Could be combined with assessment at baseline, or frequent variable collection.
**Tailoring rate (n)**	**How frequently tailoring occurs in response to the collected variables**
**At baseline (5)**	Only at the beginning of an intervention, e.g., by generating a tailored goal that does not change any further.
**When messages are sent (6)**	Each time a message is sent to the user, using variables that are collected at baseline and do not change.
**Periodic schedule (7)**	More infrequent time periods, e.g., on a monthly basis. The tailoring remains fixed in between these periods and is not immediately visible to the user.
**Frequent schedule (12)**	More frequent time points, such as on a weekly or daily basis. The tailoring is not immediately visible to the user.
**Real-time (40)**	An event-based, tailored system response to the user that occurs in real-time, e.g., to the user’s input.

Typically, variables were collected at a frequent rate with a real-time tailored output. For example, data on goal or behavior adherence was collected on a weekly basis and prompted an immediate, real-time feedback selection (I-2, I-3, I-5, I-11, I-14, I-16, I-27). This data could be group- or individual-based, however, the tailored output was more commonly group-based, as shown in Fig 5. Notably limited was continuous data collection, though some interventions tracked the participant’s data, such as steps (I-10, I-21, I-24) or GPS location (I-9, I-24) via their mobile phones or additional wearables.

Each intervention could make use of several tailoring approaches, with varying combinations in terms of granularity and dynamic adaptivity (see **S2 Table**). For example, in one intervention, the aim was to prioritize which messages are delivered to the user based on which messages they liked (I-9). Therefore, the users could provide a like/dislike on the received messages (event-based variable collection), which determined the system’s prioritization of messages (real-time response rate of the system). While the user-provided variable belonged to a group (like/dislike), the resulting output was specific to the individual, as the message prioritization was tailored to each person’s combination of indicated preferences. However, in both mappings displayed in Figs 4 and 5, a funnel structure was observed where choices in one layer constrained options in subsequent layers. For example, using group-based tailoring variables limited the production of specific output. A few exceptions were able to concatenate messages into a user-specific output (I-4, I-7, I-15), or guide message prioritization via user-given like/dislike (I-9). One such exception was the only case that combined group-based variables (collected via a survey) with static tailoring (once every three months) and a specific output. This was in the form of concatenated messages from various message ‘stems’, based on the participant’s answers to the survey items (I-15).

### Algorithms for tailoring

Regarding the algorithm functions used to execute in-intervention tailoring, we identified nine function clusters, as shown in **Table 8**. Feedback selection, variable substitution and content filtering/prioritization were used most often, while geofencing and adaptive goal-setting remained sparse. Four interventions (n = 4/27, 15%) used one function, e.g., content filtering (I-26), or feedback selection (I-5). In contrast, combining multiple functions was more common, with sixteen interventions (n = 16/27, 59%) using three functions or more, primarily combining feedback selection, progress calculation and variable substitution (n = 7/16, 44%) (I-1, I-2, I-4, I-7, I-10, I-17, I-27).

**Table 8:** Definitions of the identified thematic clusters for the functions of algorithms used for the execution of tailoring within the included eHealth interventions, inferred from the article’s intervention descriptions.

Types of functions (n*)	Function role
**Feedback selection (17)** (I-1, I-2, I-3, I-4, I-5, I-7, I-8, I-10, I-11, I-13, I-14, I-16, I-17, I-18, I-20, I-25, I-27)	Selection of appropriate feedback, typically based on user-provided (numerical) input to the system's inquiry on their goal progress or adherence.
**Variable substitution (16)** (I-1, I-2, I-4, I-6, I-7, I-10, I-13, I-15, I-16, I-17, I-18, I-19, I-21, I-23, I-24, I-27)	Insertion of relevant information, such as the user’s name, or information related to their goal, health or contextual data, typically within a message.
**Content filtering or prioritization (12)** (I-3, I-4, I-7, I-9, I-10, I-15, I-16, I-20, I-21, I-22, I-24, I-26)	Selection or filtering of content, based on, e.g., user’s characteristics or context. Typically, this was in the form of selecting a subset of relevant messages out of a larger message bank, or prioritization of a goal list, based on, e.g., the participant’s answers to a (recurring) survey, using rule-based methods. Other methods used reinforcement learning, pattern analysis from EMA data, or message ordering based on the participant’s feedback using like/dislike button on received messages.
**Progress calculation (10)** (I-1, I-2, I-3, I-4, I-7, I-8, I-10, I-17, I-25, I-27)	Comparison of (numerical) input (user-reported or device-captured) to, e.g., previous week’s values to calculate the user’s progress or adherence. This calculation was then typically used as a basis for providing feedback to the user.
**Structured Adaptive Logic (9)** (I-4, I-8, I-11, I-12, I-15, I-16, I-17, I-19, I-23)	The necessary tailoring was determined according to a rule-based flow, typically within a pre-defined structure, such as an action planning tool.
**Progress visualization (7)** (I-1, I-8, I-17, I-22, I-21, I-24, I-25)	Creating a visual representation of the participants’ progress towards the set goal in the form of graphs or figures.
**Recommendation provision (6)** (I-2, I-6, I-8, I-13, I-14, I-27)	Providing the users with (e.g., goal-related) advice, recommendations, or suggestions, typically based on contextual rules, available device-captured or system-retrieved data, or user input. Ultimately, the users could often decide themselves whether to follow the given recommendation.
**Geofencing (2)** (I-6, I-24)	Using a pre-set geofence around the user’s location to tailor the intervention to, e.g., nearby sports locations.
**Adaptive goal-setting (1)** (I-24)	Automatically setting the participant’s step goal based on historical device-captured step data.

EMA, Ecological Momentary Assessment.

*n of interventions that made use of a given function.

Two interventions explicitly mentioned the type of algorithm executing these functions: One intervention described the use of reinforcement learning (RL) for the content filtering function and feedback selection where the algorithm learned to select an appropriate type and timing of a message to the user (I-10). Another intervention described the use of a rule-based, ontology-enhanced recommender system for the recommendation provision function, generating, e.g., sports activity suggestions relevant to the user’s preferences or their current context (I-13). For the other interventions, we inferred the use of rule-based algorithms based on our interpretation of the algorithm descriptions in the respective articles. For example, Schultz et al. provided the rules used in their algorithm, although the authors called it a ‘workflow’ [92]. Some interventions (n = 9/27, 33%) provided the links to the used software, such as Qualtrics (I-3, I-8, I-9, I-10, I-16, I-20, I-23, I-26, I-27).

Further, we looked at which variable types were used as input for the tailoring functions in each intervention. The number of variable types used was highest (n = 12/12, 100%) in the content filtering/prioritization and recommendation provision functions and lowest in progress calculation and adaptive goal-setting (n = 2/12, 17%), as shown in **Table 9**. Some variable types were more frequently used for certain tailoring functions, e.g., progress calculation and visualization mainly used health data (such as steps or weight) to visualize and track the participants’ goals. On the other hand, all variable types were used for the content filtering/prioritization function, as the output of this function had a broader application, spanning from goal prioritization at baseline (I-4), to the daily selection of a message, based on a range of different variables (e.g., selected goal or current weather) (I-21). These patterns are similar to how the variable types were used across tailoring areas, aligning the usage of tailoring functions with areas of tailoring.

**Table 9:** Types of variables used in the interventions as input for the different tailoring functions.

	Feedback selection	Variable substitution	Content filtering/ prioritization	Progress calculation	Structured adaptive logic	Progress visualization	Recommendation provision	Geofencing	Adaptive goal-setting	n*
**Profiling** -Demographics		I-1, I-15, I-16, I-19, I-21, I-27	I-10, I-15, I-20				I-8			3
**Profiling** - Health profile		I-13	I-10, I-15, I-21				I-13, I-27			3
**Profiling** – Health behaviors			I-4, I-7, I-10, I-15, I-20		I-8, I-17		I-8, I-13, I-27			3
**Health data** - physiological	I-1, I-7, I-8, I-10, I-13, I-18	I-10, I-13, I-18, I-24	I-10, I-24	I-1, I-2, I-3, I-4, I-7, I-8, I-10, I-17, I-25		I-1, I-25, I-22, I-21, I-17, I-8, I-24	I-13, I-8		I-24	7
**Health data** - adherence	I-4, I-5, I-7, I-8, I-11, I-14, I-16, I-27		I-15	I-2, I-4, I-7, I-27	I-11	I-21	I-8, I-14			6
**Preferences**		I-1, I-13	I-9, I-15, I-21		I-15, I-16, I-17, I-19		I-6, I-13, I-27	I-24		5
**Contextual**		I-13	I-3, I-9, I-10, I-21				I-13, I-27	I-6, I-24		4
System-derived	I-1, I-2, I-3, I-8, I-10, I-13, I-17, I-18, I-25	I-13, I-18	I-4, I-7, I-10, I-15		I-4, I-17	I-1	I-2, I-13		I-24	7
**Socio-behavioral**			I-9, I-10				I-13			2
**Psychological**	I-8		I-4, I-7, I-10, I-15, I-21, I-20, I-22, I-26		I-8, I-17		I-8			4
**User action**	I-20	I-4, I-23	I-3, I-9		I-12, I-23		I-13			5
**Metadata**	I-4, I-13, I-27	I-2, I-4, I-6, I-7, I-13, I-16, I-17, I-27	I-3, I-7, I-9, I-10, I-15, I-16, I-21, I-26		I-8, I-17		I-13	I-24		6
n**	6	8	12	2	7	3	12	3	2	

*n = how many different functions use a certain variable type

**n = how many variable types each function uses.

### Evaluation

#### Evaluation phases.

The majority (n = 16/27, 59%) of the included interventions reached the *development* phase (TRL4 - TRL6) (see **Table 3** and **S3 Table**). Four (n = 4/27, 15%) interventions reached the *research* phase (TRL1 - TRL3), and three (n = 3/27, 11%) the *deployment* phase (TRL7 – TRL 9). Among the included interventions were four (n = 4/27, 15%) standalone protocols, for which we did not assign an evaluation phase.

#### Target vs. included population.

In most of the studies with evaluations (n = 23/28, 82%), participants from the low-SEP population with cardiometabolic conditions were included. In two studies (n = 2/28, 7%), the evaluation of the intervention was not conducted with the target users, but as a computer simulation of the target users’ behavior [55], or with digital personas evaluated by experts [65], focusing on early explorations of the intervention concepts. The recruitment of three studies (n = 3/28, 11%) in the development phase resulted in evaluation with participants of higher SEP who were more likely to join the study [61,71,81].

#### Evaluation of tailoring.

Twelve of the included interventions (n = 12/27, 44%) evaluated the tailoring within the tailored intervention elements, namely, the provided messages [61,68,79,85,88], recommendations [65], literacy-sensitive design [77,83], or (audio-)visual materials [59,71,76]. Most of these studies (n = 11/12, 92%) used qualitative evaluation methods, focusing on relevance [61,65,68,76,79,88] and appeal [71,77] of the intervention content. For example, to evaluate the appropriateness (e.g., in terms of language) of the generated content (e.g., messages), one study conducted focus groups with the target population [68], and two studies used an expert panel [65,88].

In some cases, (n = 3/12, 25%) qualitative evaluations were combined with quantitative analyses, assessing engagement and health literacy outcomes [77], and behavioral changes, such as healthy eating [59,83]. One study (n = 1/12, 8%) evaluated the effectiveness of the tailored messages using step count [85]. In two studies (n = 2/12, 17%), the participants commented on the tailoring that they noticed, or that could still be added, as part of the qualitative intervention evaluation [64,75]. Lastly, four evaluations (n = 4/12, 33%) compared the tailored intervention (elements) with a non-tailored version [59,77,83,85].

Within the qualitative evaluations, participants expressed a preference for short [61,76], simple and straightforward language [68,79] using layman terms [68] and positive framing [68,76]. Actionable advice with specific suggestions [61,68,75,79] and direct ties to health benefits [61,68], such as a specific number of steps to walk [68], or healthier versions of traditional recipes [75], was viewed positively. In contrast, certain suggestions, such as an inappropriate amount of exercise [65] or unrealistic food swaps (e.g., replace chips with an apple) [79], required further tailoring (e.g., by taking into account the users’ health data or preferences) to mitigate discouragement. Participants further expressed the need for more advanced tailoring to account for co-morbidities, as well as skill and knowledge level differences between newly diagnosed and long-term patients [75]. Additionally, the importance of sociocultural relatability of the content was highlighted [64,68,71,75,76,79,88], such as being able to recognize one’s own challenges in living with a chronic disease [64], using visuals relevant to a certain age group, or culture [75,76], terms familiar to a specific community [68,79,88], or citing opinions of local experts rather than national guidelines [68].

Within the quantitative studies, the benefits of using health literacy-sensitive design were reported, with a reduction in snacking behavior [83]. Additionally, one study showed that participants receiving messages tailored to include nearby exercise facilities were able to reach higher numbers of daily steps [85]. Participants also appreciated (audio-) visual materials [59,71,75,76], which led to higher intervention effectiveness [59].

#### Engagement.

Several (n = 5/27, 19%) of the interventions that evaluated tailoring also reported effects regarding engagement [64,71,75,77,85] (see **S3 Table**). One study reported an overall high engagement, which led to improved diabetes knowledge and higher confidence in performing diabetes self-care activities [64]. Two studies specifically highlighted the use of (audio-)visual materials [71] and health literacy-sensitive design [77] as having a positive effect on engagement. One study reported moderate engagement rates, with low usage of goal-setting tools [85]. Lastly, in one study, the lack of tailoring of the informational content to the participants’ pre-existing knowledge had a negative impact on engagement [75].

Eight other interventions (n = 8/27, 30%) [48,51,54,57,67,72,73,87], which did not evaluate tailoring, reported overall high engagement rates, which led to increased weight loss in two [54,57]. One study specifically found a link between higher engagement rates with the self-tracking tools and an improvement in eating habits and physical activity levels [72]. SMS text messages were also found to produce higher engagement rates over printed [51], as well as voice messages [67] where participants faced challenges, such as not understanding the instructions, the spoken language, or not being given enough time to respond to the voice message. Lastly, one study reported decreasing engagement due to participants losing interest in the provided content [48], and one study remained inconclusive on the role of high engagement metrics in improving biological outcomes [73].

## Discussion

**Fig 6:**
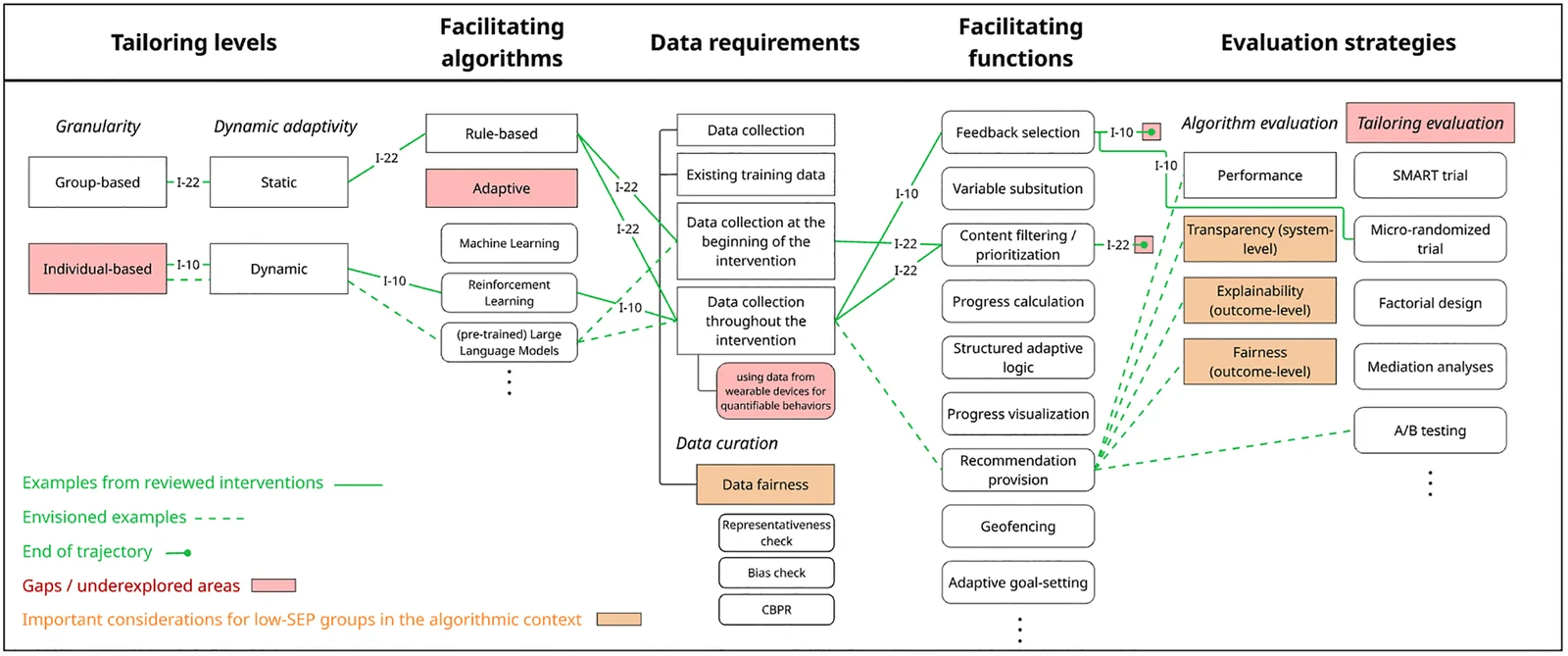
A conceptual framework, representing a synthesis of the results of this review, with research implications and envisioned future work. The red squares point to opportunities, such as further exploration of implementing user-based, adaptive algorithms as methods for tailoring, as well as the need for evaluations of different tailoring approaches. The orange squares highlight important considerations when designing with AI technologies for low-SEP groups. With the green lines, we included two examples from the reviewed interventions, to show how the existing interventions traverse this framework. We also included an envisioned example of which components a future intervention could contain. We aimed for representative example methods under, e.g., evaluations or adaptive algorithms, though these lists are not meant to be exhaustive. CBPR: Community-based participatory research.

This scoping review examined tailoring approaches within eHealth lifestyle interventions for people with lower SEP and cardiometabolic conditions. We identified a range of tailoring approaches with several commonalities: mainly self-reported tailoring variables (unrelated to SEP factors) were used (see **Table 4**) and low-SEP factors were primarily addressed with by-design, rather than in-intervention tailoring (see **Table 6**). Tailoring occurred dynamically in most cases, however, the input for tailoring and the tailored output were typically relevant to groups of users, rather than to individuals (see Fig 4). Most interventions relied on rule-based algorithms, using mainly functions of feedback selection, variable substitution and content filtering (see **Table 8**). The majority of the interventions were conducted at the development phase and an evaluation of tailoring was mostly lacking. We have synthesized the key concepts from our results, together with future research implications, which we discuss below, in a conceptual framework, shown in Fig 6. Our findings suggest that while tailoring is present in many interventions, its implementation and evaluation remain limited, especially for SEP-specific needs.

Starting with the tailoring levels, SEP-related tailoring was primarily present in the overall design, rather than within individualized adaptations, which was not always well received by participants. For example, in one of the interventions, participants expressed dissatisfaction with the uniform content aimed at low health literacy, which did not consider the varying knowledge levels between participants who were newly diagnosed, versus long-term patients [75]. Consequently, the participants’ motivation to engage in the intervention was negatively affected if the provided information was mostly already known to them and did not help them further advance their health-promoting (e.g., cooking) skills [75]. In such a scenario, tailoring during an intervention can better address changing circumstances. However, in other areas, a by-design approach might be sufficient and might even make the intervention suitable for both low- and high-SEP participants. For example, we found that simple language, free of clinical jargon, was important for low-SEP participants [68,79] – such a design might, however, be equally valuable for a large part of the general population. Similarly, a study on lifestyle perceptions among people of varying SEP found that while some attitudes towards healthy eating, such as cost-related reluctance towards dietary changes, were more specific to low SEP, some were also shared across both low and high SEP participants [93]. Thus, future studies should determine which variables can guide the intervention design for all participants and which bring more benefits when used for individualized tailoring. Such distinctions will help to guide the selection of tailoring approaches that are able to address within-group diversity, but also enable use of the intervention across varying SEP groups.

The tailoring variables were mostly collected via user-reports, also when it comes to, e.g., physical activity levels. Previous reviews on personalized eHealth and mHealth interventions among the general population have reported similar results, with a minority of the included studies using automatically captured data [94–96]. While such an approach removes the need for additional (wearable) devices, which might not be readily accessible to low-SEP populations, it can also create several limitations. First, self-reporting quantitative data reduces data objectivity [97,98]. Two of the included interventions reported that participants struggled with estimation of their physical activity levels [59,76], and some participants did not receive the advice to increase their physical activity levels due to overestimated self-reports [59]. Second, self-reported data impacts the granularity and dynamic adaptivity of tailoring. Limited collection of specific data restricts the granularity of the output that can be generated, as also shown in our results. The majority of the interventions applied dynamic tailoring, however, due to reliance on manual user input, the tailoring was typically fixed to a pre-set interval, rather than occurring at the time of the user’s need. In another review on weight-loss eHealth interventions for the general population, a similar frequency of dynamic tailoring was found among the included studies, with input being collected on a daily or weekly basis [96]. Lastly, self-reporting might be especially inappropriate for low-SEP users, as evidence shows that socioeconomic context influences self-report inaccuracies [99]. Taken together, user-reporting seems to be used more often, potentially due to the lower implementation barrier. However, this might be especially inappropriate for low-SEP users and can limit the intervention effectiveness [100]. Future interventions should focus on device-captured data for quantifiable behaviors to ensure accurate behavioral estimations and appropriate system response for maximized tailoring benefits. This would allow to increase the data specificity and volume that could be fed into adaptive algorithms for a more user-based, adaptive tailoring, while reducing user burden.

For the algorithms executing the tailoring, we found that most interventions seemed to rely on rule-based algorithms. Such an approach remains prevalent in the existing eHealth literature, as also shown in other reviews on just-in-time adaptive interventions [101] or digital mental health interventions [102] targeting the general population. Rule-based methods can circumvent training data requirements of other methods, more easily incorporate clinical guidelines [103,104], and provide more straightforward and transparent decision-making. Moreover, the sophistication of rule-based systems lies on a continuum, ranging from simple if-then rules, to large decision trees with complex branching logic. However, they still pose challenges, such as rule construction, which can become complex when adapting to multiple variables, or variables of idiosyncratic nature (e.g., user preferences). For more granular and dynamic tailoring, artificial intelligence (AI)-based algorithms provide greater flexibility. AI-based methods, leveraging, e.g., RL, can incorporate many tailoring variables simultaneously and extract data patterns to continuously adapt to the user’s actions or circumstances [105,106]. This is also evident in our results where one intervention used RL with almost all tailoring variable types to jointly tailor content and timing [62]. This means that interventions can draw on more diverse data types and sources (e.g., behavioral, contextual, feedback, preferences) to extend their tailoring capabilities and limit additional manual development of rule-based conditions. Similarly, adaptive recommender systems could curate content (themes) within the intervention, which was one of the largest areas for low-SEP-related tailoring. Using information, such as health status, barriers, cultural background, or self-efficacy to adapt the content selection, also if changes occur over time, could help to remove friction and address specific needs of each user. Since these methods require training data, which are not always available or abundant, models that can work with smaller amounts of data, or that can be trained dynamically on data collected during the intervention, may be preferred in the eHealth context. These can also be embedded within rule-based systems to support simpler tailoring with straightforward decision-making (e.g., evaluating whether a goal has been reached) complemented with more adaptive, data-driven tailoring where possible (e.g., adaptive goal-setting). When choosing the adaptive system architecture, the varying data, transparency, and evaluation requirements must always be considered, as also highlighted in Fig 6. Future work should explore this gap further to delineate the possibilities and requirements for implementing adaptive algorithms within this context.

The recent paradigm shift in the competence of large language models (LLMs), such as ChatGPT, has enabled automatic content generation, widening the scope of possibilities for future tailoring approaches even further. For example, creating content that is directly tailored to an individual can be challenging, but LLMs can leverage user-specific information to generate fully individualized content. This includes real-time generation of text and/or images, supporting functions of feedback selection, variable substitution, or recommendation provision [107,108]. LLMs could facilitate low-SEP-specific tailoring through, e.g., user profiles or interactive collaboration that moves away from by-design approaches. For example, adapting language complexity, length or tone of text to match language skills, or community preferences, which are important tailoring aspects for this target population. With the rapid LLM development, many pre-trained LLM models can nowadays be used off-the-shelf with some additional finetuning, reducing the resource and data requirements for their usage. To be able to implement LLMs for tailoring, further research is required to ensure their trustworthiness in the eHealth context. Future work should focus on delineating appropriate LLM guardrails that mitigate inaccurate information and ensure that it remains transparent and within approved medical guidelines. With such insights, this area of research holds a promising direction for more sophisticated tailoring methods, especially for low-SEP-relevant adaptations.

However, it is important to consider that data from vulnerable populations are frequently underrepresented or missing from the datasets used to train AI-based models, which can further augment existing societal biases [109]. To address this issue, first, co-design and community-based participatory research should be leveraged for intervention design, with community members being involved as a part of the research team. This will ensure that the developed AI tools are bias-free and sufficiently adapted to the needs and preferences of the target group. Next, the data that is used for AI model training should first be evaluated for representativeness of the target group and for any existing biases. When limited or no training data is available, additional data collection methods should serve the purpose of (re-)training models in subsequent trials or trial iterations. Using wearables is especially important, to include the low-SEP group in the data infosphere, from which they are often missing due to the lack of real-world use of digital devices [110]. Lastly, the performance and explainability of the developed model(s) must be evaluated on the target sub-group data to ensure sufficient transparency. Subsequently, computational evaluations should be complemented with community/user evaluations to validate the system’s performance and fairness in its tailored output.

Regarding the evaluation, we found positive effects of tailoring on user perceptions, engagement and intervention outcomes. However, evaluation of tailoring was missing from most interventions. This is a persistent gap in eHealth, also identified in other reviews on eHealth interventions for the general population [111–113]. Although the results of this review, as well as existing literature, show that tailoring holds promise for increasing intervention effectiveness [32,114], more evidence is still needed to understand the appropriateness and impact of different tailoring approaches [112,113]. For example, most of the interventions focused on qualitative evaluation, and only a few compared the tailored intervention (elements) against non-tailored ones. While qualitative judgments can provide valuable insights into users’ perceptions, comparative evaluations can additionally aid in separating the effects of tailoring mechanisms from the effects of the intervention as a whole. For example, to isolate the effects of tailoring, the comparator should not be a fundamentally different intervention. Oftentimes, tailored interventions are compared against routine care, which obscures the exact value that tailoring contributes to the overall effectiveness of the new intervention. Rather, the tailored intervention should also be compared against a non-tailored counterpart, next to routine care. In this way, a baseline effect can be determined of the new approach over established procedures, as well as what added effects the tailoring provides. Thus, future research should focus on evaluating tailoring approaches, for example via micro-randomized trials [115], by comparing the tailored system to a non-tailored one with, e.g., a factorial trial design, or A/B testing of tailored variants. An important metric to be included in such evaluations is engagement. Engagement may improve intervention effectiveness, thus insights into effective tailoring approaches, as well as potential engagement barriers that could be solved with tailoring are crucial to evaluate and report. Together with more comprehensive reporting on the used tailoring algorithms, these steps can ensure reproducibility and facilitate stronger analyses of the outcomes of different tailoring methods.

### Strengths and limitations

This review provides a thorough overview of tailoring approaches in eHealth interventions targeting low-SEP users. We approached the topic systematically and from multiple perspectives, drawing on articles with various methodological designs, which allowed us to gain a better understanding of the nature of the existing research. To the best of our knowledge, no review has yet synthesized tailoring approaches for this target group, which makes a valuable contribution to this area of research. Moreover, our review contributes to the Sustainable Development Goals (SDGs) by investigating and promoting appropriate tailoring approaches within eHealth lifestyle interventions. We contribute to the SDGs #3 “Good health and well-being” (target 3.4) and #10 “Reduced inequalities” (targets 10.2) by bringing awareness to the existing tailoring approaches and where improvements can be made. Our work can be used to increase the effectiveness and SEP-related relevance of eHealth interventions, promoting reduction of non-communicable disease-related mortality (SDG #3) and inclusion and participation of low SEP groups (SGD #10).

However, the results should be considered in the light of several limitations. Firstly, the search string used to identify eligible studies was rather complex, to simultaneously keep the search broad, but limit the indexing of irrelevant studies. Such an approach could have resulted in some studies being missed and not included in our review. However, the searches in registers for sister papers and the backwards snowballing, which did not identify any additional interventions, have increased our confidence that all relevant articles have been included.

Secondly, a strength of our paper is that we have also examined the algorithms that executed tailoring within the eHealth interventions, which provides insight into the technology underlying the tailoring mechanisms. However, the reporting on the used algorithms within the included articles often lacked explicit characterization, such as directly naming an algorithm as rule-based, so we had to infer the algorithm type or function from the provided descriptions. This means that certain functions of the algorithms may have been missed where not mentioned, not shown (e.g., in example messages), or lacking a comprehensive description.

Thirdly, four of the included studies were standalone protocols without evaluation. This could mean that the tailoring approaches within these protocols were more ambitious and might not be feasible to implement in a real-world scenario. Nevertheless, these studies showcase the intended tailoring and might serve as an inspiration for future work.

Lastly, the screening and coding process was primarily conducted by one reviewer (SVM), which might have introduced biases in the decision-making process. However, we implemented several mitigating measures, such as partial (20%) double screening and in each step, any doubts or discrepancies were discussed with the other co-authors until consensus was reached. This strengthens the reliability of our findings, despite limited reviewer triangulation.

## Conclusion

This review provides a synthesis of tailoring approaches used in eHealth interventions targeting primarily diet and exercise in low-SEP populations with cardiometabolic conditions. We found that tailoring was most often dynamic and targeted groups of users, using functions such as feedback selection and variable substitution. The evaluations seem to suggest a positive impact of health literacy-sensitive design and highlight the value of (audio-)visual materials and sociocultural relevance. The overview of tailoring levels, tailoring variables, and participant perspectives provides useful pointers for design of diet- and exercise-focused interventions with tailoring for low-SEP populations. However, the findings also uncover several gaps and opportunities for further research. Firstly, the results showed that the technology used for executing the tailoring within the interventions mainly relied on rule-based algorithms. Such an approach can circumvent various challenges posed by other, more complex algorithms; however, it might also limit the possibilities for tailoring. Secondly, we identified a lack of evaluation of tailoring, which was missing in most interventions. This limits the conclusions that can be made about the appropriateness and effectiveness of the different tailoring approaches. Evaluations of tailoring would help us better understand how individually driven the eHealth interventions truly need to be and to guide the selection of the technology that executes tailoring. Moreover, evaluations can facilitate data-driven tailoring from collected data and can contribute to reducing the data disparity problem by creating more inclusive datasets for AI-based models. These gaps highlight a potential discrepancy between the available technology and the solutions implemented, but also a need for evaluations of the used tailoring approaches. To guide future interventions, we assembled the key identified gaps and concepts from our review into a visionary framework, to help expand the existing tailoring approaches and to highlight key considerations in the design process of tailoring. Future research should explore the integration of adaptive algorithms to improve the tailoring process. However, this must be accompanied by rigorous evaluation methods and trial designs, such as factorial design, that can differentiate the specific impact of tailoring from the overall effectiveness of eHealth interventions. For this, standardization of evaluation processes to appraise tailoring approaches emerges as a productive avenue for future research.

## Supporting information

S1 Table PRISMA-ScR checklist. From: Tricco AC, Lillie E, Zarin W, O'Brien KK, Colquhoun H, Levac D, et al. PRISMA Extension for Scoping Reviews (PRISMAScR): Checklist and Explanation. Ann Intern Med. 2018;169:467–473. https://doi.org/10.7326/M18-0850.
(PDF)

S1 Text Search string. (DOCX)

S2 Table Tailoring areas, levels and variables with facilitating tailoring functions, per intervention. (DOCX)

S3 Table Categorization of each evaluation study into the evaluation phases of research, development, and deployment and the used engagement metrics. (DOCX)
