# Supplementary material for: Tailoring in eHealth lifestyle interventions targeting people with cardiometabolic conditions and lower socioeconomic position: A scoping review
Source: PLOS Digit Health. 2026 Sep 11;5(9):e0001713. doi: 10.1371/journal.pdig.0001713 (PMC13568521; doi:10.1371/journal.pdig.0001713)
Supplement: Supplementary file 2 [file pdig.0001713.s002.docx]

# **S1 Text. Search String.** Full Electronic Search Strategy

# Search strategy per database

**Proximity operators (PO)** used for: Scopus, Web of science, PsychInfo

- Guide for the size of N^[1]^ (in NEAR/N or W/N):
  - same phrase, N = 3 to 5
  - same sentence, N = 25

## Scopus

- Date of search: 03-05-2024
- Keywords: See table below
- Searching within: TITLE-ABS-KEY (Article title, Abstract, Keywords)
- Booleans between the search clusters: AND
- Search filters:
  - Range of year: 2000 - 2024
  - Language: English
  - Exclusion filters: Review (“re”), Note (“no”), Book chapter (“ch”), Conference review (“cr”), Editorial (“ed”), Erratum (“er”)

| **Scopus** | | | |
| --- | --- | --- | --- |
| **Key constructs** | **Cluster #** | **Keywords** | **Results** |
| Low socioeconomic position | #1 | ( TITLE-ABS-KEY ( ( ( low OR lower ) W/5 ( socio-economic OR "socio economic" OR socioeconomic ) ) OR ( ( socio-economic OR "socio economic" OR socioeconomic ) W/5 ( position OR status OR disparity OR inequality ) ) OR "SES" OR "SEP" OR "social determinants of health" OR ( health AND ( disparity OR inequality OR discrepancy ) ) OR ( ( deprived OR disadvantaged OR vulnerable OR impoverished OR underprivileged ) W/5 ( neighbourhood* OR neighborhood* ) ) OR rural OR ( low-income OR "low income" OR limited-income OR "limited income" OR "lower-middle income" ) OR "low-educated" OR ( ( low OR lower ) W/5 ( "economic status" OR educated OR education OR "education level" OR literacy OR "health literacy" OR "digital literacy" OR "digital health literacy" OR numeracy OR "computer literacy" OR "media literacy" OR "science literacy" OR "information literacy" OR "eHealth literacy" ) ) ) AND NOT TITLE-ABS-KEY ( ( child* OR adolescent* ) ) ) | 766.170 |
| eHealth lifestyle intervention | #2 | ( TITLE-ABS-KEY ( ( mhealth OR ehealth OR e-health OR m-health OR "mobile health" OR "electronic health" OR e-coaching OR ecoaching OR telemedicine OR "personal health informatics" OR "health promotion" OR "recommender system" OR "recommendation system" OR "recommender systems" OR "recommendation systems" ) OR ( ( mhealth OR ehealth OR telemonitoring OR mobile-based OR "mobile based" OR app-based OR "app based" OR computer-based OR "computer based" OR web-based OR "web based" OR internet-based OR "internet based" OR "digital health" OR health OR mobile OR smartphone OR smartwatch OR wearable OR computer OR online OR hybrid OR digital OR behaviour* OR behavior* OR ( ( behaviour* OR behavior* ) W/5 ( change OR modification ) ) OR lifestyle ) W/25 ( intervention* OR program* OR coaching OR coach OR telemonitoring OR technology OR app OR apps OR application* OR "health coaching" OR recommendation* OR feedback OR notification* OR message* OR text-message* OR "text message" OR "text messages" OR reminder* OR prompt* ) ) OR ( blended-care OR "blended care" OR "blended coaching" ) ) ) | 3.243.493 |
| Tailoring / personalisation | #3 | ( TITLE-ABS-KEY ( ( tailored OR tailoring OR personalised OR personalized OR personalisation OR personalization OR personalising OR personalizing OR adaptive OR persuasive OR context-aware OR "context aware" OR "context awareness" OR computer-tailored OR jitai OR "just-in-time" OR "just in the moment" OR "content matching" OR "content-matching" OR "source matching" OR "user targeting" OR "goal setting" OR "adaptive goal setting" ) OR ( ( mobile OR dynamic OR "context based" OR context-based OR context OR computer-based OR computer OR web-based OR internet-based OR digital OR app-based OR person-specific OR "person specific" OR user-based OR individual-based OR cluster-based OR group-driven OR group-based OR community-based OR community-level ) W/25 ( tailoring OR tailored OR personalised OR personalized OR personalisation OR personalization OR personalising OR personalizing ) ) ) ) | 1.660.083 |
| Lifestyle domains | #4 | ( TITLE-ABS-KEY ( ( "physical activity" OR exercise OR exercising OR movement OR workout* OR training* OR "active lifestyle" OR ( ( activity OR sedentary ) W/5 ( behaviour OR behavior ) ) OR nutrition OR diet OR ( health* W/5 ( eating OR diet ) ) OR "dietary habits" OR "dietary regimen" OR ( ( vegetable OR fruit OR food ) W/5 ( intake OR consumption ) ) OR ( ( eating OR sleeping OR sleep ) W/5 habits ) OR nourishment OR "weight loss" OR sleep* OR bedtime OR stress OR "chronic stress" ) ) ) | 8.873.942 |
| Cardiometabolic conditions | #5 | ( TITLE-ABS-KEY ( ( "metabolic syndrome" OR "metabollic syndrome" OR pre-diabet* OR prediabet* OR ( ( cardiovascular OR cardiometabolic ) W/5 ( disease OR risk* ) ) OR cvd OR diabet* OR t2d OR t2dm OR hyperglycemia OR hyperglycaemia OR ( high W/5 ( "glycemic index" OR "glycaemic index" ) ) OR "impaired glucose metabolism" OR "glucose metabolism disorder" OR dysglycemia OR "insulin resistance" OR obes* OR overweight OR dyslipidemia OR triglycerides OR ( ( high* OR increased OR elevated ) W/5 ( "body weight" OR "body mass index" OR bmi OR "glycated hemoglobin" OR "blood pressure" OR cholesterol OR "blood glucose" ) ) OR hypertension ) ) AND NOT TITLE-ABS-KEY ( ( "gestational diabetes" OR pregnant OR pregnancy OR "mental disorder" OR "mental illness" ) ) ) | 3.050.653 |
| Resulting query | #6 | #1 AND #2 AND #3 AND #4 AND #5 | 555 |
| **Resulting query with added filters** | **#7** | **#1 AND #2 AND #3 AND #4 AND #5 AND PUBYEAR > 1999 AND PUBYEAR < 2025 AND ( EXCLUDE ( DOCTYPE , "re" ) OR EXCLUDE ( DOCTYPE , "no" ) OR EXCLUDE ( DOCTYPE , "cr" ) OR EXCLUDE ( DOCTYPE , "ch" ) OR EXCLUDE ( DOCTYPE , "ed" ) OR EXCLUDE ( DOCTYPE , "er" ) ) AND ( LIMIT-TO ( LANGUAGE , "English" ) )** | **456** |
| **Update search** |  |  | **554** |

## Web of Science

- Date of search: 03-05-2024
- All Databases
- Keywords: See table below
  - *Query options: Exact search*
- Searching within: TS = Topic [Title, Abstract, Keywords]
- Booleans between the search clusters: AND
- Search filters:
  - Range of year: 2000 - 2024
  - Language: English
  - Exclusion filters: Dissertation Thesis, Review Article, Meeting, Abstract, Biography, Letter, Editorial Material (Document Types), and Awarded Grants

| **Web of Science** | | | |
| --- | --- | --- | --- |
| **Key constructs** | **Cluster #** | **Keywords** | **Results** |
| Low socioeconomic position | #1 | (TS=(((low OR lower) NEAR/5 (socio-economic OR “socio economic” OR socioeconomic)) OR ((socio-economic OR “socio economic” OR socioeconomic) NEAR/5 (position OR status OR disparity OR inequality)) OR “SES” OR “SEP” OR “social determinants of health” OR (health AND (disparity OR inequality OR discrepancy)) OR ((deprived OR disadvantaged OR vulnerable OR impoverished OR underprivileged) NEAR/5 (neighbourhood* OR neighborhood*)) OR rural OR (low-income OR “low income” OR limited-income OR “limited income” OR “lower-middle income”) OR “low-educated” OR ((low OR lower) NEAR/5 (“economic status” OR educated OR education OR “education level” OR literacy OR “health literacy” OR “digital literacy” OR “digital health literacy” OR numeracy OR “computer literacy” OR “media literacy” OR “science literacy” OR “information literacy” OR “eHealth literacy”)))) NOT TS=((child* OR adolescent*)) | 1.328.806 |
| eHealth lifestyle intervention | #2 | (TS=((mHealth OR ehealth OR e-health OR m-health OR “mobile health” OR “electronic health” OR e-coaching OR eCoaching OR telemedicine OR “personal health informatics” OR “health promotion” OR “recommender system” OR “recommendation system” OR “recommender systems” OR “recommendation systems”) OR ((mhealth OR ehealth OR telemonitoring OR mobile-based OR “mobile based” OR app-based OR “app based” OR computer-based OR “computer based” OR web-based OR “web based” OR internet-based OR “internet based” OR “digital health” OR health OR mobile OR smartphone OR smartwatch OR wearable OR computer OR online OR hybrid OR digital OR behaviour* OR behavior* OR ((behaviour* OR behavior*) NEAR/5 (change OR modification)) OR lifestyle) NEAR/25 (intervention* OR program* OR coaching OR coach OR telemonitoring OR technology OR app OR apps OR application* OR “health coaching” OR recommendation* OR feedback OR notification* OR message* OR text-message* OR “text message” OR “text messages” OR reminder* OR prompt*)) OR (blended-care OR “blended care” OR “blended coaching”))) | 3.097.440 |
| Tailoring / personalisation | #3 | (TS=((tailored OR tailoring OR personalised OR personalized OR personalisation OR personalization OR personalising OR personalizing OR adaptive OR persuasive OR context-aware OR “context aware” OR “context awareness” OR computer-tailored OR JITAI OR “just-in-time” OR “just in the moment” OR “content matching” OR “content-matching” OR “source matching” OR “user targeting” OR “goal setting” OR “adaptive goal setting”) OR ((mobile OR dynamic OR “context based” OR context-based OR context OR computer-based OR computer OR web-based OR internet-based OR digital OR app-based OR person-specific OR “person specific” OR user-based OR individual-based OR cluster-based OR group-driven OR group-based OR community-based OR community-level) NEAR/25 (tailoring OR tailored OR personalised OR personalized OR personalisation OR personalization OR personalising OR personalizing)))) | 1.614.819 |
| Lifestyle domains | #4 | (TS=((“physical activity” OR exercise OR exercising OR movement OR workout* OR training* OR “active lifestyle” OR ((activity OR sedentary) NEAR/5 (behaviour OR behavior)) OR nutrition OR diet OR (health* NEAR/5 (eating OR diet)) OR “dietary habits” OR “dietary regimen” OR ((vegetable OR fruit OR food) NEAR/5 (intake OR consumption)) OR ((eating OR sleeping OR sleep) NEAR/5 habits) OR nourishment OR “weight loss” OR sleep* OR bedtime OR stress OR “chronic stress”) )) | 12.369.823 |
| Cardiometabolic conditions | #5 | (TS=((“metabolic syndrome” OR “metabollic syndrome” OR pre-diabet* OR prediabet* OR ((cardiovascular OR cardiometabolic) NEAR/5 (disease OR risk*)) OR CVD OR diabet* OR T2D OR T2DM OR hyperglycemia OR hyperglycaemia OR (high NEAR/5 (“glycemic index” OR “glycaemic index”)) OR “impaired glucose metabolism” OR “glucose metabolism disorder” OR dysglycemia OR “insulin resistance” OR obes* OR overweight OR dyslipidemia OR triglycerides OR ((high* OR increased OR elevated) NEAR/5 (“body weight” OR “body mass index” OR BMI OR “glycated hemoglobin” OR “blood pressure” OR cholesterol OR “blood glucose”)) OR hypertension) )) NOT TS=((“gestational diabetes” OR pregnant OR pregnancy OR “mental disorder” OR “mental illness”)) | 3.536.192 |
| Resulting query | #6 | #1 AND #2 AND #3 AND #4 AND #5 | 1.299 |
| **Resulting query with added filters** | **#7** | **#1 AND #2 AND #3 AND #4 AND #5 *** | **424** |
| **Update search** |  |  | **502** |

**Timespan* (as a separate filter): *2000-01-01 to 2024-05-03 (Publication Date)*

*Other separate filters:* ***Awarded Grant*** *or* ***Dissertation Thesis*** *or* ***Review Article*** *or* ***Meeting*** *or* ***Abstract*** *or* ***Editorial Material*** *or* ***Biography*** *or* ***Letter*** *(Exclude – Document Types) and* ***English*** *(Languages)*

## PubMed

- Date of search: 03-05-2024
- Searching within: MeSH (Medical Subject Heading), [Title/Abstract]
- Keywords: See Table below
- Booleans between the search clusters: AND
- Search filters:
  - Range of years: 2000 - 2024
  - Language: English
  - Exclusion filters: -

| **PubMed** | | | |
| --- | --- | --- | --- |
| **Key constructs** | **Cluster #** | **Keywords** | **Results** |
| Low socioeconomic position | #1 | (("Socioeconomic Factors"[Mesh] OR "Healthcare Disparities"[Mesh] OR "Rural Population"[Mesh] OR "Socioeconomic Disparities in Health"[Mesh]) OR (((low[Title/Abstract] OR lower[Title/Abstract]) AND (socio-economic[Title/Abstract] OR "socio economic"[Title/Abstract] OR socioeconomic[Title/Abstract])) OR ((socio-economic[Title/Abstract] OR "socio economic"[Title/Abstract] OR socioeconomic[Title/Abstract]) AND (position[Title/Abstract] OR status[Title/Abstract] OR disparity[Title/Abstract] OR inequality[Title/Abstract])) OR "SES"[Title/Abstract] OR "SEP"[Title/Abstract] OR "social determinants of health"[Title/Abstract] OR (health[Title/Abstract] AND (disparity[Title/Abstract] OR inequality[Title/Abstract] OR discrepancy[Title/Abstract])) OR ((deprived[Title/Abstract] OR disadvantaged[Title/Abstract] OR vulnerable[Title/Abstract] OR impoverished[Title/Abstract] OR underprivileged[Title/Abstract]) AND (neighbourhood*[Title/Abstract] OR neighborhood*[Title/Abstract])) OR rural[Title/Abstract] OR (low-income[Title/Abstract] OR "low income"[Title/Abstract] OR limited-income[Title/Abstract] OR "limited income"[Title/Abstract] OR "lower-middle income"[Title/Abstract]) OR "low-educated"[Title/Abstract] OR ((low[Title/Abstract] OR lower[Title/Abstract]) AND ("economic status"[Title/Abstract] OR educated[Title/Abstract] OR education[Title/Abstract] OR "education level"[Title/Abstract] OR literacy[Title/Abstract] OR "health literacy"[Title/Abstract] OR "digital literacy"[Title/Abstract] OR "digital health literacy"[Title/Abstract] OR numeracy[Title/Abstract] OR "computer literacy"[Title/Abstract] OR "media literacy"[Title/Abstract] OR "science literacy"[Title/Abstract] OR "information literacy"[Title/Abstract] OR "eHealth literacy"[Title/Abstract])))) NOT ((child*[Title/Abstract] OR adolescent*[Title/Abstract])) | 731.889 |
| eHealth lifestyle intervention | #2 | **(**Telemedicine[Mesh] OR "Mobile Applications"[Mesh] OR "Digital Health"[Mesh] OR "Algorithms"[Mesh] OR "Digital Technology"[Mesh]) OR ((mHealth[Title/Abstract] OR ehealth[Title/Abstract] OR e-health[Title/Abstract] OR m-health[Title/Abstract] OR "mobile health"[Title/Abstract] OR "electronic health"[Title/Abstract] OR e-coaching[Title/Abstract] OR eCoaching[Title/Abstract] OR telemedicine[Title/Abstract] OR "personal health informatics"[Title/Abstract] OR "health promotion"[Title/Abstract] OR "recommender system"[Title/Abstract] OR "recommendation system"[Title/Abstract] OR "recommender systems"[Title/Abstract] OR "recommendation systems"[Title/Abstract]) OR ((mhealth[Title/Abstract] OR ehealth[Title/Abstract] OR telemonitoring[Title/Abstract] OR mobile-based[Title/Abstract] OR "mobile based"[Title/Abstract] OR app-based[Title/Abstract] OR "app based"[Title/Abstract] OR computer-based[Title/Abstract] OR "computer based"[Title/Abstract] OR web-based[Title/Abstract] OR "web based"[Title/Abstract] OR internet-based[Title/Abstract] OR "internet based"[Title/Abstract] OR "digital health"[Title/Abstract] OR health[Title/Abstract] OR mobile[Title/Abstract] OR smartphone[Title/Abstract] OR smartwatch[Title/Abstract] OR wearable[Title/Abstract] OR computer[Title/Abstract] OR online[Title/Abstract] OR hybrid[Title/Abstract] OR digital[Title/Abstract] OR behaviour*[Title/Abstract] OR behavior*[Title/Abstract] OR ((behaviour*[Title/Abstract] OR behavior*[Title/Abstract]) AND (change[Title/Abstract] OR modification[Title/Abstract])) OR lifestyle[Title/Abstract]) AND (intervention*[Title/Abstract] OR program*[Title/Abstract] OR coaching[Title/Abstract] OR coach[Title/Abstract] OR telemonitoring[Title/Abstract] OR technology[Title/Abstract] OR app[Title/Abstract] OR apps[Title/Abstract] OR application*[Title/Abstract] OR "health coaching"[Title/Abstract] OR recommendation*[Title/Abstract] OR feedback[Title/Abstract] OR notification*[Title/Abstract] OR message*[Title/Abstract] OR text-message*[Title/Abstract] OR "text message"[Title/Abstract] OR "text messages"[Title/Abstract] OR reminder*[Title/Abstract] OR prompt*[Title/Abstract])) OR (blended-care[Title/Abstract] OR "blended care"[Title/Abstract] OR "blended coaching"[Title/Abstract])) | 1.963.361 |
| Tailoring / personalisation | #3 | (tailored[Title/Abstract] OR tailoring[Title/Abstract] OR personalised[Title/Abstract] OR personalized[Title/Abstract] OR personalisation[Title/Abstract] OR personalization[Title/Abstract] OR personalising[Title/Abstract] OR personalizing[Title/Abstract] OR adaptive[Title/Abstract] OR persuasive[Title/Abstract] OR context-aware[Title/Abstract] OR "context aware"[Title/Abstract] OR "context awareness"[Title/Abstract] OR computer-tailored[Title/Abstract] OR JITAI[Title/Abstract] OR "just-in-time"[Title/Abstract] OR "just in the moment"[Title/Abstract] OR "content matching"[Title/Abstract] OR "content-matching"[Title/Abstract] OR "source matching"[Title/Abstract] OR "user targeting"[Title/Abstract] OR "goal setting"[Title/Abstract] OR "adaptive goal setting"[Title/Abstract]) OR ((mobile[Title/Abstract] OR dynamic[Title/Abstract] OR "context based"[Title/Abstract] OR context-based[Title/Abstract] OR context[Title/Abstract] OR computer-based[Title/Abstract] OR computer[Title/Abstract] OR web-based[Title/Abstract] OR internet-based[Title/Abstract] OR digital[Title/Abstract] OR app-based[Title/Abstract] OR person-specific[Title/Abstract] OR "person specific"[Title/Abstract] OR user-based[Title/Abstract] OR individual-based[Title/Abstract] OR cluster-based[Title/Abstract] OR group-driven[Title/Abstract] OR group-based[Title/Abstract] OR community-based[Title/Abstract] OR community-level[Title/Abstract]) AND (tailoring[Title/Abstract] OR tailored[Title/Abstract] OR personalised[Title/Abstract] OR personalized[Title/Abstract] OR personalisation[Title/Abstract] OR personalization[Title/Abstract] OR personalising[Title/Abstract] OR personalizing[Title/Abstract])) | 438.489 |
| Lifestyle domains | #4 | ("Healthy Lifestyle"[Mesh] OR "Exercise"[Mesh] OR "Sedentary Behavior"[Mesh] OR "Diet, Healthy"[Mesh] OR "Diet, Diabetic"[Mesh] OR "Sleep"[Mesh] OR "Stress, Psychological"[Mesh]) OR (("physical activity"[Title/Abstract] OR exercise[Title/Abstract] OR exercising[Title/Abstract] OR movement[Title/Abstract] OR workout*[Title/Abstract] OR training*[Title/Abstract] OR "active lifestyle"[Title/Abstract] OR ((activity[Title/Abstract] OR sedentary[Title/Abstract]) AND (behaviour[Title/Abstract] OR behavior[Title/Abstract])) OR nutrition[Title/Abstract] OR diet[Title/Abstract] OR (health*[Title/Abstract] AND (eating[Title/Abstract] OR diet[Title/Abstract])) OR "dietary habits"[Title/Abstract] OR "dietary regimen"[Title/Abstract] OR ((vegetable[Title/Abstract] OR fruit[Title/Abstract] OR food[Title/Abstract]) AND (intake[Title/Abstract] OR consumption[Title/Abstract])) OR ((eating[Title/Abstract] OR sleeping[Title/Abstract] OR sleep[Title/Abstract]) AND habits[Title/Abstract]) OR nourishment[Title/Abstract] OR "weight loss"[Title/Abstract] OR sleep*[Title/Abstract] OR bedtime[Title/Abstract] OR stress[Title/Abstract] OR "chronic stress"[Title/Abstract])) | 3.381.712 |
| Cardiometabolic conditions | #5 | (("Overweight"[Mesh]) OR (("metabolic syndrome"[Title/Abstract] OR "metabollic syndrome"[Title/Abstract] OR pre-diabet*[Title/Abstract] OR prediabet*[Title/Abstract] OR ((cardiovascular[Title/Abstract] OR cardiometabolic[Title/Abstract]) AND (disease[Title/Abstract] OR risk*[Title/Abstract])) OR CVD[Title/Abstract] OR diabet*[Title/Abstract] OR T2D[Title/Abstract] OR T2DM[Title/Abstract] OR hyperglycemia[Title/Abstract] OR hyperglycaemia[Title/Abstract] OR (high[Title/Abstract] AND ("glycemic index"[Title/Abstract] OR "glycaemic index"[Title/Abstract])) OR "impaired glucose metabolism"[Title/Abstract] OR "glucose metabolism disorder"[Title/Abstract] OR dysglycemia[Title/Abstract] OR "insulin resistance"[Title/Abstract] OR obes*[Title/Abstract] OR overweight[Title/Abstract] OR dyslipidemia[Title/Abstract] OR triglycerides[Title/Abstract] OR ((high*[Title/Abstract] OR increased[Title/Abstract] OR elevated[Title/Abstract]) AND ("body weight"[Title/Abstract] OR "body mass index"[Title/Abstract] OR BMI[Title/Abstract] OR "glycated hemoglobin"[Title/Abstract] OR "blood pressure"[Title/Abstract] OR cholesterol[Title/Abstract] OR "blood glucose"[Title/Abstract])) OR hypertension[Title/Abstract]))) NOT (("gestational diabetes"[Title/Abstract] OR pregnant[Title/Abstract] OR pregnancy[Title/Abstract] OR "mental disorder"[Title/Abstract] OR "mental illness"[Title/Abstract])) | 2.141.179 |
| Resulting query | #6 | #1 AND #2 AND #3 AND #4 AND #5 | 774 |
| **Resulting query with added filters** | **#7** | **#1 AND #2 AND #3 AND #4 AND #5 AND ((english[Filter]) AND (2000:2024[pdat]))** | **759** |
| **Updated search** |  |  | **923** |

## PsycINFO (EBSCO)

- Date of search: 03-05-2024
- Searching within: TI (Title) OR AB (Abstract) OR KW (Keywords) for each cluster
- Keywords: See Table below
  - *Search modes – Boolean/Phrase*
- Booleans between the search clusters: AND
- Search filters:
  - Range of years: 2000 - 2024
  - Language: English
  - Exclusion filters: Exclude Dissertations

| **PsycINFO (EBSCO)** | | | |
| --- | --- | --- | --- |
| **Key constructs** | **Cluster #** | **Keywords** | **Results** |
| Low socioeconomic position | #S1 | **TI** ( ((low OR lower) NEAR/5 (socio-economic OR “socio economic” OR socioeconomic)) OR ((socio-economic OR “socio economic” OR socioeconomic) NEAR/5 (position OR status OR disparity OR inequality)) OR “SES” OR “SEP” OR “social determinants of health” OR (health AND (disparity OR inequality OR discrepancy)) OR ((deprived OR disadvantaged OR vulnerable OR impoverished OR underprivileged) NEAR/5 (neighbourhood* OR neighborhood*)) OR rural OR (low-income OR “low income” OR limited-income OR “limited income” OR “lower-middle income”) OR “low-educated” OR ((low OR lower) NEAR/5 (“economic status” OR educated OR education OR “education level” OR literacy OR “health literacy” OR “digital literacy” OR “digital health literacy” OR numeracy OR “computer literacy” OR “media literacy” OR “science literacy” OR “information literacy” OR “eHealth literacy”)) )  **OR AB** ( ((low OR lower) NEAR/5 (socio-economic OR “socio economic” OR socioeconomic)) OR ((socio-economic OR “socio economic” OR socioeconomic) NEAR/5 (position OR status OR disparity OR inequality)) OR “SES” OR “SEP” OR “social determinants of health” OR (health AND (disparity OR inequality OR discrepancy)) OR ((deprived OR disadvantaged OR vulnerable OR impoverished OR underprivileged) NEAR/5 (neighbourhood* OR neighborhood*)) OR rural OR (low-income OR “low income” OR limited-income OR “limited income” OR “lower-middle income”) OR “low-educated” OR ((low OR lower) NEAR/5 (“economic status” OR educated OR education OR “education level” OR literacy OR “health literacy” OR “digital literacy” OR “digital health literacy” OR numeracy OR “computer literacy” OR “media literacy” OR “science literacy” OR “information literacy” OR “eHealth literacy”)) )  **OR KW** ( ((low OR lower) NEAR/5 (socio-economic OR “socio economic” OR socioeconomic)) OR ((socio-economic OR “socio economic” OR socioeconomic) NEAR/5 (position OR status OR disparity OR inequality)) OR “SES” OR “SEP” OR “social determinants of health” OR (health AND (disparity OR inequality OR discrepancy)) OR ((deprived OR disadvantaged OR vulnerable OR impoverished OR underprivileged) NEAR/5 (neighbourhood* OR neighborhood*)) OR rural OR (low-income OR “low income” OR limited-income OR “limited income” OR “lower-middle income”) OR “low-educated” OR ((low OR lower) NEAR/5 (“economic status” OR educated OR education OR “education level” OR literacy OR “health literacy” OR “digital literacy” OR “digital health literacy” OR numeracy OR “computer literacy” OR “media literacy” OR “science literacy” OR “information literacy” OR “eHealth literacy”)) )  **NOT** TI ( (child* OR adolescent*) ) NOT AB ( (child* OR adolescent*) ) NOT KW ( (child* OR adolescent*) ) | 130.269 |
| eHealth lifestyle intervention | #S2 | **TI** ( (mHealth OR ehealth OR e-health OR m-health OR “mobile health” OR “electronic health” OR e-coaching OR eCoaching OR telemedicine OR “personal health informatics” OR “health promotion” OR “recommender system” OR “recommendation system” OR “recommender systems” OR “recommendation systems”) OR ((mhealth OR ehealth OR telemonitoring OR mobile-based OR “mobile based” OR app-based OR “app based” OR computer-based OR “computer based” OR web-based OR “web based” OR internet-based OR “internet based” OR “digital health” OR health OR mobile OR smartphone OR smartwatch OR wearable OR computer OR online OR hybrid OR digital OR behaviour* OR behavior* OR ((behaviour* OR behavior*) NEAR/5 (change OR modification)) OR lifestyle) NEAR/25 (intervention* OR program* OR coaching OR coach OR telemonitoring OR technology OR app OR apps OR application* OR “health coaching” OR recommendation* OR feedback OR notification* OR message* OR text-message* OR “text message” OR “text messages” OR reminder* OR prompt*)) OR (blended-care OR “blended care” OR “blended coaching”) )  **OR AB** ( (mHealth OR ehealth OR e-health OR m-health OR “mobile health” OR “electronic health” OR e-coaching OR eCoaching OR telemedicine OR “personal health informatics” OR “health promotion” OR “recommender system” OR “recommendation system” OR “recommender systems” OR “recommendation systems”) OR ((mhealth OR ehealth OR telemonitoring OR mobile-based OR “mobile based” OR app-based OR “app based” OR computer-based OR “computer based” OR web-based OR “web based” OR internet-based OR “internet based” OR “digital health” OR health OR mobile OR smartphone OR smartwatch OR wearable OR computer OR online OR hybrid OR digital OR behaviour* OR behavior* OR ((behaviour* OR behavior*) NEAR/5 (change OR modification)) OR lifestyle) NEAR/25 (intervention* OR program* OR coaching OR coach OR telemonitoring OR technology OR app OR apps OR application* OR “health coaching” OR recommendation* OR feedback OR notification* OR message* OR text-message* OR “text message” OR “text messages” OR reminder* OR prompt*)) OR (blended-care OR “blended care” OR “blended coaching”) )  **OR KW** ( (mHealth OR ehealth OR e-health OR m-health OR “mobile health” OR “electronic health” OR e-coaching OR eCoaching OR telemedicine OR “personal health informatics” OR “health promotion” OR “recommender system” OR “recommendation system” OR “recommender systems” OR “recommendation systems”) OR ((mhealth OR ehealth OR telemonitoring OR mobile-based OR “mobile based” OR app-based OR “app based” OR computer-based OR “computer based” OR web-based OR “web based” OR internet-based OR “internet based” OR “digital health” OR health OR mobile OR smartphone OR smartwatch OR wearable OR computer OR online OR hybrid OR digital OR behaviour* OR behavior* OR ((behaviour* OR behavior*) NEAR/5 (change OR modification)) OR lifestyle) NEAR/25 (intervention* OR program* OR coaching OR coach OR telemonitoring OR technology OR app OR apps OR application* OR “health coaching” OR recommendation* OR feedback OR notification* OR message* OR text-message* OR “text message” OR “text messages” OR reminder* OR prompt*)) OR (blended-care OR “blended care” OR “blended coaching”) ) | 37.553 |
| Tailoring / personalisation | #S3 | **TI** ( (tailored OR tailoring OR personalised OR personalized OR personalisation OR personalization OR personalising OR personalizing OR adaptive OR persuasive OR context-aware OR “context aware” OR “context awareness” OR computer-tailored OR JITAI OR “just-in-time” OR “just in the moment” OR “content matching” OR “content-matching” OR “source matching” OR “user targeting” OR “goal setting” OR “adaptive goal setting”) OR ((mobile OR dynamic OR “context based” OR context-based OR context OR computer-based OR computer OR web-based OR internet-based OR digital OR app-based OR person-specific OR “person specific” OR user-based OR individual-based OR cluster-based OR group-driven OR group-based OR community-based OR community-level) NEAR/25 (tailoring OR tailored OR personalised OR personalized OR personalisation OR personalization OR personalising OR personalizing)) )  **OR AB** ( (tailored OR tailoring OR personalised OR personalized OR personalisation OR personalization OR personalising OR personalizing OR adaptive OR persuasive OR context-aware OR “context aware” OR “context awareness” OR computer-tailored OR JITAI OR “just-in-time” OR “just in the moment” OR “content matching” OR “content-matching” OR “source matching” OR “user targeting” OR “goal setting” OR “adaptive goal setting”) OR ((mobile OR dynamic OR “context based” OR context-based OR context OR computer-based OR computer OR web-based OR internet-based OR digital OR app-based OR person-specific OR “person specific” OR user-based OR individual-based OR cluster-based OR group-driven OR group-based OR community-based OR community-level) NEAR/25 (tailoring OR tailored OR personalised OR personalized OR personalisation OR personalization OR personalising OR personalizing)) )  **OR KW** ( (tailored OR tailoring OR personalised OR personalized OR personalisation OR personalization OR personalising OR personalizing OR adaptive OR persuasive OR context-aware OR “context aware” OR “context awareness” OR computer-tailored OR JITAI OR “just-in-time” OR “just in the moment” OR “content matching” OR “content-matching” OR “source matching” OR “user targeting” OR “goal setting” OR “adaptive goal setting”) OR ((mobile OR dynamic OR “context based” OR context-based OR context OR computer-based OR computer OR web-based OR internet-based OR digital OR app-based OR person-specific OR “person specific” OR user-based OR individual-based OR cluster-based OR group-driven OR group-based OR community-based OR community-level) NEAR/25 (tailoring OR tailored OR personalised OR personalized OR personalisation OR personalization OR personalising OR personalizing)) ) | 119.125 |
| Lifestyle domains | #S4 | **TI** ( (“physical activity” OR exercise OR exercising OR movement OR workout* OR training* OR “active lifestyle” OR ((activity OR sedentary) NEAR/5 (behaviour OR behavior)) OR nutrition OR diet OR (health* NEAR/5 (eating OR diet)) OR “dietary habits” OR “dietary regimen” OR ((vegetable OR fruit OR food) NEAR/5 (intake OR consumption)) OR ((eating OR sleeping OR sleep) NEAR/5 habits) OR nourishment OR “weight loss” OR sleep* OR bedtime OR stress OR “chronic stress”) )  **OR AB** ( (“physical activity” OR exercise OR exercising OR movement OR workout* OR training* OR “active lifestyle” OR ((activity OR sedentary) NEAR/5 (behaviour OR behavior)) OR nutrition OR diet OR (health* NEAR/5 (eating OR diet)) OR “dietary habits” OR “dietary regimen” OR ((vegetable OR fruit OR food) NEAR/5 (intake OR consumption)) OR ((eating OR sleeping OR sleep) NEAR/5 habits) OR nourishment OR “weight loss” OR sleep* OR bedtime OR stress OR “chronic stress”) )  **OR KW** ( (“physical activity” OR exercise OR exercising OR movement OR workout* OR training* OR “active lifestyle” OR ((activity OR sedentary) NEAR/5 (behaviour OR behavior)) OR nutrition OR diet OR (health* NEAR/5 (eating OR diet)) OR “dietary habits” OR “dietary regimen” OR ((vegetable OR fruit OR food) NEAR/5 (intake OR consumption)) OR ((eating OR sleeping OR sleep) NEAR/5 habits) OR nourishment OR “weight loss” OR sleep* OR bedtime OR stress OR “chronic stress”) ) | 861.699 |
| Cardiometabolic conditions | #S5 | **TI** ( (“metabolic syndrome” OR “metabollic syndrome” OR pre-diabet* OR prediabet* OR ((cardiovascular OR cardiometabolic) NEAR/5 (disease OR risk*)) OR CVD OR diabet* OR T2D OR T2DM OR hyperglycemia OR hyperglycaemia OR (high NEAR/5 (“glycemic index” OR “glycaemic index”)) OR “impaired glucose metabolism” OR “glucose metabolism disorder” OR dysglycemia OR “insulin resistance” OR obes* OR overweight OR dyslipidemia OR triglycerides OR ((high* OR increased OR elevated) NEAR/5 (“body weight” OR “body mass index” OR BMI OR “glycated hemoglobin” OR “blood pressure” OR cholesterol OR “blood glucose”)) OR hypertension) )  **OR AB** ( (“metabolic syndrome” OR “metabollic syndrome” OR pre-diabet* OR prediabet* OR ((cardiovascular OR cardiometabolic) NEAR/5 (disease OR risk*)) OR CVD OR diabet* OR T2D OR T2DM OR hyperglycemia OR hyperglycaemia OR (high NEAR/5 (“glycemic index” OR “glycaemic index”)) OR “impaired glucose metabolism” OR “glucose metabolism disorder” OR dysglycemia OR “insulin resistance” OR obes* OR overweight OR dyslipidemia OR triglycerides OR ((high* OR increased OR elevated) NEAR/5 (“body weight” OR “body mass index” OR BMI OR “glycated hemoglobin” OR “blood pressure” OR cholesterol OR “blood glucose”)) OR hypertension) )  **OR KW** ( (“metabolic syndrome” OR “metabollic syndrome” OR pre-diabet* OR prediabet* OR ((cardiovascular OR cardiometabolic) NEAR/5 (disease OR risk*)) OR CVD OR diabet* OR T2D OR T2DM OR hyperglycemia OR hyperglycaemia OR (high NEAR/5 (“glycemic index” OR “glycaemic index”)) OR “impaired glucose metabolism” OR “glucose metabolism disorder” OR dysglycemia OR “insulin resistance” OR obes* OR overweight OR dyslipidemia OR triglycerides OR ((high* OR increased OR elevated) NEAR/5 (“body weight” OR “body mass index” OR BMI OR “glycated hemoglobin” OR “blood pressure” OR cholesterol OR “blood glucose”)) OR hypertension) )  **NOT TI** ( (“gestational diabetes” OR pregnant OR pregnancy OR “mental disorder” OR “mental illness”) ) NOT AB ( (“gestational diabetes” OR pregnant OR pregnancy OR “mental disorder” OR “mental illness”) ) NOT KW ( (“gestational diabetes” OR pregnant OR pregnancy OR “mental disorder” OR “mental illness”) ) | 101.910 |
| Resulting query | #S6 | #S1 AND #S2 AND #S3 AND #S4 AND #S5 | 41 |
| **Resulting query with added filters** | **#S7** | **#S1 AND #S2 AND #S3 AND #S4 AND #S5**  **Limiters - Publication Year: 2000-2024; English language; Exclude Dissertations** | **35** |
| **Update search** |  |  | **36** |

1. <https://researchguides.uic.edu/searchstrategies/proximityoperators#:~:text=Typically%2C%20the%20operators%20are%20composed,number%2C%20the%20narrower%20the%20search>.
