# Supplementary material for: Tailoring in eHealth lifestyle interventions targeting people with cardiometabolic conditions and lower socioeconomic position: A scoping review
Source: PLOS Digit Health. 2026 Sep 11;5(9):e0001713. doi: 10.1371/journal.pdig.0001713 (PMC13568521; doi:10.1371/journal.pdig.0001713)
Supplement: Supplementary file 3 [file pdig.0001713.s003.docx]

**S2 Table. Tailoring areas, levels and variables with facilitating tailoring functions, per intervention.**

| **Id*** | **Tailoring area** | **Algorithm function** | **Variables** | **Granularity of tailoring variables** | **Variable collection rate** | **Tailoring rate** | **Granularity of output** | **Static /dynamic tailoring** |
| --- | --- | --- | --- | --- | --- | --- | --- | --- |
| I-1 | Content | Variable substitution: inserting participant-relevant values into messages | Participant's name (person-specific), preference on weight units (grouped) | Both | Baseline | When messages are sent (When progress messages are sent at 3,6,12 months) | Specific to an individual | Static |
| I-1 | Content | Feedback selection & progress calculation: feedback on goal progression (weight change) | Weight values, incentive targets (system-derived) | Variables are specific to the individual | Periodically  (Appointments at 3,6, 12 months) | Real-time (When data is available) | Relevant to a group | Dynamic |
| I-1 | Representation (visuals) | Progress calculation & visualization | Weight data, incentive data | Variables are specific to the individual | Periodically  (Appointments at 3,6, 12 months) | Real-time (When data is available) | Specific to an individual | Dynamic |
| I-2 | Content | Feedback selection, variable substitution & progress calculation | Weight, goal adherence (grouped) | Both | Frequently  (Once a week) | Real-time (Immediately, to user response) | Relevant to a group | Dynamic |
| I-2 | Goal-setting | Recommendation provision: goal level increase suggestion | Successful goal adherence (reached goal or not) | Variables belong to segments | Frequently  (Once a week) | Real-time (When goal is achieved) | Relevant to a group | Dynamic |
| I-3 | Content | Feedback selection & progress calculation | Weight, weight change, user creating plans for reaching goals | Both | Frequently  (Twice a week) | Real-time (Immediately, to user response) | Relevant to a group | Dynamic |
| I-3 | Content | Content filtering: selection of intervention content type (reminders, tips, education…) | Selected goal, day of the week | Variables belong to segments | Frequently  (Once a week) | Frequent schedule (Weekly (not immediately visible to the user)) | Relevant to a group | Static |
| I-3 | Timing | Content filtering: Selection of content for users who request additional tips/information on dealing with cravings | User request for additional information on cravings | Variables belong to segments | Event-based  (At user’s request) | Real-time (Immediately, at user's request) | Relevant to a group | Dynamic |
| I-4 | Content | Variable substitution: inserting goal-relevant information into self-monitoring messages | Goal data (chosen goal out of a set) | Variables belong to segments | Periodically  (Every 6 months) | Periodic schedule (When the user answers the survey, but not immediately visible - tailoring is updated after six months) | Relevant to a group | Static |
| I-4 | Content | Feedback selection, progress calculation & variable substitution: feedback type/response selection with progress trends, success reinforcement, motivational strategies, short skills training | Goal progress (numerical values, e.g., 1-5), goal data, user’s response to the IVR | Variables belong to segments | Frequently  (Once a week) | Real-time (Immediately, to user response) | Specific to an individual | Dynamic |
| I-4 | Intervention functionality | Structured adaptive logic & progress calculation: deciding the frequency of in-person counselling | Weekly average weight change - but the weight is assigned into a zone | Both | Frequently (Daily, or at least once a week) | Frequent schedule (Once a week) | Relevant to a group | Dynamic |
| I-4 | Goal-setting | Content prioritization (of goals): categorizing the user's current behaviors and tailoring the list of goals | Current behaviors of the participant | Variables belong to segments | Periodically  (Every 6 months) | Real-time (Immediately, to user response) | Relevant to a group | Static |
| I-5 | Content | Feedback selection: feedback on data, with describing trends | Goal adherence | Variables belong to segments | Frequently (Once a week from daily offline records) | Real-time (Immediately, to user response) | Relevant to a group | Dynamic |
| I-6 | Content | Geofencing:  Content of the messages sent to the user of available PA classes within their geofence | PA locations & classes available in user's geofence | Variables belong to segments | Frequently  (Daily) | Frequent schedule (Daily) | Relevant to a group | Dynamic |
| I-6 | Content | Variable substitution & recommendation provision:  Sending a reminder message with information on the class the user selected the day before | User preferences on the selected PA type, meta data of that class | Variables belong to segments | Frequently  (Daily) | Frequent schedule (Daily) | Relevant to a group | Dynamic |
| I-7 | Goal-setting | Content prioritization (of goals): ranking of the goals suggested to the user | Behavior change variables for goal ranking | Variables belong to segments | Periodically (Every 6 months) | Real-time (When the user answers the survey) | Relevant to a group | Dynamic |
| I-7 | Content | Feedback selection, progress calculation & variable substitution: selecting type of feedback message and describing trends | Responses on goal adherence, selected goals | Variables belong to segments | Frequently (Once a week) | Real-time (Immediately, to user response) | Specific to an individual | Dynamic |
| I-7 | Content | Feedback selection & progress calculation: weight status feedback | Average weekly weight changes | Variables are specific to the individual | Frequently (Daily, or at least once a week) | Frequent schedule (Once a week) | Relevant to a group | Dynamic |
| I-8 | Goal-setting | Structured adaptive logic & recommendation provision: The user goes through interactive assessment tool | Dietary intake, PA level, socio-cognitive beliefs selected goals and action, preparatory, and coping plans, goal-adherence, demographic characteristics | Both | Event-based  (User decides when they enter their data/responses) | Real-time (Immediately, to user response) | Relevant to a group | Dynamic |
| I-8 | Content | Recommendation provision: receiving optional advice on how to deal with user-indicated difficult situations | Selected difficult situations (from a list) | Variables belong to segments | Event-based (Data is collected when the user requests feedback) | Real-time (Immediately, to user response) | Relevant to a group | Dynamic |
| I-8 | Content | Recommendation provision: providing advice on overcoming difficult situations in reaching goals; an option the user can decide to choose | Selected difficult situations (from a list) | Variables belong to segments | Periodically (On a monthly basis) | Real-time (Immediately, to user response) | Relevant to a group | Dynamic |
| I-8 | Content | Feedback selection: selecting feedback based on user's assessment responses | Body weight, psychological variables, adherence to health behaviors, | Both | Periodically (On a monthly basis) | Real-time (Immediately, to user response) | Relevant to a group | Dynamic |
| I-8 | Content | Feedback selection & progress calculation: selecting feedback on goal progression | Body weight, weight change | Variables are specific to the individual | Event-based (User decides when they enter their data/responses) | Real-time (Immediately, to user response) | Relevant to a group | Dynamic |
| I-8 | Representation (visuals) | Progress visualization | Health data | Variables are specific to the individual | Event-based (When the user is interacting with the session) | Real-time (Immediately, to user response) | Specific to an individual | Dynamic |
| I-9 | Timing & Frequency | Content filtering: filtering when to send certain messages | Individual user's patterns on location, social context | Variables are specific to the individual | Continuously (GPS data) | Real-time (Immediately, to user location) | Specific to an individual | Dynamic |
| I-9 | Content | Content prioritization: adapting the prioritization of the messages according to user likes/dislikes | User's given like/dislike on a message | Variables belong to segments | Event-based (When user reacts to message) | Real-time (In the background, to user response (not immediately clear to user)) | Specific to an individual | Dynamic |
| I-10 | Content + timing | Content filtering, progress calculation, feedback selection & variable substitution: selection of a message category at the right time that increases PA likelihood for the individual | Fixed variables: profiling, health profile, psychological, socio-behavioral, health behaviors; dynamic: historical message data, historical step data, meta-data | Both | Baseline + Continuously  (Continuously for e.g., step data) | Frequent schedule (2x a day (two messages sent per day)) | Specific to an individual | Dynamic |
| I-11 | Content | Feedback selection based on numerical input from the user | Numerical input on behavior adherence (0-7) | Variables belong to segments | Once a week Frequently | Real-time (Immediately, to user response) | Relevant to a group | Dynamic |
| I-11 | Intervention functionality | Structured adaptive logic: deciding when to initiate contact with care manager/health educator | Out of range numerical input on behavior adherence | Variables belong to segments | Frequently (Once a week) | Real-time (When a rule is satisfied (out of range value)) | Relevant to a group | Dynamic |
| I-12 | Content | Structured adaptive logic: selecting whether the video is offered as optional or required | Answer to quiz question (choice-based) | Variables belong to segments | Event-based (When user views a video) | Real-time (Immediately, to user response) | Relevant to a group | Dynamic |
| I-13 | Content | Recommendation provision: based on the user's health profile | User's health profile data (health condition, physiological profile, social profile, PA level, exercise preferences…) | Both | Baseline | At baseline | Specific to an individual | Static |
| I-13 | Content + Timing | Recommendation provision & variable substitution: to the user's current context, profile, preferences and behaviors | User's current (dynamic) context (nutritional values of items on the menu), health profile, recommended daily nutritional intake values | Variables are specific to the individual | Event-based (At user's request) | Real-time (Immediately, to user request) | Specific to an individual | Dynamic |
| I-13 | Content | Recommendation provision & variable substitution: on goal compensation | Dynamic context information - what the user ate during the day, exercise preferences, weather, etc. | Both | Frequently (Throughout the day) | Frequent schedule (At the end of the day) | Specific to an individual | Dynamic |
| I-13 | Content | Feedback selection & variable substitution: daily summary on nutritional intake - sum of values and comparison to the recommended amount (i.e. Goal) | User's recommended nutritional intake; recorded food intake | Variables are specific to the individual | Frequently (Throughout the day) | Frequent schedule (At the end of the day) | Specific to an individual | Dynamic |
| I-14 | Content | Feedback selection based on numerical input | Goal adherence (numerical inputs (e.g., 1-5) | Variables belong to segments | Frequently (Once a week) | Real-time (Immediately, to user response) | Relevant to a group | Dynamic |
| I-14 | Goal-setting | Recommendation provision: goal level increase suggestion | Successful goal adherence (reached goal or not) | Variables belong to segments | Frequently (Once a week) | Real-time (Immediately, to user response) | Relevant to a group | Dynamic |
| I-15 | Content | Content filtering: type of messages to be sent every 3 months | Behavioral survey responses, preferences | Variables belong to segments | Periodically (Every three months) | Periodic schedule (For the next 3 months, following a survey (not immediately visible to the user)) | Specific to an individual | Static |
| I-15 | Content | Variable substitution: inserting participant-relevant values into messages | Personal characteristics, e.g., Name | Variables are specific to the individual | Baseline | When messages are sent | Specific to an individual | Static |
| I-15 | Timing & Frequency | Structured adaptive logic: frequency of messages are sent | User preference on message frequency | Variables belong to segments | Periodically (Every three months) | Periodic schedule (For the next 3 months, following a survey (not immediately visible to the user)) | Relevant to a group | Static |
| I-15 | Intervention functionality | Content filtering: the proportion of the messages that were selected as matching the user's preferences on the message topic (50, 60 or 70%) | Evaluation (improvement/ regression) of self-reported self-care behaviors (in diet, exercise, medication use) | Variables belong to segments | Periodically (Once a month) | Periodic schedule (Monthly, not immediately visible to the user) | Relevant to a group | Static |
| I-16 | Content | Feedback selection: feedback on goal adherence | Adherence logs | Variables are specific to the individual | Frequently (Once a week) | Real-time (Immediately, to user response) | Relevant to a group | Dynamic |
| I-16 | Content | Content filtering & variable substitution: content of the message sent (also for a self-monitoring request) relate to the goal | Goals chosen during coaching sessions | Variables belong to segments | Periodically (Once a month) | Periodic schedule (Once a month - visible in daily messages to the user) | Relevant to a group | Static |
| I-16 | Content | Variable substitution: participant-relevant information in messages sent to the support person | Personal characteristics (e.g., Name) | Variables are specific to the individual | Baseline | When messages are sent | Specific to an individual | Static |
| I-16 | Timing & Frequency | Structured adaptive logic: timing of message delivery | Preferences | - | Baseline | - | - | - |
| I-17 | Content | Feedback selection & progress calculation: type of message + describing trends | Data logs, questionnaire data on fruit/vegetable intake & fat consumption | Variables are specific to the individual | Frequently (Daily, or at least once a week) | Real-time (Immediately when data is entered) | Specific to an individual | Dynamic |
| I-17 | Timing & Frequency | Structured adaptive logic: reminder delivery on logging adherence | Recorded missing values of adherence logs | Variables belong to segments | Frequently (Daily) | Real-time (When 6 days of missing values are recorded) | Relevant to a group | Dynamic |
| I-17 | Content | Structured adaptive logic: system response on various topics to help with action planning + comparison to recommended values | Preferences, psychological and profiling variables | Variables belong to segments | Periodically (On a basis of 6 weeks (optionally)) | Real-time (Immediately, to user response) | Relevant to a group | Dynamic |
| I-17 | Representation (visuals) | Progress visualization | Data logs (laboratory results & self-monitoring) | Variables are specific to the individual | Frequently (Daily, or at least once a week (unknown for laboratory results) | Real-time (Immediately when data is entered) | Specific to an individual | Dynamic |
| I-18 | Content, Representation (visuals) | Feedback selection & variable substitution: provides a descriptive report of the recorded values of the previous week together with stoplight-based selection of emoticons | Stored data logs (glucose measurements) from the week | Variables are specific to the individual | Frequently (Daily) | Frequent schedule (Weekly) | Specific to an individual | Dynamic |
| I-18 | Content | Feedback selection: feedback on currently provided glucose values | Current data logs (blood glucose) | Variables are specific to the individual | Frequently (Daily) | Real-time (Immediately, to user response) | Relevant to a group | Dynamic |
| I-19 | Content | Variable substitution: inserting participant-relevant values into messages | Participant name | Variables are specific to the individual | Baseline | When messages are sent | Specific to an individual | Static |
| I-19 | Timing & Frequency | Structured adaptive logic: timing of message delivery | Preferences | - | Baseline | - | - | - |
| I-20 | Content & Goal-setting | Feedback selection & content filtering: feedback to quiz answers; or how to increase PA | Current PA levels, psychological variables, quiz responses | Variables belong to segments | Event-based (During user interaction) | Real-time (Immediately, to user response) | Relevant to a group | Dynamic |
| I-20 | Representation (visuals) | Content filtering: selection of intervention content according to the participant's age, gender and culture | Personal characteristics (age, gender, culture) | Variables are specific to the individual | Baseline | Baseline | Relevant to a group | Static |
| I-21 | Content | Content filtering: messages to the user are selected based on several contextual & personal variables | Statically collected variables (e.g., Mobility, self-efficacy, community) and user's current context (e.g., Time of the day, weather) | Variables belong to segments | Baseline + Frequently (Daily) | Frequent schedule (Daily / once a day, adapted before the message is sent out) | Relevant to a group | Dynamic |
| I-21 | Content | Variable substitution: inserting participant-relevant values into messages | Participant name | Variables are specific to the individual | Baseline | When messages are sent | Specific to an individual | Static |
| I-21 | Timing & Frequency | Content filtering: Timing of message delivery | User preference on message timing (4 time-points in the day) not included in the table | Variables belong to segments | Baseline | At baseline | Relevant to a group | Static |
| I-21 | Representation (visuals) | Progress visualization | Fitbit data, low sodium food choices | Variables are specific to the individual | Continuously + Frequently (Daily) | Real-time (When data is entered) | Specific to an individual | Dynamic |
| I-22 | Content | Content filtering: selection of messages within a newsletter | Behavioral survey responses | Variables belong to segments | Periodically (Every three months) | Periodic schedule (Every three months, not immediately visible to the user) | Relevant to a group | Static |
| I-22 | Representation (visuals) | Progress visualization | Data logs on blood pressure, eating and activity | Variables are specific to the individual | Frequently (At most daily) | Real-time (Immediately, when data is entered) | Specific to an individual | Dynamic |
| I-23 | Content | Structured adaptive logic & variable substitution: changes in the text according to the user's choices/responses | User responses within the action-planning tool (choice-based) | Variables belong to segments | Event-based (During user interaction) | Real-time (Immediately, to user response) | Relevant to a group | Static |
| I-24 | Content | Content filtering & variable substitution: daily messages relating to steps or other data | Fitbit data (sleep, steps, heart rate…) | Variables are specific to the individual | Continuously via Fitbit | Frequent schedule (Daily) | Specific to an individual | Dynamic |
| I-24 | Content + timing | Geofencing: sending the right message at the right time - when the user approaches a certain location | User's geofence, user preferences on PA locations | Variables are specific to the individual | Continuously (GPS data) | Real-time (Immediately, when user enters the geofence) | Specific to an individual | Dynamic |
| I-24 | Goal-setting | Adaptive goal-setting: adaptively change the step goal according to previous week's average | Weekly steps average | Variables are specific to the individual | Continuously (Step data) | Frequent schedule (Weekly) | Specific to an individual | Dynamic |
| I-24 | Representation (visuals) | Progress visualization | Automatically collected Fitbit, weight and BP data | Variables are specific to the individual | Continuously + Frequently (At most daily) | Real-time (When data is available) | Specific to an individual | Dynamic |
| I-25 | Content | Feedback selection & progress calculation: feedback type when user enters their log data. Includes comparison to their previous entry | Step data logs (goal-progression - system derived) | Variables are specific to the individual | Frequently (At most daily) | Real-time (Immediately, when data is entered) | Specific to an individual | Dynamic |
| I-25 | Representation (visuals) | Progress visualization | Step data logs | Variables are specific to the individual | Frequently (At most daily) | Real-time (Immediately when data is entered) | Specific to an individual | Dynamic |
| I-26 | Content | Content filtering: selecting the bank of messages related to target behavior and stage of BC | Target behavior, current stage of change (determined during coaching sessions, not changed in-between) | Variables belong to segments | Periodically (Monthly) | Periodic schedule (Monthly) | Relevant to a group | Static |
| I-27 | Goal-setting | Recommendation provision: PA goal recommendation with suggestions on community resources | PA preference (indoor/outdoor), location, health profile & behaviors | Variables belong to segments | Baseline | At baseline | Relevant to a group | Static |
| I-27 | Content | Feedback selection & progress calculation: selection of feedback to user responses | Goal adherence | Variables belong to segments | Frequently (Once a week) | Real-time (Immediately, to user response) | Relevant to a group | Dynamic |
| I-27 | Content | Variable substitution: inserting participant-relevant values into messages | Goal / PA type, name | Both | Baseline | When messages are sent | Specific to an individual | Static |

PA, physical activity; BMI, body mass index; IVR, interactive voice response; BP, blood pressure; EMA, ecological momentary assessment.

*Intervention ID
