# Supplementary material for: Tailoring in eHealth lifestyle interventions targeting people with cardiometabolic conditions and lower socioeconomic position: A scoping review
Source: PLOS Digit Health. 2026 Sep 11;5(9):e0001713. doi: 10.1371/journal.pdig.0001713 (PMC13568521; doi:10.1371/journal.pdig.0001713)
Supplement: Supplementary file 4 [file pdig.0001713.s004.docx]

Categorization to evaluation phases and engagement metrics

The three evaluation phases according to Jansen-Kosterink et al. [1]:

- Research stage: TRL1-TRL3
- Development stage: TRL4-TRL6
- Deployment stage: TRL7-TRL9

**S3 Table. Categorization of each evaluation study into the evaluation phases of research, development, and deployment and the used engagement metrics.**

| ID* | Included papers | TRL category | TRL categorization reasoning | Engagement metrics and results |
| --- | --- | --- | --- | --- |
| I-1 | Dombrowski et al. 2020 [2] | Development | **Study design:** A three-arm individually randomized parallel-group controlled feasibility trial  **Participants (N):** 105  **Stage of technology development**:** Post-prototype  **Setting:** Relevant environment (Daily clinical context)  **Outcomes:** Acceptability, feasibility, assessment of health benefits **Cost analysis:** Not conducted | **Metrics:** Engagement with website & responses to texts  **Results**:   - infrequent text responses & decreasing engagement due to losing interest in the content - some participants would like more interactivity with the texts - most did not access the website |
|  | Macaulay et al. 2022 [3] | - | Protocol | - |
| I-2 | Stein et al. 2019 [4] | - | Protocol | - |
| I-3 | Kharmats et al. 2022 [5] | Development | **Study design:** RCT  **Participants (N):** 155  **Stage of technology development:** Post-prototype stage  **Setting:** Relevant environment  **Outcomes:** Health outcomes, self-reported behavioral changes and psychological variables (e.g., self-efficacy), engagement **Cost analysis:** Not conducted  **Comment:** There were still usability issues with the aggregator and feedback delivery during the study (Jansen-Kosterink et al. [1] mention that in the deployment stage, there are no more "critical usability issues left") | **Metrics:** Response* rates to SMS texts with questions & whether all the provided messages (in SMS or print) were read by the participants  *Participants were told that responding was optional  **Results:**  When asked whether they read all messages:   - SMS group:   - 67.7% of participants strongly agreed   - 16.9% agreed - Printed messages group:   - 22.4% of participants strongly agreed   - 28.4% agreed - The difference between the two groups was statistically significant (p<0.001) - The majority (63% on average) of participants responded to texts asking them for goal selection, with responses declining from 80% to 52% between week 1 and week 16 - On average, 57% responded on questions regarding making plans for reaching their set goal. This response ratio also declined from 76% to 46% between weeks 1 and 16. - On average, 55% sent texts to receive tips for addressing cravings or to report their weight - Overall average of response rate to SMS messages: 51% (moderate). This might have been influenced by the fact that participants were told that responding to the messages is optional. |
| I-4 | Berger et al. 2019 [6] | - | Protocol | - |
| I-5 | Foley et al. 2012 [7] | - | Protocol | - |
|  | Bennett et al. 2013 [8] | Development | **Study design:** RCT  **Participants (N):** 194  **Stage of technology development:** Post-prototype  **Setting:** Relevant environment  **Outcomes:** Health outcomes, engagement **Cost analysis:** Not conducted  **Comment:** research-controlled conditions: "Study staff provided Shape coaches with a 2-day baseline training session, weekly supervision, and refresher trainings every 6 months. Study staff reviewed 5% of coaching calls for protocol adherence." [8] | **Metrics:** Engagement with intervention components, such as responses to IVR calls, counselling calls, or making use of the provided fitness membership  **Results:**   - On average, the authors report an 84.3% completion rate of the counseling calls during 12 months, excluding some participants (5.5%) that asked to stop with counseling calls but continued to engage with other intervention components - The completion rate of the IVR calls varied throughout the week, spanning from 65.2% to 89.5%, and averaging at 72%   - This completion rate (and not the completion of the counselling calls) was significantly correlated with weight loss (p=0.04) - 70.3% of the intervention participants made of the provided (free) fitness membership, with 40.7% of participants making use of it more than once |
| I-6 | Powell-Wiley et al. 2022 [9] | Research | **Study design:** Computer simulation of the users’ behavior  **Participants (N):** Not tested with real users  **Stage of technology development:** Prototype  **Setting:** Lab setting  **Outcomes:** System verification **Cost analysis:** Not conducted | - Not defined |
| I-7 | Foley et al. 2016 [10] | - | Protocol |  |
|  | Bennett et al. 2018 [11] | Deployment | **Study design:** RCT  **Participants (N):** 351  **Stage of technology development:** Post-prototype  **Setting:** Relevant environment  **Outcomes:** Health outcomes, behavioral changes, engagement **Cost analysis:** Discuss cost benefits and mention that the costs will also be formally analyzed (perhaps in a different publication) | **Metrics:** Completion of self-monitoring, coaching calls and self-weighing  **Results:**  The authors report that, on a weekly basis, the intervention participants completed:   - a median of 93.2% (IQR, 54%–100%) of self-monitoring, - a median of 89% (IQR, 50%–100%) of coaching calls, and that - participants weighed themselves on average 42.9% of the expected days per week - The authors also report that 5% or more weight loss has been achieved by over 40% of the intervention participants and suggest a link between weight loss and high engagement levels |
| I-8 | Walthouwer et al. 2013 [12] | - | Protocol |  |
|  | Walthouwer et al. 2015 [13] | Development | **Study design:** RCT  **Participants (N):** 1419  **Stage of technology development:** Post-prototype  **Setting:** Relevant environment  **Outcomes:** Use, acceptability, potential health benefits (self-reported) **Cost analysis:** Not conducted  **Comment:** Their main aim is on evaluation of different version of the intervention to see which one would work better, especially among low-educated people. Still encountered problems with e.g., measuring physical activity levels, which had consequences on feedback to the users. | - Not defined |
| I-9 | Clark et al. 2018 [14] | - | Protocol |  |
| I-10 | Pathak et al. 2021 [15] | Development | **Study design:** Crowdsourcing, feasibility, pilot and expert review  **Participants (N):** feasibility testing (n=10), pilot testing (n=8), crowdsourcing (n=134) and expert review (by coauthors)  **Stage of technology development:** Prototype stage  **Setting:** Lab setting  **Outcomes:** Acceptability, user experience, impact evaluation **Cost analysis:** Not conducted | - Not defined |
|  | Aguilera et al. 2020 [16] | - | Protocol |  |
| I-11 | Schillinger et al. 2008 [17] | Development | **Study design:** Practical RCT  **Participants (N):** 339  **Stage of technology development:** Post-prototype stage  **Setting:** Relevant environment  **Outcomes:** Focus on evaluating the reach of the technology vs. and in-person only intervention among underrepresented (and vulnerable) groups (further research necessary to establish effectiveness) **Cost analysis:** Not conducted  **Comment:** Tested in relevant environment, but under research constraints (e.g., the nurses were guided in communication) | - Not defined |
| I-12 | Wood et al. 2015 [18] | Development | **Study design:** Pilot study  **Participants (N):** 7 participants, without a comparison group  **Stage of technology development:** Post-prototype  **Setting:** Relevant environment  **Outcomes:** Behavioral outcomes (changes in self-care activities), health literacy evaluation, diabetes knowledge user experience evaluation **Cost analysis:** Not conducted  **Comment:** Beta-testing the created app | **Metrics:** Use of the app  **Results:**   - During the intervention (2 weeks), the app was opened by some participants up to 16 times, but at least twice by all participants - The use of the app during the intervention was linked to increased diabetes knowledge and confidence in performing diabetes self-care activities (e.g., in diet curation, exercising), without interfering with other, desired activities |
| I-13 | Alian et al. 2018 [19] | Research | **Study design:** Expert verification of simulated use-case scenarios  **Participants (N):** Not tested with real users, instead digital personas were used in use case studies with expert verification  **Stage of technology development:** Prototype stage  **Setting:** Lab setting  **Outcomes:** Technology verification, evaluation of proof of concept **Cost analysis:** Not conducted | - Not defined |
| I-14 | Ramirez et al. 2016 [20] | Development | **Study design:** Qualitative study  **Participants (N):** 125  **Stage of technology development:** Pre-prototype  **Setting:** Lab setting  **Outcomes:** Acceptability, intervention preferences **Cost analysis:** Not conducted | - Not defined |
|  | Ramirez & Wu 2017 [21] | Development | **Study design:** Randomized pilot study  **Participants (N):** 42  **Stage of technology development:** Prototype  **Setting:** Relevant environment  **Outcomes:** Feasibility, usability, (early stage evaluation) **Cost analysis:** Not conducted | **Metrics:** Receipt and response to a short voice or text message  **Results:**   - All participants received the messages throughout the intervention - Participants who received text messages had an average response rate of 62.7% to messages that required a response - Participants who received voice messages (automated calls) had an average response rate of 30% - Reported barriers to engagement with the text/voice messages:   - not understanding the instructions,   - not knowing how to send text messages,   - too busy to respond,   - not given enough time to respond before the call ended,   - receiving the call in a (default) language the participant did not understand |
| I-15 | Gatwood et al. 2020 [22] | Research | **Study design:** Qualitative research  **Participants (N):** 16 participants per focus group (2x), with some overlap  **Stage of technology development:** Prototype component  **Setting:** Lab setting  **Outcomes:** Acceptability, user perspectives on the tailoring aspect of the messages **Cost analysis:** Not conducted | - Not defined |
| I-16 | Mayberry et al. 2016 [23] | Development | **Study design:** Feasibility study  **Participants (N):** 19  **Stage of technology development:** Prototype  **Setting:** Relevant environment  **Outcomes:** Engagement, user experience, behavioral outcomes, functional verification & testing (to reduce technical bugs) **Cost analysis:** Not conducted | - Not defined |
|  | Nelson et al. 2018 [24] | - | Protocol |  |
| I-17 | Glasgow et al. 2011 [25] | Development | **Study design:** RCT  **Participants (N):** 270  **Stage of technology development:** Post-prototype  **Setting:** Relevant environment  **Outcomes:** Engagement, usability, website use **Cost analysis:** Not conducted | **Metrics:** Website usage (log-ins and website components that were visited at least twice)  **Results:**   - High variation in website use with a decrease over time, with some participants stopping with use completely, while some participants still visited the website weekly (for self-monitoring features) and some almost daily - The authors linked greater website usage (particularly of the self-monitoring features) with greater dietary and physical activity improvements - No (significant) links were found with medication adherence or with biological outcomes |
|  | Glasgow et al. 2012 [26] | Development | **Study design:** Pragmatic RCT  **Participants (N):** 463  **Stage of technology development:** Post-prototype  **Setting:** Relevant environment  **Outcomes:** Health outcomes, behavioral and psychosocial outcomes, website use, effectiveness **Cost analysis:** Not conducted | **Metrics:** Website use  **Results:**   - Similarly as in the study above, the website usage decreased over time, from an average of about 11 times to less than 3 times per month between the start and end of the intervention - No differences in website use were found between intervention arms of digital intervention with or without human support |
| I-18 | Azelton et al. 2021 [27] | Development | **Study design:** Pilot RCT (not yet a large clinical study)  **Participants (N):** 45  **Stage of technology development:** Post-prototype  **Setting:** Relevant environment  **Outcomes:** Health outcomes, verification of intervention effectiveness, behavioral outcomes, engagement, compliance **Cost analysis:** Cover the costs of the intervention, also in comparison to other programs; outline the projected financial value and benefits | **Metrics:** Compliance with weekly goals, how many glucose checks were performed on a daily basis, completion rate of virtual health coaching sessions  **Results:**   - 59% of weekly goals were achieved by participants and on 36% of the goals they made some progress - Blood glucose was registered on 74% of days - Completion rate of coaching sessions: 86% - A correlation was found between improvement of biological outcomes (e.g., blood pressure or weight loss) and all of the engagement metrics - Authors report that it remains inconclusive whether the engagement contributed to the improved outcomes |
| I-19 | Karimi et al. 2020 [28] | - | Protocol | - |
|  | Karimi et al. 2023 [29] | Development | **Study design:** Qualitative study  **Participants (N):** 60  **Stage of technology development:** Post-prototype  **Setting:** Relevant environment  **Outcomes:** Feasibility, acceptability, usability **Cost analysis:** Not conducted | **Metrics:** self-reported engagement  **Results:**   - Good engagement with the intervention components reported by the participants - Still, about a half (53%) of the participants reported not fully engaging with all the intervention components - Some participants would have preferred to have more customized level of the provided information that would correspond to their pre-existing knowledge - Barriers for engagement:   - Basic content   - Content already known (not curated to pre-existing knowledge of some participants) |
| I-20 | Rowsell et al. 2015 [30] | Development | **Study design:** Qualitative study  **Participants (N):** 65  **Stage of technology development:** Prototype  **Setting:** Lab setting  **Outcomes:** Engagement, usability, acceptability  **Cost analysis:** Not conducted | - Not defined |
|  | Muller et al. 2017[31] | Development | **Study design:** International RCT  **Participants (N):** 1041  **Stage of technology development:** Post-prototype  **Setting:** Relevant environment  **Outcomes:** Health literacy outcomes, engagement **Cost analysis:** Not conducted | **Metrics:** Usage of the (web-based) intervention, to compare the interactive vs. plain-text condition. Also measured self-reported engagement: satisfaction with the intervention & likelihood of recommending it to others  **Results:**   - Significant difference (p<.001) between the two conditions in intervention usage:   - In the plain-text condition, the participants completed a higher number of the intervention sections   - However, this higher completion rate did not lead to higher improvements in health literacy - Participants that had higher health literacy had significantly higher completion rates - No significant differences were found between the two conditions in terms of self-reported engagement. - Participants in both conditions reported being highly satisfied with the intervention website and most of them would likely recommend it to others |
| I-21 | Robles et al. 2022 [32] | Research | **Study design:** Qualitative research ((1) discovery interviews, (2) empathy mapping, (3) design sprint, (4) storyboarding, and (5) paper prototyping)  **Participants (N):** n=5 (interviews), n=12 (empathy mapping), n=3 design team, n=10 prototype feedback  **Stage of technology development:** Pre-prototype and early (paper) prototypes  **Setting:** Lab setting  **Outcomes:** Acceptability, usability, opinions of the target population on first prototypes that then guided the final design decisions **Cost analysis:** Not conducted | - Not defined |
|  | Hellem et al. 2024 [33] | Research | **Study design:** Qualitative research  **Participants (N):** 86  **Stage of technology development:** Pre-prototype and Prototype component  **Setting:** Lab setting  **Outcomes:** Qualitative feedback on developed notifications **Cost analysis:** Not conducted  **Comment:** Evaluating how well the generated messages would fit (in terms of relevance) to the different communities (high and low SE  p), but there is not yet a technology involved in the evaluation, only in the concept (the intervention with the mHealth technology is drafted/designed, but the main point of this paper is evaluating the message component, i.e., they have a concept of the intervention and they're evaluating a component of the intervention to see the acceptability of it) | - Not defined |
|  | Golbus et al. 2024 [34] | - | Protocol |  |
| I-22 | Hageman et al. 2014 [35] | Development | **Study design:** RCT  **Participants (N):** 289  **Stage of technology development:** Post-prototype  **Setting:** Relevant environment  **Outcomes:** Usability, health outcomes, behavioral changes **Cost analysis:** Not conducted | - Not defined |
| I-23 | Ayre et al. 2019 [36] | - | Protocol |  |
|  | Ayre et al. 2020 [37] | Development | **Study design:** Online RCT  **Participants (N):** 1,769  **Stage of technology development:** Post-prototype  **Setting**: Real-world  **Outcomes:** Usability, behavioral outcomes (self-reported reduction in snacking behavior) **Cost analysis:** Not conducted | - Not defined |
| I-24 | Tamura et al. 2020 [38] | - | Protocol |  |
|  | Vijayakumar et al. 2022 [39] | Development | **Study design:** Pilot study (Community-based pilot feasibility intervention)  **Participants (N):** 24  **Stage of technology development:** Post-prototype  **Setting:** Relevant environment  **Outcomes:** Usability, function verification **Cost analysis:** Not conducted  **Comment:** Changes were still made to the functions of the app, it was not a final version | **Metrics:** Scores based on user-app interaction  **Results:**   - Average user-app engagement score was 9.8/18 points - Average completion rates:   - EMAs: 37.5% rate of completing all EMAs and 62.5% rate of completing at least half   - All modules: 62.5%   - Goal-setting tools: 12.5% - Better social connectedness within the neighborhood, higher neighborhood safety, but also higher social isolation were linked to higher engagement levels |
| I-25 | Greaney et al. 2009 [40] | - | Protocol | - |
|  | Bennett et al. 2012 [41] | Deployment | **Study design:** Randomized pragmatic/effectiveness trial  **Participants (N):** 365  **Stage of technology development:** Post-prototype  **Setting:** Relevant environment (Clinical setting)  **Outcomes:** Health outcomes and engagement **Cost analysis:** Not conducted | **Metrics:** Completion rates of counselling calls, goal tracking  **Results:**   - Overall average completion rate of the counselling calls amounted to 70%, with completion declining from 80% to 66.7% between the initial and last counselling calls - 40% of participants tracked their goals on a weekly basis for at least half of the intervention, while 25% did this for at least 75% of the intervention duration - The authors report that engagement was not at the desired levels |
| I-26 | Diez-Canseco et al. 2015 [42] | Development | **Study design:** Feasibility & acceptability study (mixed-methods)  **Participants (N):** 108  **Stage of technology development:** Prototype component  **Setting:** Lab setting  **Outcomes:** Acceptability (understanding and appeal) **Cost analysis:** Not conducted | - Not defined |
|  | Rubinstein et al. 2016 [43] | Deployment | **Study design:** RCT  **Participants (N):** 637  **Stage of technology development:** Post-prototype  **Setting:** Relevant environment  **Outcomes:** Health outcomes, self-reported behavioral outcomes **Cost analysis:** Cost effectiveness analyzed, reported elsewhere | - Not defined |
| I-27 | Schultz et al. 2022 [44] | - | Protocol | - |

IVR, Interactive Voice Response; EMA, Ecological Momentary Assessment

* Intervention ID

** Pre-prototype (research, no technology/interactive prototype developed yet); Prototype or prototype component(s); Post-prototype (moving towards a more mature application)

[1]	Jansen-Kosterink S, Broekhuis M, van Velsen L. Time to act mature—Gearing eHealth evaluations towards technology readiness levels. Digit Health 2022;8:20552076221113396. https://doi.org/10.1177/20552076221113396.

[2]	Dombrowski SU, McDonald M, Van Der Pol M, Grindle M, Avenell A, Carroll P, et al. Game of Stones: feasibility randomised controlled trial of how to engage men with obesity in text message and incentive interventions for weight loss. BMJ Open 2020;10:e032653. https://doi.org/10.1136/bmjopen-2019-032653.

[3]	Macaulay L, O’Dolan C, Avenell A, Carroll P, Cotton S, Dombrowski S, et al. Effectiveness and cost-effectiveness of text messages with or without endowment incentives for weight management in men with obesity (Game of Stones): study protocol for a randomised controlled trial. Trials 2022;23:582. https://doi.org/10.1186/s13063-022-06504-5.

[4]	Stein RI, Strickland JR, Tabak RG, Dale AM, Colditz GA, Evanoff BA. Design of a randomized trial testing a multi-level weight-control intervention to reduce obesity and related health conditions in low-income workers. Contemp Clin Trials 2019;79:89–97. https://doi.org/10.1016/j.cct.2019.01.011.

[5]	Kharmats AY, Wang C, Fuentes L, Hu L, Kline T, Welding K, et al. Monday-focused tailored rapid interactive mobile messaging for weight management 2 (MTRIMM2): results from a randomized controlled trial br. MHEALTH 2022;8. https://doi.org/10.21037/mhealth-21-3.

[6]	Berger MB, Steinberg DM, Askew S, Gallis JA, Treadway CC, Egger JR, et al. The Balance protocol: A pragmatic weight gain prevention randomized controlled trial for medically vulnerable patients within primary care. BMC Public Health 2019;19. https://doi.org/10.1186/s12889-019-6926-7.

[7]	Foley P, Levine E, Askew S, Puleo E, Whiteley J, Batch B, et al. Weight gain prevention among black women in the rural community health center setting: The Shape Program. BMC Public Health 2012;12. https://doi.org/10.1186/1471-2458-12-305.

[8]	Bennett GG, Foley P, Levine E, Whiteley J, Askew S, Steinberg DM, et al. Behavioral Treatment for Weight Gain Prevention Among Black Women in Primary Care Practice: A Randomized Clinical Trial. JAMA Intern Med 2013;173:1770. https://doi.org/10.1001/jamainternmed.2013.9263.

[9]	Powell-Wiley TM, Martinez MF, Tamura K, Neally SJ, O’Shea KJ, Curlin K, et al. The Impact of a Place-Tailored Digital Health App Promoting Exercise Classes on African American Women’s Physical Activity and Obesity: Simulation Study. J Med Internet Res 2022;24. https://doi.org/10.2196/30581.

[10]	Foley P, Steinberg D, Levine E, Askew S, Batch BC, Puleo EM, et al. Track: A randomized controlled trial of a digital health obesity treatment intervention for medically vulnerable primary care patients. Contemp Clin Trials 2016;48:12–20. https://doi.org/10.1016/j.cct.2016.03.006.

[11]	Bennett GG, Steinberg D, Askew S, Levine E, Foley P, Batch BC, et al. Effectiveness of an App and Provider Counseling for Obesity Treatment in Primary Care. Am J Prev Med 2018;55:777–86. https://doi.org/10.1016/j.amepre.2018.07.005.

[12]	Walthouwer MJL, Oenema A, Soetens K, Lechner L, De Vries H. Systematic development of a text-driven and a video-driven web-based computer-tailored obesity prevention intervention. BMC Public Health 2013;13. https://doi.org/10.1186/1471-2458-13-978.

[13]	Walthouwer MJL, Oenema A, Lechner L, de Vries H. Comparing a Video and Text Version of a Web-Based Computer-Tailored Intervention for Obesity Prevention: A Randomized Controlled Trial. J Med INTERNET Res 2015;17. https://doi.org/10.2196/jmir.4083.

[14]	Clark DO, Srinivas P, Bodke K, Keith N, Hood S, Tu W. Addressing people and place microenvironments in weight loss disparities (APP-Me): Design of a randomized controlled trial testing timely messages for weight loss behavior in low income Black and White Women. Contemp Clin Trials 2018;67:74–80. https://doi.org/10.1016/j.cct.2018.01.006.

[15]	Pathak LE, Aguilera A, Williams JJ, Lyles CR, Hernandez-Ramos R, Miramontes J, et al. Developing Messaging Content for a Physical Activity Smartphone App Tailored to Low-Income Patients: User-Centered Design and Crowdsourcing Approach. JMIR MHealth UHealth 2021;9:e21177. https://doi.org/10.2196/21177.

[16]	Aguilera A, Figueroa CA, Hernandez-Ramos R, Sarkar U, Cemballi A, Gomez-Pathak L, et al. mHealth app using machine learning to increase physical activity in diabetes and depression: clinical trial protocol for the DIAMANTE Study. BMJ Open 2020;10:e034723. https://doi.org/10.1136/bmjopen-2019-034723.

[17]	Schillinger D, Hammer H, Wang F, Palacios J, McLean I, Tang A, et al. Seeing in 3-D: Examining the reach of diabetes self-management support strategies in a public health care system. Health Educ Behav 2008;35:664–82. https://doi.org/10.1177/1090198106296772.

[18]	Wood FG, Alley E, Baer S, Johnson R. Interactive Multimedia Tailored to Improve Diabetes Self-Management. Nurs Clin North Am 2015;50:565-+. https://doi.org/10.1016/j.cnur.2015.05.009.

[19]	Alian S, Li J, Pandey V. A Personalized Recommendation System to Support Diabetes Self-Management for American Indians. IEEE Access 2018;6:73041–51. https://doi.org/10.1109/ACCESS.2018.2882138.

[20]	Ramirez M, Wu S, Beale E. Designing a Text Messaging Intervention to Improve Physical Activity Behavior Among Low-Income Latino Patients With Diabetes: A Discrete-Choice Experiment, Los Angeles, 2014-2015. Prev Chronic Dis 2016;13. https://doi.org/10.5888/pcd13.160035.

[21]	Ramirez M, Wu S. Phone messaging to prompt physical activity and social support among low-income latino patients with type 2 diabetes: A randomized pilot study. JMIR Diabetes 2017;2. https://doi.org/10.2196/diabetes.7063.

[22]	Gatwood J, Shuvo S, Ross A, Riordan C, Smith P, Gutierrez ML, et al. The Management of Diabetes in Everyday Life (MODEL) program: development of a tailored text message intervention to improve diabetes self-care activities among underserved African-American adults. Transl Behav Med 2020;10:204–12. https://doi.org/10.1093/tbm/ibz024.

[23]	Mayberry LS, Berg CA, Harper KJ, Osborn CY. The Design, Usability, and Feasibility of a Family-Focused Diabetes Self-Care Support mHealth Intervention for Diverse, Low-Income Adults with Type 2 Diabetes. J Diabetes Res 2016;2016:e7586385. https://doi.org/10.1155/2016/7586385.

[24]	Nelson LA, Wallston KA, Kripalani S, Greevy RA, Elasy TA, Bergner EM, et al. Mobile phone support for diabetes self-care among diverse adults: Protocol for a three-arm randomized controlled trial. JMIR Res Protoc 2018;7. https://doi.org/10.2196/resprot.9443.

[25]	Glasgow RE, Christiansen SM, Kurz D, King DK, Woolley T, Faber AJ, et al. Engagement in a diabetes self-management website: Usage patterns and generalizability of program use. J Med Internet Res 2011;13. https://doi.org/10.2196/jmir.1391.

[26]	Glasgow RE, Kurz D, King D, Dickman JM, Faber AJ, Halterman E, et al. Twelve-month outcomes of an Internet-based diabetes self-management support program. Patient Educ Couns 2012;87:81–92. https://doi.org/10.1016/j.pec.2011.07.024.

[27]	Azelton KR, Crowley AP, Vence N, Underwood K, Morris G, Kelly J, et al. Digital Health Coaching for Type 2 Diabetes: Randomized Controlled Trial of Healthy at Home. Front Digit Health 2021;3. https://doi.org/10.3389/fdgth.2021.764735.

[28]	Karimi N, Crawford D, Opie R, Maddison R, O’Connell S, Hamblin PS, et al. EatSmart, a Web-Based and Mobile Healthy Eating Intervention for Disadvantaged People With Type 2 Diabetes: Protocol for a Pilot Mixed Methods Intervention Study. JMIR Res Protoc 2020;9:e19488. https://doi.org/10.2196/19488.

[29]	Karimi N, Opie R, Crawford D, O’Connell S, Hamblin PS, Steele C, et al. Participants’ and Health Care Providers’ Insights Regarding a Web-Based and Mobile-Delivered Healthy Eating Program for Disadvantaged People With Type 2 Diabetes: Descriptive Qualitative Study. JMIR Form Res 2023;7:e37429. https://doi.org/10.2196/37429.

[30]	Rowsell A, Muller I, Murray E, Little P, Byrne CD, Ganahl K, et al. Views of People With High and Low Levels of Health Literacy About a Digital Intervention to Promote Physical Activity for Diabetes: A Qualitative Study in Five Countries. J Med Internet Res 2015;17:e4999. https://doi.org/10.2196/jmir.4999.

[31]	Muller I, Rowsell A, Stuart B, Hayter V, Little P, Ganahl K, et al. Effects on engagement and health literacy outcomes of web-based materials promoting physical activity in people with diabetes: An international randomized trial. J Med Internet Res 2017;19. https://doi.org/10.2196/jmir.6601.

[32]	Robles MC, Newman MW, Doshi A, Bailey S, Huang L, Choi SJ, et al. A Physical Activity Just-in-time Adaptive Intervention Designed in Partnership With a Predominantly Black Community: Virtual, Community-Based Participatory Design Approach. JMIR Form Res 2022;6. https://doi.org/10.2196/33087.

[33]	Hellem AK, Casetti A, Bowie K, Golbus JR, Merid B, Nallamothu BK, et al. A Community Participatory Approach to Creating Contextually Tailored mHealth Notifications: myBPmyLife Project. Health Promot Pract 2024;25:417–27. https://doi.org/10.1177/15248399221141687.

[34]	Golbus JR, Jeganathan VSE, Stevens R, Ekechukwu W, Farhan Z, Contreras R, et al. A physical activity and diet just-in-time adaptive intervention to reduce blood pressure: The myBPmyLife study rationale and design. J Am HEART Assoc 2024;13. https://doi.org/10.1161/JAHA.123.031234.

[35]	Hageman PA, Pullen CH, Hertzog M, Boeckner LS. Effectiveness of tailored lifestyle interventions, using web-based and print-mail, for reducing blood pressure among rural women with prehypertension: main results of the Wellness for Women: DASHing towards Health clinical trial. Int J Behav Nutr Phys Act 2014;11. https://doi.org/10.1186/s12966-014-0148-2.

[36]	Ayre J, Cvejic E, Bonner C, Turner RM, Walter SD, McCaffery KJ. Accounting for health literacy and intervention preferences when reducing unhealthy snacking: protocol for an online randomised controlled trial. BMJ Open 2019;9:e028544. https://doi.org/10.1136/bmjopen-2018-028544.

[37]	Ayre J, Cvejic E, Bonner C, Turner RM, Walter SD, McCaffery KJ. Effects of health literacy, screening, and participant choice on action plans for reducing unhealthy snacking in Australia: A randomised controlled trial. PLOS Med 2020;17. https://doi.org/10.1371/journal.pmed.1003409.

[38]	Tamura K, Vijayakumar NP, Troendle JF, Curlin K, Neally SJ, Mitchell VM, et al. Multilevel mobile health approach to improve cardiovascular health in resource-limited communities with Step It Up: a randomised controlled trial protocol targeting physical activity. BMJ Open 2020;10:e040702. https://doi.org/10.1136/bmjopen-2020-040702.

[39]	Vijayakumar NP, Neally SJ, Potharaju KA, Curlin K, Troendle JF, Collins BS, et al. Customizing Place-Tailored Messaging Using a Multilevel Approach: Pilot Study of the Step It Up Physical Activity Mobile App Tailored to Neighborhood Environment. Circ Cardiovasc Qual Outcomes 2022;15:E009328. https://doi.org/10.1161/CIRCOUTCOMES.122.009328.

[40]	Greaney ML, Quintiliani LM, Warner ET, King DK, Emmons KM, Colditz GA, et al. Weight Management Among Patients at Community Health Centers: The “Be Fit, Be Well” Study. Obes Weight Manag 2009;5:222–8. https://doi.org/10.1089/obe.2009.0507.

[41]	Bennett GG, Warner ET, Glasgow RE, Askew S, Goldman J, Ritzwoller DP, et al. Obesity treatment for socioeconomically disadvantaged patients in primary care practice. Arch Intern Med 2012;172:565–74. https://doi.org/10.1001/archinternmed.2012.1.

[42]	Diez-Canseco F, Zavala-Loayza JA, Beratarrechea A, Kanter R, Ramirez-Zea M, Rubinstein A, et al. Design and Multi-Country Validation of Text Messages for an mHealth Intervention for Primary Prevention of Progression to Hypertension in Latin America. JMIR MHealth UHealth 2015;3:e3874. https://doi.org/10.2196/mhealth.3874.

[43]	Rubinstein A, Miranda JJ, Beratarrechea A, Diez-Canseco F, Kanter R, Gutierrez L, et al. Effectiveness of an mHealth intervention to improve the cardiometabolic profile of people with prehypertension in low-resource urban settings in Latin America: a randomised controlled trial. Lancet Diabetes Endocrinol 2016;4:52–63. https://doi.org/10.1016/S2213-8587(15)00381-2.

[44]	Schultz CL, Bocarro JN, Hipp JA, Bennett GJ, Floyd MF. Prescribing time in nature for human health and well-being: Study protocol for tailored park prescriptions. Front Digit Health 2022;4. https://doi.org/10.3389/fdgth.2022.932533.
