# Supplementary material for: Tailoring in eHealth lifestyle interventions targeting people with cardiometabolic conditions and lower socioeconomic position: A scoping review
Source: PLOS Digit Health. 2026 Sep 11;5(9):e0001713. doi: 10.1371/journal.pdig.0001713 (PMC13568521; doi:10.1371/journal.pdig.0001713)
Supplement: Supplementary file 5 [file pdig.0001713.s006.docx]

Response to Reviewers

[PDIG-D-25-00409] Tailoring in eHealth lifestyle interventions targeting people with cardiometabolic conditions and lower socioeconomic position: a scoping review

Dear Aline Lutz de Araujo, Leo Anthony Celi and reviewers,

Thank you for reviewing our manuscript. We appreciated the reviewers’ positive assessment of the paper’s relevance, rigor and contributions to the field. Based on their comments, we carefully revised the manuscript, and also worked with a language editor to improve our narrative synthesis, linguistic clarity and conciseness.

In the table below, we individually address all points raised by the reviewers and we refer to changes in the tracked manuscript.

We trust that our revisions have adequately addressed all comments but would be willing to make further adjustments if required.

| # | Reviewer #1 | Response to Reviewer #1 | Manuscript page and line number |
| --- | --- | --- | --- |
| 1.1 | Your review provides synthesis of tailoring approaches used in eHealth interventions targeting low-SEP populations with cardiometabolic conditions. It also findings also uncover several gaps and opportunities for further research for eHealth lifestyle interventions in lower socioeconomic population having cardiometabolic conditions. | Thank you |  |
|  | Reviewer #2 | Response to Reviewer #2 | Manuscript page and line number |
| 2.1 | Overall Evaluation  This manuscript presents a comprehensive scoping review examining tailoring approaches within eHealth lifestyle interventions for individuals with cardiometabolic conditions and lower socioeconomic positions (SEP). The paper is well-written, methodologically sound, and addresses a relevant public health issue. It maps the current landscape of tailoring strategies in eHealth interventions, emphasizing gaps and future research directions.  Strengths Relevance and Timeliness o The focus on tailoring eHealth interventions for lower SEP populations is highly relevant, considering the health disparities and digital divide in chronic disease management. o Cardiometabolic conditions are a critical global health burden, making the topic of this review particularly impactful.  Contributions (should be improved – see below) o The paper identifies gaps in existing interventions (e.g., overreliance on rule-based algorithms - that are quite old and limited evaluation of tailoring). o It provides actionable insights for researchers, including the need for dynamic, adaptive tailoring using computational algorithms.  Innovation Potential (should be improved – see below) o By highlighting the underutilization of AI-based or adaptive tailoring techniques, the paper sets a foundation for innovative digital health solutions. | Thank you |  |
| 2.2 | Comprehensive Methodology (should be improved) o The scoping review follows the Arksey and O’Malley framework and PRISMA-ScR checklist, demonstrating rigor and transparency. Yet, many papers are pre GenAI epoch and therefore their eHealth tailoring approach and recommendations could be extrapolated to better reflect the abilities nowadays. | Thank you, we extended the discussion section to provide more examples on technology-based tailoring, with emphasis on Large Language Models and GenAI:  *“[…]This means interventions can draw on more data (e.g., behavioral, contextual, feedback, preferences), increase their granularity and dynamic adaptivity, and limit additional manual development. For example, an RL model can learn from the users’ actions and adapt elements such as goals, accordingly over time. Moreover, with a broader knowledge bank, a recommender system could suggest PA types that also accommodate community resources, thereby enhancing applicability for varying SEP levels. Such methods facilitate considerably more advanced tailoring approaches that future research could explore, though generative AI, including Large Language Models (LLMs), widens this scope even further.*  *The recent paradigm shift in the competence of LLMs, such as ChatGPT, has enabled automatic content generation, streamlining intervention development and enabling deeper tailoring. For example, creating content that is directly tailored to an individual can be challenging. However, LLMs, can leverage user-specific information to generate fully individualized content, including text and images, in real-time, supporting functions such as feedback selection, variable substitution, or recommendation provision [107]. LLMs offer greater flexibility and could facilitate SEP-specific tailoring that moves away from by-design approaches. For example, adapting language complexity, length or tone of text to match language skills, or community preferences are important tailoring aspects for this target population. While further research is required to ensure their trustworthiness, LLMs could dynamically tailor these features, through user profiles or interactive collaboration. Furthermore, opportunities remain to generate tailored images, automatically translate text or have it read aloud in custom voice to enhance accessibility and sociocultural relevance. With a careful design of the LLM’s capabilities, e.g., ensuring that it remains within approved medical guidelines to mitigate inaccurate information, this area of research holds a promising direction for more sophisticated tailoring methods.“* | p. 33-34 , lines 548-574 |
| 2.3 | o A broad search strategy across multiple databases (Scopus, Web of Science, PubMed, PsycINFO) ensures thorough coverage of relevant literature (I am not sure if all the data is available to the readers – please indicate clearly) | We added a link to the data availability statement that provides access to the data extraction sheet and the papers excluded during full-text screening. |  |
| 2.4 | Clarity of Presentation o The manuscript is logically structured, with detailed tables and figures summarizing interventions (quality of some figures should be improved!), tailoring variables, and algorithm types. | Thank you, we improved the quality of figures 2, 4 and 5.  In Figure 2, we changed the order of the colors in the bars, so that the green (majority) color is always at the bottom and we added a grid for improved readability:    In Figures 4 and 5, we better highlighted each stage/border, increased the size and boldness of the text, we removed the white border background behind each label and we spread the labels out a bit more for easier visual processing:   |  |
| 2.5 | o The discussion connects findings to practical implications and future research needs yet should be further extended. | We extended the discussion section with additional GenAI tailoring suggestions, see point 2.2 above. | p. 33-34 , lines 548-574 |
| 2.6 | Weaknesses  Limited Critical Analysis of Intervention Quality o While the paper maps tailoring approaches, there is little discussion about the quality or effectiveness of the interventions, beyond descriptive reporting. | Thank you for this comment. We did not conduct quality assessment in our review because we followed the guidelines of Arksey & o’Malley [1], Peters et al. [2], and Pollock et al. [3], which delineate that the aim of scoping reviews should be to present and bring together existing records regardless of their methodology or quality.  We amended the Methods section to clarify this:  *“We chose to conduct a scoping review to identify research gaps [35,36] and to examine the existing evidence without requirements on quality assessment and restrictions of the included study designs [36].”*  Effectiveness analysis was not feasible, as most interventions did not evaluate the effects of tailoring specifically, outside the effects of the intervention, or did not evaluate tailoring at all. Our findings highlight the need for more systematic evaluations and standardized processes to enable future effectiveness assessments. | Page 6, lines 125-127 |
| 2.7 | o More critical analysis of the success (or failure) of specific tailoring strategies would strengthen the contribution. | Thank you. We did not include such analyses, as most interventions did not evaluate the effects of tailoring specifically, outside the effects of the intervention, only evaluated it qualitatively, or did not evaluate it at all. We clarified this for future work:  “*Together with more comprehensive reporting on the used tailoring algorithms, these steps can ensure reproducibility and facilitate stronger analyses of the outcomes of different tailoring methods.*” | p. 35, lines 620-622 |
| 2.8 | Innovation Gap in Findings o Although the paper is comprehensive, the review largely synthesizes existing literature without proposing a conceptual framework or new model for tailoring. This is particularly true when considering that many of the reviewed works are pre GenAI epoch, and that the possibility to implement new approaches – granular and personal – are much higher nowadays vs. 10 years ago! For example, mentioning two papers from 2015 and 2019 [68,79], i.e. pre GenAI, that propose "straightforward language with layman terms and positive framing" can be accompanied by new possibilities of AI these days.  o The automated procedure for screening and selecting relevant papers is systematic and thorough, however the outcome of extracting information from those papers can be elevated. The integration of relevant modern approaches to generate new ideas and policies is required.  o Following the above, a more explicit discussion of how emerging technologies (e.g., GenAI, personalization engines etc) could be integrated might add novelty. | Thank you. Adding a conceptual framework goes beyond the scope of our review, but we extended the discussion section with additional GenAI tailoring suggestions, see point 2.2 above. | p. 33-34 , lines 548-574 |
| 2.9 | Overemphasis on Descriptive Statistics o The results section is heavily descriptive (e.g., counts of studies), which sometimes overshadows interpretative insights. A stronger narrative synthesis, including potential effectiveness of the recommendation, could improve readability and impact. | Thank you, please see the tracked changes in the results section. In addressing this suggestion, we aimed to keep a balance between the descriptive and narrative components of the results section. | Results section, p. 11-30 |
| 2.10 | Evaluation of Tailoring o The paper highlights that over half of interventions lacked evaluation of tailoring, but it does not provide in-depth recommendations or criteria for how evaluation should be conducted. | We extended the section on tailoring evaluation in the discussion section with example recommendations:  *“For example, to isolate the effects of tailoring, the comparator should not be a fundamentally different intervention. Oftentimes, tailored interventions are compared against routine care, which obscures the exact value that tailoring contributes to the overall effectiveness of the new intervention. Rather, the tailored intervention should also be compared against a non-tailored counterpart, next to routine care. In this way, a baseline effect can be determined of the new approach over established procedures, as well as what added effects the tailoring provides. Thus, future research should evaluate tailoring approaches, for example via micro-randomized trials [113], by comparing the tailored system to a non-tailored one during a trial, or A/B testing of tailored variants.”* | p. 35, lines 611-620 |
| 2.11 | Minor Scope Limitation  o Many of the papers focus on diet and physical activity recommendations - this should be reflected in the abstract and conclusions – thus, narrowing the claimed generality. The exclusion of certain behavioral domains (e.g., mental health or smoking cessation) is understandable but may limit the generalizability of conclusions about tailoring methods. | We modified the Abstract and Conclusion sections to clarify the scope, as follows:   - Abstract: “*We identified 43 eligible articles, with 27 unique eHealth lifestyle interventions targeting primarily diet and exercise.”* - Conclusion: “*This review provides a synthesis of tailoring approaches used in eHealth interventions targeting primarily diet and exercise in low-SEP populations with cardiometabolic conditions. […] The overview of tailoring levels, tailoring variables, and participant perspectives provides useful pointers for design of diet- and exercise-focused interventions with tailoring for low-SEP populations.”* | Abstract: p. 2, line 39  Conclusion: p. 37, lines 654 and 664 |
| 2.12 | The manuscript makes a relevant contribution to the field of digital health by systematically mapping tailoring approaches for vulnerable populations. With some improvements in critical analysis, the addition of a conceptual framework or more future-oriented recommendations, and a slightly less descriptive tone, the paper could be an excellent addition to PLOS Digital Health. | Thank you |  |
|  | Reviewer #3 | Response to Reviewer #3 | Manuscript page and line number |
| 3.1 | Strengths  Clear scope focusing on low-SEP populations; avoids race/ethnicity as a sole SEP proxy. Transparent and updated search methodology. Effective conceptual mapping of tailoring and algorithmic functions; prevents double-counting by merging multi-paper records. Acknowledges the frequent lack of tailoring evaluation in literature. | Thank you |  |
| 3.2 | Dual Screening and Coding: Expand QA beyond 20% title/abstract double-screening. Conduct and report independent duplicate full-text screening and coding for a robust subset (e.g., ≥25–30%), including κ statistics and resolution procedures. | Thank you for the comment. We reported the Cohen’s kappa for title and abstract screening (κ=0.9) (p. 7, line 150). The second author additionally screened 20% of full-text articles for verification. We reported the Cohen’s kappa (κ=0.9) (p. 8, line 156). However, due to restricted available resources, we were not able to do more than 20% of double-screening or additional coding. We chose this percentage because it is consistent with methods used in other reviews. For transparency, we published the papers excluded during full-text screening, together with the reasons for their exclusion (see point 3.4).  We clarified our resolution procedures:   - “*The reviewers met at the mid-point and at the end of the screening process to resolve any conflicts, following the procedure of Mark and Thomas [41] and Levac et al. [42]. Any additional doubts were consulted with the other authors until consensus.*” (p. 7, lines 150-153) - “*Any questions or uncertainties that emerged during the coding process were discussed with the other authors until consensus was reached*.” (p.10, lines 201-203)   We added clarifications for the coding process:  “*This categorization*” [of TRL levels] “*was independently verified by the second reviewer (MK)*.” (p.9. 185-186) | p. 7, line 150  p. 8, line 156  p. 7, lines 150-153  p.10, lines 201-203  p.9. 185-186 |
| 3.3 | Algorithm Classification Transparency: Explicitly flag inferred algorithm types/functions in tables/figures with textual evidence or "not reported." Consider a "Reported by authors" vs. "Inferred by reviewers" column. | Thank you, we added a column next to algorithm type in the extraction spreadsheet, which we made publicly available (see point 3.4).  We added the following clarification to the title of Table 8:  *“Definitions of the identified thematic clusters for the functions of algorithms used for the execution of tailoring within the included eHealth interventions, inferred from the article’s intervention descriptions.”* | p. 25, lines 405-406 |
| 3.4 | Data Availability: Deposit the complete study-level extraction spreadsheet and full list of excluded full-text records with reasons in an open repository (DOI preferred). | The requested data were deposited in OSF: DOI: https://doi.org/10.17605/OSF.IO/3AFSR. We added the link to the data availability statement. |  |
| 3.5 | Denominators and Sensitivity Analyses:  - Report counts and denominators for evaluation practices/algorithm types. | Counts and denominators have been added in the Results section where only percentages were being reported. You can find these in tracked changes on the given pages. | p. 11, 14, 15, 16, 25, 26, 28 |
| 3.6 | - Consider sensitivity analysis excluding interventions without deployed evaluation. | Thank you for this comment, however, applying sensitivity analysis to all our results would not be applicable because, rather than considering the effects of tailoring approaches, we wanted to explore all the approaches that exist. In the *Evaluation of tailoring* section, interventions without deployed evaluations were indeed excluded, as they did not describe any evaluation results. We clarified this exclusion as follows:  *“Twelve of the included interventions (n=12/27, 44%) evaluated the tailoring within the tailored intervention elements […]. Most of these studies […]”* | Evaluation of tailoring: p. 28-30  Clarification: p. 28, line 441 |
| 3.7 | Limitations: Discuss potential selection bias from including protocols/design papers and temper language in the Abstract/Conclusion accordingly. | To address this point, the following text was added to the Strengths and Limitations section:  *“Thirdly, four of the included studies were standalone protocols without evaluation. This could mean that the tailoring approaches within these protocols were more ambitious and might not be feasible to implement in a real-world scenario. Nevertheless, these studies showcase the intended tailoring and might serve as an inspiration for future work.”*  We also amended the language in the Abstract and the Conclusion:   - *Abstract: “These findings suggest that while tailoring is present in many interventions, the current approaches remain limited in the facilitating technology and dynamic adaptations to SEP-specific needs.”* - Conclusion: *“The evaluations seem to suggest a positive impact of health literacy-sensitive design and highlighted the value of (audio-)visual materials and of sociocultural relevance.”* | Strengths and Limitations section: p. 36, lines 643-646  Abstract: p. 3, line 49  Conclusion: p.37, lines 659-660 |
| 3.8 | Minor Suggestions: State whether full-text screening and data extraction involved independent duplication and where details are located. | We added the statements as follows:   - *“SS subsequently screened all full texts, with MK independently screening 20% (κ=0.9) for verification.”* - *“The extraction process was thoroughly discussed with the other co-authors and piloted by SS and MK, to ensure clarity and consistency, and then conducted by SS.”*   For details on the full-text screening, please see the point 3.2 above. | p. 8, lines 155-156  p.8, lines 164-166 |
| 3.9 | Ensure consistent use of n=27 interventions vs n=43 papers across all percentages. | We included the denominator of n=x/27 or n=x/43 in all the appropriate instances where percentages are provided. This can be found in the tracked changes of the manuscript. | p. 14, 15, 16, 25, 26, 28 |
| 3.10 | Consider a figure to visually separate by-design vs in-intervention dynamic tailoring. | Thank you, we created Figure 3 as follows:     |  |

[1]	Arksey H, O’Malley L. Scoping studies: towards a methodological framework. Int J Soc Res Methodol 2005;8:19–32. https://doi.org/10.1080/1364557032000119616.

[2]	Peters MDJ, Godfrey CM, Khalil H, McInerney P, Parker D, Soares CB. Guidance for conducting systematic scoping reviews. Int J Evid Based Healthc 2015;13:141–6. https://doi.org/10.1097/XEB.0000000000000050.

[3]	Pollock D, Peters MDJ, Khalil H, McInerney P, Alexander L, Tricco AC, et al. Recommendations for the extraction, analysis, and presentation of results in scoping reviews. JBI Evid Synth 2023;21:520–32. https://doi.org/10.11124/JBIES-22-00123.
