# Supplementary material for: Tailoring in eHealth lifestyle interventions targeting people with cardiometabolic conditions and lower socioeconomic position: A scoping review
Source: PLOS Digit Health. 2026 Sep 11;5(9):e0001713. doi: 10.1371/journal.pdig.0001713 (PMC13568521; doi:10.1371/journal.pdig.0001713)
Supplement: Supplementary file 6 [file pdig.0001713.s007.docx]

Response to Reviewers

[PDIG-D-25-00409R1] Tailoring in eHealth lifestyle interventions targeting people with cardiometabolic conditions and lower socioeconomic position: a scoping review

Dear Onicio Leal-Neto, Leo Anthony Celi and reviewers,

Thank you for reviewing our manuscript. We appreciated the reviewers’ positive assessment of our previous revision and the reinforcement of our paper’s rigor and importance in the field. We carefully revised the manuscript and in the table below, we individually address all points raised by the reviewers. In this table, we also refer to the pages and lines where the changes can be found in the tracked manuscript (with track changes hidden).

| # | Reviewer #2 comments | Response to Reviewer #2 | Page and line numbers (with changes **hidden)** |
| --- | --- | --- | --- |
| 2.1 | This review maps how tailoring is used in eHealth lifestyle interventions for adults with cardiometabolic conditions and lower socioeconomic position (SEP). The topic is important, and the review is conducted with reasonable methodological approach. However, in its current form, the manuscript does not yet reach the level of conceptual and practical maturity expected for PLOS ONE. | Thank you |  |
| 2.2 | -Limited added value beyond descriptive mapping: While the descriptive overview is solid, the paper stops short of offering a conceptual framework that truly links: e.g. tailoring level → chosen algorithmic approach → required data → expected mechanisms → appropriate evaluation design. Without such structure, the significance of the review for guiding future eHealth tailoring research is reduced. | Thank you, we added a conceptual framework that links the key concepts identified within our results, together with future work implications, highlighting gaps/underexplored areas, as well as important considerations for the low SEP population in the algorithmic context. Moreover, we also include examples from two reviewed interventions, that show how they traverse the framework. The discussion section is grounded in this visual framework, which aids in presenting existing knowledge and approaches and eases identification of important considerations and research opportunities.    ***Fig 6. A conceptual framework, connecting the results of this review, with research implications and envisioned future work.*** *The red squares point to opportunities, such as further exploration of implementing user-based, adaptive algorithms as methods for tailoring, as well as the need for evaluations of different tailoring approaches. The orange squares highlight important considerations when designing with AI technologies for low-SEP groups. With the green lines, we included two examples from the reviewed interventions, to show how the existing interventions traverse this framework. We also included an envisioned example of which components a future intervention could contain. We aimed for representative example methods under e.g., evaluations or adaptive algorithms, though these lists are not meant to be exhaustive. CBPR: Community-based participatory research.*  We also highlighted the added framework in our abstract and conclusion:   - Abstract: “*To guide future tailoring designs, we assembled a framework that encapsulates the key concepts from our findings, in combination with envisioned future work.*” - Conclusion: “*To guide future interventions, we assembled the key identified gaps and concepts from our review into a visionary framework, to help expand the existing tailoring approaches and to highlight key considerations in the design process of tailoring.*” | p. 29 (and in figure files)  Abstract:  p.2  lines 45-47  Conclusion:  p. 36  lines 644-647 |
| 2.3 | -Insufficient engagement with implications for low SEP in the algorithmic context. The manuscript notes the importance of tailoring to literacy, culture, and SEP, but does not sufficiently discuss how advanced adaptive algorithms may exacerbate or mitigate inequalities (e.g., data sparsity for low SEP groups, bias, explainability), nor how evaluation strategies should explicitly address these issues. | Thank you, we elaborated further on how to consider the algorithmic implications for the low SEP group and added more examples to our previous version. We also highlighted these considerations within the conceptual framework for increased clarity.  **The following is now in the manuscript:**  *“However, it is important to consider that data from vulnerable populations are frequently underrepresented or missing from the datasets used to train AI-based models, which can further augment existing societal biases [108]. To address this issue, first, co-design and community-based participatory research should be leveraged for intervention design, with community members being involved as a part of the research team. This will ensure that the developed AI tools are bias-free and sufficiently adapted to the needs and preferences of the target group. Next, the data that is used for AI model training should first be evaluated for representativeness of the target group and for any existing biases. When limited or no training data is available, additional data collection methods should serve the purpose of (re-)training models in subsequent trials or trial iterations. Using wearables is especially important, to include the low-SEP group in the data infosphere, from which they are often missing due to the lack of real-world use of digital devices [109]. Lastly, the performance and explainability of the developed model(s) must be evaluated on the target sub-group data to ensure sufficient transparency. Subsequently, computational evaluations should be complemented with community/user evaluations to validate the system’s performance and fairness in its tailored output.”* | p.32  lines 553-566 |
| 2.4 | - The manuscript is at parts too general - for example, it does not specify what kinds of adaptive algorithms (e.g., reinforcement learning, contextual bandits, dynamic Bayesian models, LLM-based dialog systems) are realistically applicable in this domain, under what data conditions, and with what constraints (e.g., fairness, transparency, resource requirements). | Thank you. We previously described the use of reinforcement learning, as well as LLM-based algorithms. We extended this section to include considerations regarding the potential constraints when using adaptive algorithms. We also added pointers for the type of algorithms that could be applicable in this domain. Lastly, we elaborated the future work section of the section specifically discussing LLM-based tailoring, and added considerations regarding LLM’s constraints.  **The following is now in the manuscript:**  *“For the algorithms executing the tailoring, we found that most interventions seemed to rely on rule-based algorithms. Such an approach remains prevalent in the existing eHealth literature, as also shown in other reviews on just-in-time adaptive interventions [101] or digital mental health interventions [102] targeting the general population. Rule-based methods can circumvent training data requirements of other methods, more easily incorporate clinical guidelines [103,104], and provide more straightforward and transparent decision-making. However, they still pose challenges, such as rule construction, which can become complex when adapting to multiple variables, or variables of idiosyncratic nature (e.g., user preferences). For more granular and dynamic tailoring, artificial intelligence (AI)-based algorithms provide greater flexibility. AI-based methods, leveraging, e.g., RL, can incorporate many tailoring variables simultaneously and extract data patterns to continuously adapt to the user’s actions or circumstances [105,106]. This is also evident in our results where one intervention used RL with almost all tailoring variable types to jointly tailor content and timing [62]. This means that interventions can draw on more diverse data types and sources (e.g., behavioral, contextual, feedback, preferences) to extend their tailoring capabilities and limit additional manual development of rule-based conditions. Similarly, adaptive recommender systems could curate content (themes) within the intervention, which was one of the largest areas for low-SEP-related tailoring. Using information, such as health status, barriers, cultural background, or self-efficacy to adapt the content selection, also if changes occur over time, could help to remove friction and address specific needs of each user. Since these methods require training data, which are not always available or abundant, models that can work with smaller amounts of data or that can be trained dynamically on data collected during the intervention may be preferred in the eHealth context. These can also be embedded within rule-based systems to support simpler tailoring with straightforward decision-making (e.g., evaluating whether a goal has been reached) complemented with more adaptive, data-driven tailoring where possible (e.g., adaptive goal-setting). Future work should explore this gap further to delineate the possibilities and requirements for implementing adaptive algorithms within this context.*  *The recent paradigm shift in the competence of large language models (LLMs), such as ChatGPT, has enabled automatic content generation, widening the scope of possibilities for tailoring even further. For example, creating content that is directly tailored to an individual can be challenging, but LLMs can leverage user-specific information to generate fully individualized content. This includes real-time generation of text and/or images, supporting functions of feedback selection, variable substitution, or recommendation provision [107]. LLMs could facilitate low-SEP-specific tailoring through e.g., user profiles or interactive collaboration that moves away from by-design approaches. For example, adapting language complexity, length or tone of text to match language skills, or community preferences, which are important tailoring aspects for this target population. With the rapid LLM development, many pre-trained LLM models can nowadays be used off-the-shelf with some additional finetuning, reducing the resource and data requirements for their usage. To be able to implement LLMs for tailoring, further research is required to ensure their trustworthiness in the eHealth context. Future work should focus on delineating appropriate LLM guardrails that mitigate inaccurate information and ensure that it remains transparent and within approved medical guidelines. With such insights, this area of research holds a promising direction for more sophisticated tailoring methods, especially for low-SEP-relevant adaptations.”* | p. 31-32  lines 511-552 |
| 2.5 | - The call for “rigorous evaluation methods” remains vague. There is no concrete discussion of study designs or analytic strategies capable of isolating the effect of tailoring (e.g., factorial designs, SMART trials, micro-randomized trials, A/B testing of tailoring vs non-tailoring, mediation analyses to link tailoring mechanisms to outcomes). | Thank you. In our discussion section we had discussed the potential approaches towards evaluation of tailoring, including the suggested examples of evaluation methods. We added some more examples to this section, as well as to the conclusion section. We also incorporated the examples of evaluation strategies into the framework to increase clarity. Lastly, we also added more elaboration to the author summary for further clarification.  **The following is now in the manuscript, discussion section:**  *“While qualitative judgments can provide valuable insights into users’ perceptions, comparative evaluations can additionally aid in separating the effects of tailoring mechanisms from the effects of the intervention as a whole. For example, to isolate the effects of tailoring, the comparator should not be a fundamentally different intervention. Oftentimes, tailored interventions are compared against routine care, which obscures the exact value that tailoring contributes to the overall effectiveness of the new intervention. Rather, the tailored intervention should also be compared against a non-tailored counterpart, next to routine care. In this way, a baseline effect can be determined of the new approach over established procedures, as well as what added effects the tailoring provides. Thus, future research should focus on evaluating tailoring approaches, for example via micro-randomized trials [114], by comparing the tailored system to a non-tailored one with e.g., a factorial trial design, or A/B testing of tailored variants. An important metric to be included in such evaluations is engagement. Engagement may improve intervention effectiveness, thus insights into effective tailoring approaches, as well as potential engagement barriers that could be solved with tailoring are crucial to evaluate and report. Together with more comprehensive reporting on the used tailoring algorithms, these steps can ensure reproducibility and facilitate stronger analyses of the outcomes of different tailoring methods.”*  **The following is now in the manuscript, conclusion section:**  “*Future research should explore the integration of adaptive algorithms to improve the tailoring process. However, this must be accompanied by rigorous evaluation methods and trial designs, such as factorial design, that can differentiate the specific impact of tailoring from the overall effectiveness of eHealth interventions*”  **The following is now in the manuscript, author summary section:**  *“Future research should further investigate the use of adaptive algorithms for tailoring and prioritize evaluation trial designs that can differentiate the impact of different tailoring methods.”* | Discussion section:  p. 33-34  lines 574-589  Conclusion:  p. 36  lines 647-650  Author summary:  p.3  lines 59-61 |
| 2.6 | - The “future research” section reads more like a generic wish list than a substantive, actionable research agenda. For a journal like PLOS ONE, where the bar is methodological soundness and clearly supported conclusions, this lack of elaboration undermines the manuscript’s added value for the field. | Thank you. Our manuscript does not contain a single “future work” section, but rather, for each discussed topic in the Discussion section, the future work possibilities are delineated at the end of each paragraph. We have restructured some of the future work suggestions, to create a more concrete vision/guidelines for future work in eHealth tailoring. Moreover, with the addition of the conceptual framework that directly highlights the gaps/underexplored areas, the research agenda for future work stands out more clearly. | p. 30, lines 483-487  p. 31, lines 506-510  p. 32, lines 534-536  p. 32-33, lines 547-552  p. 33-34, lines 582-589 |
|  | Reviewer #4 comments | Response to Reviewer #4 | Manuscript page and line numbers |
| 4.1 | I recommend to mention the relation of your study with Sustainable Development goals, this papers is involved directly/indirect with most of the SDG Goals, a section explaining the relation with SDG will be very good. | Thank you, we included this as part of the Strengths & Limitations section.  **The following is now in the manuscript:**  “*Moreover, our review contributes to the Sustainable Development Goals (SDGs) by investigating and promoting appropriate tailoring approaches within eHealth lifestyle interventions. We contribute to the SDGs #3 “Good health and well-being” (target 3.4) and #10 “Reduced inequalities” (targets 10.2) by bringing awareness to the existing tailoring approaches and where improvements can be made. Our work can be used to increase the effectiveness and SEP-related relevance of eHealth interventions, promoting reduction of non-communicable disease-related mortality (SDG #3) and inclusion and participation of low SEP groups (SGD #10)*.” | p. 34  lines 596-602 |
| 4.2 | Furthermore, I accept this manuscript. Please refer to other reviewers with solid background in statistics analysis, I should highlight that i am /specialist in GIS/health with spatial analysis. | Thank you |  |
|  | Reviewer #5 comments | Response to Reviewer #5 | Manuscript page and line numbers |
| 5.1 | The authors provide an important contribution to the literature by synthesizing tailoring techniques in eHealth lifestyle interventions for people with cardiometabolic conditions and lower socioeconomic position (SEP). This scoping review addresses a critical gap: while tailoring is frequently cited as essential for improving interventions targeting individuals with low SEP and chronic conditions, clear guidance on what to tailor and how to implement tailoring remains lacking. This comprehensive review demonstrates thoughtful and systematic consideration of tailoring variables in prior studies and approaches through elaborate thematic clustering. The manuscript may benefit from several clarifications and typographical corrections.  Strengths:  - The introduction effectively establishes the absence of synthesized knowledge and guidelines for tailoring eHealth interventions to low-SEP populations and provides comprehensive mapping of existing evidence to address this gap. The manuscript language is clear, easy to read, and concise (aside from the minor typographical errors noted below).  - The review appropriately follows Arksey and O'Malley framework and PRISMA-ScR guidelines. The use of both inductive and deductive methods for thematic clustering and coding of tailoring areas and levels was appropriate for the scope of this review. Double screening strengthens the reliability of study selection. Supplemental links work properly and materials are easy to access and review.  - The authors acknowledge important limitations, including that "evaluation of tailoring was missing from most studies (n=15/27, 55%)" while highlighting the importance of sociocultural, language, health literacy-sensitive design factors.  - The revision letter thoroughly addressed concerns raised by all prior three reviewers. The authors made reasonable changes where possible and appropriately justified decisions when changes weren't feasible (e.g., explaining why tailoring-specific effectiveness analyses weren't included: "most interventions did not evaluate the effects of tailoring specifically, outside the effects of the intervention"). | Thank you |  |
| 5.2 | Abstract: 1. Line 43, page 2: "SEP" abbreviation is used without first defining it. Please spell out "socioeconomic position (SEP)" on first use. Introduction | Thank you, we added the full form.  *“…and low socioeconomic position (SEP).”* | p. 2  line 31 |
| 5.3 | 2. Line 97, page 5: This sentence “Otherwise, the potential for increased usability [30,31], improved adherence [30], and better health outcomes [32,33] is disregarded.” Is awkwardly phrased. Do you mean will “not be realized” or “…interventions miss the opportunity for…”? | Thank you, we reformulated the sentence:  *“Otherwise, interventions miss the opportunity to improve usability [30,31], adherence [30], and health outcomes [32,33].”* | p.5  lines 99-100 |
| 5.4 | Methods: 3. Line 132, page 6: Consider writing out the PICO framework with a brief sentence explaining its use, similar to what was done for the PCC framework. | Thank you, we added more clarification:  *“We structured the eligibility criteria (see* ***Textbox 1****) using relevant components of the PICO (Population, Intervention, Comparison, Outcome) framework [39]. Each PICO component was defined according to our review’s aims (e.g., requiring an intervention description), as well as the key concepts of the search string. Due to the exploratory nature of our review, we did not apply the framework’s criteria regarding comparisons or outcomes.”* | p.6  lines 125-129 |
| 5.5 | Methods: 4. Line 294, page 20: I believe the word BCT is an abbreviation that needs to be written out. I do not see it used elsewhere in the paper. | Thank you. Full form of the BCTs abbreviation were added to the list of abbreviations under the table. | p. 21  Table 6 |
| 5.6 | Results/Discussion: 5. Line 362, page 24: Remove double periods in ". . The number" | Thank you, double period removed. | p. 24  line 369 |
| 5.7 | Results/Discussion: 6. Line 489, page 30: Clarify "This means interventions can draw on more data" - do you mean more data types/sources? Consider: "This means interventions can draw on more diverse data types and sources (e.g., behavioral, contextual, feedback, preferences)" | Thank you, we added the clarification:  *“This means interventions can draw on more diverse data types and sources (e.g., behavioral, contextual, feedback, preferences),”* | p. 31  line 523 |
| 5.8 | Results/Discussion: 7. Line 518, page 31: Change "as also shown in our results" to "as shown in our prior work" to minimize confusion | Thank you, we clarified this part:  “*Although the results of this review, as well as existing literature, show [..]*” | p. 33  line 570 |
| 5.9 | Results/Discussion: 8. Line 548, page 30: Remove extra spacing in "es . Such..." | Thank you, space removed. | p. 34  line 605 |
| 5.10 | Results/Discussion: 9. Results/RQ3 line 103, Page 6: I appreciate that RQ3 appropriately focuses on evaluation methods and phases, which aligns well with the scoping review methodology. Given that the authors establish improved adherence as a key potential benefit of tailoring in the abstract, author summary, and introduction, I wonder whether summary reporting of adherence and retention outcomes (where available) might add practical value to the findings. While I recognize that most studies (n=15/27, 55%) did not evaluate tailoring effectiveness specifically, I note that the authors mention adherence in the context of Progress calculation (n=10 studies). This suggests that at least some studies reported engagement metrics.  Questions that come to mind are:  1) Were adherence, retention, or completion rates reported descriptively in additional studies beyond the 10 mentioned?  2) If so, would a summary table of these outcomes across studies enhance the practical applicability of the findings for researchers designing interventions for low-SEP populations?  3) If this data was inconsistently reported across studies, might this gap be worth highlighting as a recommendation for future research reporting standards? Even without being able to attribute outcomes to tailoring specifically, descriptive data on intervention engagement in this population could provide valuable context and strengthen alignment with the adherence-related rationale presented in the introduction. | Thank you. We analyzed the results of the included intervention papers for engagement metrics and results (with or without links to tailoring). We have included this data into the S4 Table 1, next to data on evaluation phases. We then summarized these findings in the results section, to highlight any effects of tailoring on engagement or of engagement on intervention effectiveness. This creates more concrete guiding points for future interventions on which intervention elements might be of added value, as well as where tailoring could be used to tackle engagement barriers.  **The following is now in the manuscript, results section:**  *“Several (n=5/27, 19%) of the interventions that evaluated tailoring also reported effects regarding engagement [64,71,75,77,85] (see* ***S4 Table 1****). One study reported an overall high engagement, which led to improved diabetes knowledge and higher confidence in performing diabetes self-care activities [64]. Two studies specifically highlighted the use of (audio-)visual materials [71] and health literacy-sensitive design [77] as having a positive effect on engagement. One study reported moderate engagement rates, with low usage of goal-setting tools [85]. Lastly, in one study, the lack of tailoring of the informational content to the participants’ pre-existing knowledge had a negative impact on engagement [75].*  *Eight other interventions (n=8/27, 30%) [48,51,54,57,67,72,73,87], which did not evaluate tailoring, reported overall high engagement rates, which led to increased weight loss in two [54,57]. One study specifically found a link between higher engagement rates with the self-tracking tools and an improvement in eating habits and physical activity levels [72]. SMS text messages were also found to produce higher engagement rates over printed [51], as well as voice messages [67] where participants faced challenges, such as not understanding the instructions, the spoken language, or not being given enough time to respond to the voice message. Lastly, one study reported decreasing engagement due to participants losing interest in the provided content [48], and one study remained inconclusive on role of high engagement metrics in improving biological outcomes [73].”*  While most studies (where this would be appropriate) reported the engagement metrics, we still highlighted the value of reporting on this metric also in future evaluations:  **The following is now in the manuscript, discussion section:**  “*Regarding the evaluation, we found positive effects of tailoring on user perceptions, engagement and intervention outcomes.*  *[..]*  *An important metric to be included in such evaluations is engagement. Engagement may improve intervention effectiveness, thus insights into effective tailoring approaches, as well as potential engagement barriers that could be solved with tailoring are crucial to evaluate and report.*” | Results section:  p. 28  lines 430-446  Discussion section:  p. 33  lines 567-568,  p. 34  lines 585-587 |
| 5.11 | 10. Line 504, page 31: The authors provide an excellent overview of large language models (LLMs) in digital health and appropriately note that “community preferences are important tailoring aspects for this target population.” To strengthen this section, I recommend the authors explicitly discuss the importance of community-based participatory research (CBPR) and stakeholder engagement in developing LLM-based interventions. Given the documented digital divide and historical exclusion of low-SEP populations from technology development, participatory approaches are essential to ensure these tools are culturally appropriate, address community-identified needs, and avoid perpetuating existing health inequities. Additionally, the authors should note that community members should be meaningfully compensated as experts and co-developers, not simply as research participants. Citation [78] may be a good reference for this. | Thank you, we included this topic in the section discussing vulnerable populations and data for AI-based models (see point 2.3). | p. 32-33  lines 553-557 |
