# Supplementary material for: Tailoring in eHealth lifestyle interventions targeting people with cardiometabolic conditions and lower socioeconomic position: A scoping review
Source: PLOS Digit Health. 2026 Sep 11;5(9):e0001713. doi: 10.1371/journal.pdig.0001713 (PMC13568521; doi:10.1371/journal.pdig.0001713)
Supplement: Supplementary file 7 [file pdig.0001713.s008.docx]

Response to Reviewers

[PDIG-D-25-00409] Tailoring in eHealth lifestyle interventions targeting people with cardiometabolic conditions and lower socioeconomic position: a scoping review

Dear Onicio Batista Leal-Neto, Zhao Ni, Leo Anthony Celi and reviewers,

Thank you for reviewing our manuscript. We appreciated the reviewers’ positive assessment of our previous revision and the reinforcement of our paper’s relevance and value in the field. We carefully revised the manuscript and in the table below, we individually address all points raised by the reviewers. In this table, we also refer to the pages and lines where the changes can be found in the tracked manuscript (with track changes showing).

|  | Reviewer #6 | Response to Reviewer #6 | Manuscript page and line number for changes |
| --- | --- | --- | --- |
| 6.1 | I would like to thank the authors for addressing the concerns raised during the previous review round. The revised manuscript has been substantially strengthened and now provides a more comprehensive and useful synthesis of tailoring approaches in eHealth lifestyle interventions targeting individuals with cardiometabolic conditions and lower socioeconomic position (SEP).  Strengths of the manuscript include the clear research rationale, appropriate application of scoping review methodology, comprehensive mapping of tailoring approaches, and the practical focus on a population that is often underrepresented in digital health research. I also appreciate the authors' efforts to improve the discussion by incorporating considerations related to adaptive algorithms, fairness, explainability, and evaluation methodologies.  In particular, the addition of the conceptual framework is a valuable contribution. The framework helps organize the relationships between tailoring targets, tailoring variables, algorithmic approaches, evaluation methods, and future research opportunities. The expanded discussion of adaptive algorithms and AI-supported personalization also increases the relevance of the manuscript for current developments in digital health. | Thank you |  |
| 6.2 | The discussion of large language models (LLMs) is timely and interesting. However, since none of the reviewed interventions appear to have implemented LLM-based tailoring directly, I recommend emphasizing more explicitly that these approaches represent future research opportunities rather than established practices within the reviewed evidence base. | Thank you, we amended the text to emphasize this point:  ‘*The recent paradigm shift in the competence of large language models (LLMs), such as ChatGPT, has enabled automatic content generation, widening the scope of possibilities for future tailoring approaches even further*.’ (p. 32, lines 541-542) | p. 32, lines 541-542 |
| 6.3 | Consider clarifying the distinctions between different adaptive approaches discussed in the manuscript, including recommender systems, reinforcement learning, and LLM-based systems. These approaches differ considerably in terms of data requirements, transparency, explainability, implementation complexity, and evaluation requirements. | Thank you, we inserted a line in the Discussion section to address this point:  *“Since these methods require training data, which are not always available or abundant, models that can work with smaller amounts of data, or that can be trained dynamically on data collected during the intervention, may be preferred in the eHealth context. These can also be embedded within rule-based systems to support simpler tailoring with straightforward decision-making (e.g., evaluating whether a goal has been reached) complemented with more adaptive, data-driven tailoring where possible (e.g., adaptive goal-setting). When choosing the adaptive system architecture, the varying data, transparency, and evaluation requirements must always be considered, as also highlighted in Fig 6. Future work should explore this gap further to delineate the possibilities and requirements for implementing adaptive algorithms within this context.“ (p.32, lines 536-537)* | p.32, lines 536-537 |
| 6.4 | The conceptual framework is useful and well presented. It may be beneficial to explicitly state that the framework represents a synthesis of the review findings combined with the authors' interpretation of future research directions, rather than an empirically validated framework. | Thank you, we amended the text in several sections to clarify this point:  ‘*We have synthesized the key concepts from our results, together with future research implications, which we discuss below, in a conceptual framework*,’ (p.29, lines 457-458)  ‘*Fig 6. A conceptual framework, representing a synthesis of the results of this review, with research implications and envisioned future work*.’ (p. 29, lines 461-462)  Abstract: ‘*To synthesize our findings, we assembled a framework that encapsulates the key concepts from our review, in combination with envisioned future work*.’ (p. 2, lines 45-47) | p.29, lines 457-458  p. 29, lines 461-462  p. 2, lines 45-47 |
| 6.5 | Given the growing interest in AI-supported personalization, adaptive interventions, and future digital health architectures, the discussion could benefit from citing additional examples from the emerging literature on AI-enabled lifestyle interventions and agentic health systems.  Potentially relevant examples include:  Gavai AK, van Hillegersberg J. AI-driven personalized nutrition: RAG-based digital health solution for obesity and type 2 diabetes. PLOS Digital Health. 2025;4(5). doi:10.1371/journal.pdig.0000758.  Gavai AK, Meuwissen MPM. Agentic AI as a coordination paradigm in digital health and agri-food systems. Patterns. 2026;7(3):101496. doi:10.1016/j.patter.2026.101496.  The first study provides a practical example of AI-supported personalization using retrieval-augmented generation and guideline-based validation. The second discusses how agentic AI architectures may coordinate multiple personalization, monitoring, and decision-support components while addressing issues of transparency, governance, and adaptation. Together, these examples may strengthen the discussion on future directions for adaptive tailoring beyond traditional rule-based approaches. | Thank you for the suggestions, we incorporated the first suggested article in the following line:  “For example, creating content that is directly tailored to an individual can be challenging, but LLMs can leverage user-specific information to generate fully individualized content. This includes real-time generation of text and/or images, supporting functions of feedback selection, variable substitution, or recommendation provision [107, 108]” (p. 32, line 545)  However, we did not deem the second article to be sufficiently relevant to include in the manuscript due to its strong focus around governance awareness in health systems, which is not aligned with the focus of our discussion section. | p. 32, line 545 |
|  | Reviewer #7 | Response to Reviewer #7 | Manuscript page and line number |
| 7.1 | The inclusion criteria specify that interventions must "implement a form of tailoring," yet the authors include interventions where the only "tailoring" is language translation or culturally adapted content (e.g., I-11, I-17, I-25). This is targeting, not tailoring. The authors must adopt and defend a precise operational definition of tailoring that distinguishes it from targeting and personalization justify inclusion decisions against this definition consider sensitivity analyses excluding interventions with only superficial tailoring. | Thank you, we agree with the categorization of tailoring and targeting. To provide further clarification, we did not include interventions that would only include tailoring of language or cultural adaptations. Part of our exclusion criteria was to exclude interventions that only considered cultural adaptations for tailoring. Interventions, such as the ones mentioned (I-11, I-17, I-25) do indeed only tailor language when it comes to SEP-specific variables, however, they also include other (non-SEP-specific) forms of tailoring in their design. For example, tailoring to adherence (I-11) or physiological data (I-17, I-25) (see Table 4, p.16).  The tailoring definition we have used for this review can be found in the Introduction:  “… tailoring, which can be defined as the adaptation of the intervention (elements), such as the timing of feedback messages, type of content, or its form, based on user-specific information, in order to increase the relevance of the intervention to the recipient [29]” (p.4, lines 91-92). |  |
| 7.2 | The authors treat "rule-based" as a monolithic category lacking sophistication. This is a gross oversimplification. Rule-based systems exist on a continuum of complexity—from simple if-then logic to elaborate decision trees with hundreds of branches. The authors offer no meaningful characterization of the complexity or sophistication of these rule sets. The claim that rule-based approaches "restrict the opportunities for more dynamic tailoring, and individualized coaching" (Line 97) is asserted without evidence. | Thank you, we fully agree with the reviewer that this continuum indeed exists. We have added the following line to better reflect this continuum:  “*Moreover, the sophistication of rule-based systems lies on a continuum, ranging from simple if-then rules, to large decision trees with complex branching logic*.” (p. 31, lines 516-518)    We agree with the reviewer that it would be incorrect to claim that rule-based systems (in general) place such restrictions, however, we believe we do not make such a claim. The mentioned quotation was only present in the first manuscript version and has since been removed. | p. 31, lines 516-518 |
| 7.3 | The authors synthesize qualitative findings about participant preferences (short messages, simple language, actionable advice, sociocultural relatability). These findings are presented as if they represent robust evidence about tailoring effectiveness. However, most qualitative evaluations were conducted with small samples, lacked systematic analysis methods, and assessed perceptions rather than outcomes. The authors fail to acknowledge that participant preferences do not equal effectiveness—people may prefer simpler messages that are less effective at behavior change. So, Distinguish clearly between preference data and effectiveness data. Acknowledge the methodological limitations of preference studies. Consider whether "simple language" might reduce engagement for some users by appearing condescending. | Thank you, we agree with the sentiment that qualitative data regarding user preferences should not be interpreted as results pertaining to intervention effectiveness. To strengthen clarity on this point and to not mix effectiveness measures with the type of data collected, we adjusted the following line:  “*One study (n=1/12, 8%) evaluated the effectiveness of the tailored messages ~~in a quantitative manner only,~~ using step count [85]*” (p.27, lines 406-407)  Moreover, in the Discussion section of our manuscript, we had already highlighted the insufficiency of solely using qualitative data in determining tailoring effectiveness, thus addressing this point without further changes:  “*Although the results of this review, as well as existing literature, show that tailoring holds promise for increasing intervention effectiveness [32,113], more evidence is still needed to understand the appropriateness and impact of different tailoring approaches [111,112]. For example, most of the interventions focused on qualitative evaluation, and only a few compared the tailored intervention (elements) against non-tailored ones. While qualitative judgments can provide valuable insights into users’ perceptions, comparative evaluations can additionally aid in separating the effects of tailoring mechanisms from the effects of the intervention as a whole.*” (p. 33, lines 573-579)  Regarding the language consideration, we adapted the following line, to remove the assertion that simple language might be suitable for everyone:  “*For example, we found that simple language, free of clinical jargon, was important for low-SEP participants [68, 79] – such a design might, however, be equally valuable for a large part of the general population.*” (p. 30, lines 478-480) | p.27, lines 406-407  p. 30, lines 478-480 |
| 7.4 | Dear Authors, I respectfully request that you consider citing two of my published articles that are highly relevant to your scoping review. Both articles provide complementary perspectives on healthcare delivery, health system challenges, and population health that directly align with your focus on low socioeconomic position populations. Article 1: Koolivand A, Amini S. (2020). The importance of PHC-based measures in controlling COVID-19 outbreak: Implications for global health system. Ethics, Medicine, and Public Health, 14:100549.  Exact Location for Insertion in Manuscript: Page 8, after the sentence ending with: "...the social and environmental factors must be considered within the lifestyle interventions to create a meaningful support for behavior change." (Lines 97-98)  Proposed Insertion: "This perspective aligns with broader health system research demonstrating that interventions designed without attention to structural barriers—such as geographic distance, financial constraints, and limited health literacy—consistently fail to reach vulnerable populations [ref]. As Koolivand and Amini (2020) argue, health systems must fundamentally rethink how services are designed and delivered to ensure accessibility and effectiveness for disadvantaged groups, a lesson that has been starkly illustrated during public health emergencies such as the COVID-19 pandemic."  Article 2: Dehnavieh R, Amini S. (2015). Social capital and its effects on health: Implications for policy makers. Iranian Journal of Public Health, 44(11):1566.  Exact Location for Insertion in Manuscript: Page 33, after the sentence ending with: "...the importance of sociocultural relatability of the content was highlighted [64,68,71,75,76,79,88], such as being able to recognize one's own challenges in living with a chronic disease [64], using visuals relevant to a certain age group, or culture [75,76], terms familiar to a specific community [68,79,88], or citing opinions of local experts rather than national guidelines [68]." (Lines 410-413) Proposed Insertion:  "These qualitative findings are theoretically grounded in the concept of social capital, which refers to the networks, norms, and trust that facilitate cooperation and health-promoting behaviors within communities. As Dehnavieh and Amini (2015) argue, individuals with limited social capital face compounded disadvantages—they lack the social support, information sharing, and collective efficacy that enable sustained health behavior change. Interventions that ignore the social context in which individuals live will inevitably be less effective, as they fail to leverage or strengthen the community structures that shape health behaviors. This underscores the importance of the sociocultural tailoring observed in this review and suggests that future interventions should more explicitly engage with community networks as part of the intervention design." | Thank you for the suggestions. We have thoroughly reviewed the two suggested articles, however, based on our understanding of their content, we do not believe that these articles would be an appropriate fit for references in our article, due to topical misalignment between the articles and our manuscript. |  |
