# Supplementary material for: Tailoring in eHealth lifestyle interventions targeting people with cardiometabolic conditions and lower socioeconomic position: A scoping review
Source: PLOS Digit Health. 2026 Sep 11;5(9):e0001713. doi: 10.1371/journal.pdig.0001713 (PMC13568521; doi:10.1371/journal.pdig.0001713)
Supplement: Peer review history

# Peer review: Decision Letter 0

**DOI:** 10.1371/journal.pdig.0001713.r001

**Authors:** Aline Lutz de Araujo, Onicio Batista Leal-Neto

21 Aug 2025

Response to Reviewers Revised Manuscript with Track Changes Manuscript **Journal Requirements:**

1. Please amend your detailed Financial Disclosure statement. This is published with the article. It must therefore be completed in full sentences and contain the exact wording you wish to be published.

i. State the initials, alongside each funding source, of each author to receive each grant.

2. We ask that a manuscript source file is provided at Revision. Please upload your manuscript file as a .doc, .docx, .rtf or .tex.

3. Please upload separate figure files in .tif or .eps format. Also, remove the figures from your manuscript file but keep the legends.

For more information about figure files please see our guidelines:

https://journals.plos.org/digitalhealth/s/figures

https://journals.plos.org/digitalhealth/s/figures#loc-file-requirements

**Additional Editor Comments (if provided):**;**Reviewers' Comments:**

**Comments to the Author**

1. Does this manuscript meet PLOS Digital Health’s publication criteria?

Reviewer #1: Yes

Reviewer #2: Partly

Reviewer #3: Partly

**********

2. Has the statistical analysis been performed appropriately and rigorously?

Reviewer #1: Yes

Reviewer #2: No

Reviewer #3: Yes

**********

3. Have the authors made all data underlying the findings in their manuscript fully available (please refer to the Data Availability Statement at the start of the manuscript PDF file)?

The PLOS Data policy

Reviewer #1: Yes

Reviewer #2: Yes

Reviewer #3: Yes

**********

4. Is the manuscript presented in an intelligible fashion and written in standard English?

Reviewer #1: Yes

Reviewer #2: Yes

Reviewer #3: Yes

**********

Reviewer #1: your review provides synthesis of tailoring approaches used in eHealth interventions targeting low-

542 SEP populations with cardiometabolic conditions. It also findings also uncover several gaps and opportunities for further research for eHealth lifestyle interventions in lower socioeconomic population having cardiometabolic conditions.

Reviewer #2: Overall Evaluation

This manuscript presents a comprehensive scoping review examining tailoring approaches within eHealth lifestyle interventions for individuals with cardiometabolic conditions and lower socioeconomic positions (SEP). The paper is well-written, methodologically sound, and addresses a relevant public health issue. It maps the current landscape of tailoring strategies in eHealth interventions, emphasizing gaps and future research directions.

________________________________________

Strengths

1. Relevance and Timeliness

o The focus on tailoring eHealth interventions for lower SEP populations is highly relevant, considering the health disparities and digital divide in chronic disease management.

o Cardiometabolic conditions are a critical global health burden, making the topic of this review particularly impactful.

2. Comprehensive Methodology (should be improved)

o The scoping review follows the Arksey and O’Malley framework and PRISMA-ScR checklist, demonstrating rigor and transparency. Yet, many papers are pre GenAI epoch and therefore their eHealth tailoring approach and recommendations could be extrapolated to better reflect the abilities nowadays.

o A broad search strategy across multiple databases (Scopus, Web of Science, PubMed, PsycINFO) ensures thorough coverage of relevant literature (I am not sure if all the data is available to the readers – please indicate clearly)

3. Contributions (should be improved – see below)

o The paper identifies gaps in existing interventions (e.g., overreliance on rule-based algorithms - that are quite old and limited evaluation of tailoring).

o It provides actionable insights for researchers, including the need for dynamic, adaptive tailoring using computational algorithms.

4. Clarity of Presentation

o The manuscript is logically structured, with detailed tables and figures summarizing interventions (quality of some figures should be improved!), tailoring variables, and algorithm types.

o The discussion connects findings to practical implications and future research needs, yet should be further extended.

5. Innovation Potential (should be improved – see below)

o By highlighting the underutilization of AI-based or adaptive tailoring techniques, the paper sets a foundation for innovative digital health solutions.

________________________________________

Weaknesses

1. Limited Critical Analysis of Intervention Quality

o While the paper maps tailoring approaches, there is little discussion about the quality or effectiveness of the interventions, beyond descriptive reporting.

o More critical analysis of the success (or failure) of specific tailoring strategies would strengthen the contribution.

2. Innovation Gap in Findings

o Although the paper is comprehensive, the review largely synthesizes existing literature without proposing a conceptual framework or new model for tailoring. This is particularly true when considering that many of the reviewed works are pre GenAI epoch, and that the possibility to implement new approaches – granular and personal – are much higher nowadays vs. 10 years ago! For example, mentioning two papers from 2015 and 2019 [68,79], i.e. pre GenAI, that propose "straightforward language with layman terms and positive framing" can be accompanied by new possibilities of AI theses days.

o The automated procedure for screening and selecting relevant papers is systematic and thorough, however the outcome of extracting information from those papers can be elevated. The integration of relevant modern approaches to generate new ideas and policies is required.

o Following the above, a more explicit discussion of how emerging technologies (e.g., GenAI, personalization engines etc) could be integrated might add novelty.

3. Overemphasis on Descriptive Statistics

o The results section is heavily descriptive (e.g., counts of studies), which sometimes overshadows interpretative insights. A stronger narrative synthesis, including potential effectiveness of the recommendation, could improve readability and impact.

4. Evaluation of Tailoring

o The paper highlights that over half of interventions lacked evaluation of tailoring, but it does not provide in-depth recommendations or criteria for how evaluation should be conducted.

5. Minor Scope Limitation

o Many of the papers focus on diet and physical activity recommendations - this should be reflected in the abstract and conclusions – thus, narrowing the claimed generality. The exclusion of certain behavioural domains (e.g., mental health or smoking cessation) is understandable but may limit the generalizability of conclusions about tailoring methods.

________________________________________

Recommendation

Recommendation: Major Revisions Before Acceptance

The manuscript makes a relevant contribution to the field of digital health by systematically mapping tailoring approaches for vulnerable populations. With some improvements in critical analysis, the addition of a conceptual framework or more future-oriented recommendations, and a slightly less descriptive tone, the paper could be an excellent addition to PLOS Digital Health.

Reviewer #3: Strengths:

Clear scope focusing on low-SEP populations; avoids race/ethnicity as a sole SEP proxy.

Transparent and updated search methodology.

Effective conceptual mapping of tailoring and algorithmic functions; prevents double-counting by merging multi-paper records.

Acknowledges the frequent lack of tailoring evaluation in literature.

Suggestions for Revision:

Dual Screening and Coding: Expand QA beyond 20% title/abstract double-screening. Conduct and report independent duplicate full-text screening and coding for a robust subset (e.g., ≥25–30%), including κ statistics and resolution procedures.

Algorithm Classification Transparency: Explicitly flag inferred algorithm types/functions in tables/figures with textual evidence or "not reported." Consider a "Reported by authors" vs. "Inferred by reviewers" column.

Data Availability: Deposit the complete study-level extraction spreadsheet and full list of excluded full-text records with reasons in an open repository (DOI preferred).

Denominators and Sensitivity Analyses: Report counts and denominators for evaluation practices/algorithm types. Consider sensitivity analysis excluding interventions without deployed evaluation.

Limitations: Discuss potential selection bias from including protocols/design papers and temper language in the Abstract/Conclusion accordingly.

Minor Suggestions:

State whether full-text screening and data extraction involved independent duplication and where details are located.

Ensure consistent use of n=27 interventions vs n=43 papers across all percentages.

Consider a figure to visually separate by-design vs in-intervention dynamic tailoring.

**********

what does this mean?). If published, this will include your full peer review and any attached files.

**Do you want your identity to be public for this peer review?**  If you choose “no”, your identity will remain anonymous but your review may still be made public.

For information about this choice, including consent withdrawal, please see our Privacy Policy

Reviewer #1: No

Reviewer #2: No

Reviewer #3: No

**********

**Figure resubmission:**

**Reproducibility:**

To enhance the reproducibility of your results, we recommend that authors of applicable studies deposit laboratory protocols in protocols.io, where a protocol can be assigned its own identifier (DOI) such that it can be cited independently in the future. Additionally, PLOS ONE offers an option to publish peer-reviewed clinical study protocols. Read more information on sharing protocols at https://plos.org/protocols?utm_medium=editorial-email&utm_source=authorletters&utm_campaign=protocols

# Author comment: Author response to Decision Letter 1

**DOI:** 10.1371/journal.pdig.0001713.r002

20 Oct 2025

Attachment Submitted filename: Response to Reviewers.docx

# Peer review: Decision Letter 1

**DOI:** 10.1371/journal.pdig.0001713.r003

**Authors:** Zhao Ni, Onicio Batista Leal-Neto

19 Mar 2026

Response to ReviewersRevised Manuscript with Track ChangesManuscript**Journal Requirements:** **Additional Editor Comments (if provided):** **Reviewers' Comments:**

**Comments to the Author**

Reviewer #2: (No Response)

Reviewer #4: All comments have been addressed

Reviewer #5: (No Response)

**********

publication criteria?

Reviewer #2: No

Reviewer #4: Yes

Reviewer #5: Yes

**********

3. Has the statistical analysis been performed appropriately and rigorously?

Reviewer #2: Yes

Reviewer #4: Yes

Reviewer #5: Yes

**********

4. Have the authors made all data underlying the findings in their manuscript fully available (please refer to the Data Availability Statement at the start of the manuscript PDF file)?

The PLOS Data policy

Reviewer #2: Yes

Reviewer #4: Yes

Reviewer #5: Yes

**********

5. Is the manuscript presented in an intelligible fashion and written in standard English?

Reviewer #2: (No Response)

Reviewer #4: Yes

Reviewer #5: Yes

**********

Reviewer #2: This review maps how tailoring is used in eHealth lifestyle interventions for adults with cardiometabolic conditions and lower socioeconomic position (SEP).

The topic is important, and the review is conducted with reasonable methodological approach. However, in its current form, the manuscript does not yet reach the level of conceptual and practical maturity expected for PLOS ONE.

Main concerns / reasons for (current) rejection

-Limited added value beyond descriptive mapping: While the descriptive overview is solid, the paper stops short of offering a conceptual framework that truly links: e.g. tailoring level → chosen algorithmic approach → required data → expected mechanisms → appropriate evaluation design. Without such structure, the significance of the review for guiding future eHealth tailoring research is reduced.

- Insufficient engagement with implications for low SEP in the algorithmic context. The manuscript notes the importance of tailoring to literacy, culture, and SEP, but does not sufficiently discuss how advanced adaptive algorithms may exacerbate or mitigate inequalities (e.g., data sparsity for low SEP groups, bias, explainability), nor how evaluation strategies should explicitly address these issues.-

- The manuscript is at parts too general - for example, it does not specify what kinds of adaptive algorithms (e.g., reinforcement learning, contextual bandits, dynamic Bayesian models, LLM-based dialog systems) are realistically applicable in this domain, under what data conditions, and with what constraints (e.g., fairness, transparency, resource requirements).

- The call for “rigorous evaluation methods” remains vague. There is no concrete discussion of study designs or analytic strategies capable of isolating the effect of tailoring (e.g., factorial designs, SMART trials, micro-randomized trials, A/B testing of tailoring vs non-tailoring, mediation analyses to link tailoring mechanisms to outcomes).

- The “future research” section reads more like a generic wish list than a substantive, actionable research agenda. For a journal like PLOS ONE, where the bar is methodological soundness and clearly supported conclusions, this lack of elaboration undermines the manuscript’s added value for the field.

Reviewer #4: I recommend to mention the relation of your study with Sustainable Development gaols, this papers is involved directly/indirect with most of the SDG Goals, a section exalaining the relation with SDG will be very good.

Furthermore, I accept this manuscript. Please refer to other reviewers with solid background in statistics analysis, I should highlight that i am /specialist in GIS/health with spatial analysis.

Reviewer #5: The authors provide an important contribution to the literature by synthesizing tailoring techniques in eHealth lifestyle interventions for people with cardiometabolic conditions and lower socioeconomic position (SEP). This scoping review addresses a critical gap: while tailoring is frequently cited as essential for improving interventions targeting individuals with low SEP and chronic conditions, clear guidance on what to tailor and how to implement tailoring remains lacking. This comprehensive review demonstrates thoughtful and systematic consideration of tailoring variables in prior studies and approaches through elaborate thematic clustering. The manuscript may benefit from several clarifications and typographical corrections.

Strengths:

- The introduction effectively establishes the absence of synthesized knowledge and guidelines for tailoring eHealth interventions to low-SEP populations and provides comprehensive mapping of existing evidence to address this gap. The manuscript language is clear, easy to read, and concise (aside from the minor typographical errors noted below).

- The review appropriately follows Arksey and O'Malley framework and PRISMA-ScR guidelines. The use of both inductive and deductive methods for thematic clustering and coding of tailoring areas and levels was appropriate for the scope of this review. Double screening strengthens the reliability of study selection. Supplemental links work properly and materials are easy to access and review.

- The authors acknowledge important limitations, including that "evaluation of tailoring was missing from most studies (n=15/27, 55%)" while highlighting the importance of sociocultural, language, health literacy-sensitive design factors.

- The revision letter thoroughly addressed concerns raised by all prior three reviewers. The authors made reasonable changes where possible and appropriately justified decisions when changes weren't feasible (e.g., explaining why tailoring-specific effectiveness analyses weren't included: "most interventions did not evaluate the effects of tailoring specifically, outside the effects of the intervention").

Suggestions:

Abstract:

1. Line 43, page 2: "SEP" abbreviation is used without first defining it. Please spell out "socioeconomic position (SEP)" on first use.

Introduction

2. Line 97, page 5: This sentence “Otherwise, the potential for increased usability [30,31], improved adherence [30], and better health outcomes [32,33] is disregarded.” Is awkwardly phrased. Do you mean will “not be realized” or “…interventions miss the opportunity for…”?

Methods:

3. Line 132, page 6: Consider writing out the PICO framework with a brief sentence explaining its use, similar to what was done for the PCC framework.

4. Line 294, page 20: I believe the word BCT is an abbreviation that needs to be written out. I do not see it used elsewhere in the paper.

Results/Discussion:

5. Line 362, page 24: Remove double periods in ". . The number"

6. Line 489, page 30: Clarify "This means interventions can draw on more data" - do you mean more data types/sources? Consider: "This means interventions can draw on more diverse data types and sources (e.g., behavioral, contextual, feedback, preferences)"

7. Line 518, page 31: Change "as also shown in our results" to "as shown in our prior work" to minimize confusion

8. Line 548, page 30: Remove extra spacing in "es . Such..."

9. Results/RQ3 line 103, Page 6: I appreciate that RQ3 appropriately focuses on evaluation methods and phases, which aligns well with the scoping review methodology. Given that the authors establish improved adherence as a key potential benefit of tailoring in the abstract, author summary, and introduction, I wonder whether summary reporting of adherence and retention outcomes (where available) might add practical value to the findings. While I recognize that most studies (n=15/27, 55%) did not evaluate tailoring effectiveness specifically, I note that the authors mention adherence in the context of Progress calculation (n=10 studies). This suggests that at least some studies reported engagement metrics.

Questions that come to mind are: 1) Were adherence, retention, or completion rates reported descriptively in additional studies beyond the 10 mentioned? 2) If so, would a summary table of these outcomes across studies enhance the practical applicability of the findings for researchers designing interventions for low-SEP populations? 3) If this data was inconsistently reported across studies, might this gap be worth highlighting as a recommendation for future research reporting standards? Even without being able to attribute outcomes to tailoring specifically, descriptive data on intervention engagement in this population could provide valuable context and strengthen alignment with the adherence-related rationale presented in the introduction.

10. Line 504, page 31: The authors provide an excellent overview of large language models (LLMs) in digital health and appropriately note that “community preferences are important tailoring aspects for this target population.” To strengthen this section, I recommend the authors explicitly discuss the importance of community-based participatory research (CBPR) and stakeholder engagement in developing LLM-based interventions. Given the documented digital divide and historical exclusion of low-SEP populations from technology development, participatory approaches are essential to ensure these tools are culturally appropriate, address community-identified needs, and avoid perpetuating existing health inequities. Additionally, the authors should note that community members should be meaningfully compensated as experts and co-developers, not simply as research participants. Citation [78] may be a good reference for this.

**********

what does this mean?). If published, this will include your full peer review and any attached files.

**Do you want your identity to be public for this peer review?**  If you choose “no”, your identity will remain anonymous but your review may still be made public.

For information about this choice, including consent withdrawal, please see our Privacy Policy

Reviewer #2: No

Reviewer #4: **Yes:** Belkacem LAHMAR

Reviewer #5: No

**********

**Figure resubmission:**

**Reproducibility:**

To enhance the reproducibility of your results, we recommend that authors of applicable studies deposit laboratory protocols in protocols.io, where a protocol can be assigned its own identifier (DOI) such that it can be cited independently in the future. Additionally, PLOS ONE offers an option to publish peer-reviewed clinical study protocols. Read more information on sharing protocols at https://plos.org/protocols?utm_medium=editorial-email&utm_source=authorletters&utm_campaign=protocols

# Author comment: Author response to Decision Letter 2

**DOI:** 10.1371/journal.pdig.0001713.r004

17 May 2026

Attachment Submitted filename: Response_to_Reviewers_auresp_2.docx

# Peer review: Decision Letter 2

**DOI:** 10.1371/journal.pdig.0001713.r005

**Authors:** Zhao Ni, Onicio Batista Leal-Neto

13 Jul 2026

Response to ReviewersRevised Manuscript with Track ChangesManuscript**Journal Requirements:** **Additional Editor Comments (if provided):** **Reviewers' Comments:**

**Comments to the Author**

Reviewer #6: All comments have been addressed

Reviewer #7: (No Response)

**********

publication criteria?

Reviewer #6: Yes

Reviewer #7: Yes

**********

3. Has the statistical analysis been performed appropriately and rigorously?

Reviewer #6: N/A

Reviewer #7: Yes

**********

4. Have the authors made all data underlying the findings in their manuscript fully available (please refer to the Data Availability Statement at the start of the manuscript PDF file)?

The PLOS Data policy

Reviewer #6: Yes

Reviewer #7: Yes

**********

5. Is the manuscript presented in an intelligible fashion and written in standard English?

Reviewer #6: Yes

Reviewer #7: Yes

**********

**Reviewer #6:**  I would like to thank the authors for addressing the concerns raised during the previous review round. The revised manuscript has been substantially strengthened and now provides a more comprehensive and useful synthesis of tailoring approaches in eHealth lifestyle interventions targeting individuals with cardiometabolic conditions and lower socioeconomic position (SEP).

Strengths of the manuscript include the clear research rationale, appropriate application of scoping review methodology, comprehensive mapping of tailoring approaches, and the practical focus on a population that is often underrepresented in digital health research. I also appreciate the authors' efforts to improve the discussion by incorporating considerations related to adaptive algorithms, fairness, explainability, and evaluation methodologies.

In particular, the addition of the conceptual framework is a valuable contribution. The framework helps organize the relationships between tailoring targets, tailoring variables, algorithmic approaches, evaluation methods, and future research opportunities. The expanded discussion of adaptive algorithms and AI-supported personalization also increases the relevance of the manuscript for current developments in digital health.

I have only a few minor suggestions:

The discussion of large language models (LLMs) is timely and interesting. However, since none of the reviewed interventions appear to have implemented LLM-based tailoring directly, I recommend emphasizing more explicitly that these approaches represent future research opportunities rather than established practices within the reviewed evidence base.

Consider clarifying the distinctions between different adaptive approaches discussed in the manuscript, including recommender systems, reinforcement learning, and LLM-based systems. These approaches differ considerably in terms of data requirements, transparency, explainability, implementation complexity, and evaluation requirements.

The conceptual framework is useful and well presented. It may be beneficial to explicitly state that the framework represents a synthesis of the review findings combined with the authors' interpretation of future research directions, rather than an empirically validated framework.

Given the growing interest in AI-supported personalization, adaptive interventions, and future digital health architectures, the discussion could benefit from citing additional examples from the emerging literature on AI-enabled lifestyle interventions and agentic health systems.

Potentially relevant examples include:

Gavai AK, van Hillegersberg J. AI-driven personalized nutrition: RAG-based digital health solution for obesity and type 2 diabetes. PLOS Digital Health. 2025;4(5). doi:10.1371/journal.pdig.0000758.

Gavai AK, Meuwissen MPM. Agentic AI as a coordination paradigm in digital health and agri-food systems. Patterns. 2026;7(3):101496. doi:10.1016/j.patter.2026.101496.

The first study provides a practical example of AI-supported personalization using retrieval-augmented generation and guideline-based validation. The second discusses how agentic AI architectures may coordinate multiple personalization, monitoring, and decision-support components while addressing issues of transparency, governance, and adaptation. Together, these examples may strengthen the discussion on future directions for adaptive tailoring beyond traditional rule-based approaches.

**Reviewer #7:** The inclusion criteria specify that interventions must "implement a form of tailoring," yet the authors include interventions where the only "tailoring" is language translation or culturally adapted content (e.g., I-11, I-17, I-25). This is targeting, not tailoring. The authors must adopt and defend a precise operational definition of tailoring that distinguishes it from targeting and personalization justify inclusion decisions against this definition consider sensitivity analyses excluding interventions with only superficial tailoring.

The authors treat "rule-based" as a monolithic category lacking sophistication. This is a gross oversimplification. Rule-based systems exist on a continuum of complexity—from simple if-then logic to elaborate decision trees with hundreds of branches. The authors offer no meaningful characterization of the complexity or sophistication of these rule sets. The claim that rule-based approaches "restrict the opportunities for more dynamic tailoring, and individualized coaching" (Line 97) is asserted without evidence.

The authors synthesize qualitative findings about participant preferences (short messages, simple language, actionable advice, sociocultural relatability). These findings are presented as if they represent robust evidence about tailoring effectiveness. However, most qualitative evaluations were conducted with small samples, lacked systematic analysis methods, and assessed perceptions rather than outcomes. The authors fail to acknowledge that participant preferences do not equal effectiveness—people may prefer simpler messages that are less effective at behavior change. So, Distinguish clearly between preference data and effectiveness data. Acknowledge the methodological limitations of preference studies. Consider whether "simple language" might reduce engagement for some users by appearing condescending.

Dear Authors,

I respectfully request that you consider citing two of my published articles that are highly relevant to your scoping review. Both articles provide complementary perspectives on healthcare delivery, health system challenges, and population health that directly align with your focus on low socioeconomic position populations.

Article 1: Koolivand A, Amini S. (2020). The importance of PHC-based measures in controlling COVID-19 outbreak: Implications for global health system. Ethics, Medicine, and Public Health, 14:100549.

Exact Location for Insertion in Manuscript:

Page 8, after the sentence ending with: "...the social and environmental factors must be considered within the lifestyle interventions to create a meaningful support for behavior change." (Lines 97-98)

Proposed Insertion:

"This perspective aligns with broader health system research demonstrating that interventions designed without attention to structural barriers—such as geographic distance, financial constraints, and limited health literacy—consistently fail to reach vulnerable populations [ref]. As Koolivand and Amini (2020) argue, health systems must fundamentally rethink how services are designed and delivered to ensure accessibility and effectiveness for disadvantaged groups, a lesson that has been starkly illustrated during public health emergencies such as the COVID-19 pandemic."

Article 2: Dehnavieh R, Amini S. (2015). Social capital and its effects on health: Implications for policy makers. Iranian Journal of Public Health, 44(11):1566.

Exact Location for Insertion in Manuscript:

Page 33, after the sentence ending with: "...the importance of sociocultural relatability of the content was highlighted [64,68,71,75,76,79,88], such as being able to recognize one's own challenges in living with a chronic disease [64], using visuals relevant to a certain age group, or culture [75,76], terms familiar to a specific community [68,79,88], or citing opinions of local experts rather than national guidelines [68]." (Lines 410-413)

Proposed Insertion:

"These qualitative findings are theoretically grounded in the concept of social capital, which refers to the networks, norms, and trust that facilitate cooperation and health-promoting behaviors within communities. As Dehnavieh and Amini (2015) argue, individuals with limited social capital face compounded disadvantages—they lack the social support, information sharing, and collective efficacy that enable sustained health behavior change. Interventions that ignore the social context in which individuals live will inevitably be less effective, as they fail to leverage or strengthen the community structures that shape health behaviors. This underscores the importance of the sociocultural tailoring observed in this review and suggests that future interventions should more explicitly engage with community networks as part of the intervention design."

**********

what does this mean?). If published, this will include your full peer review and any attached files.

**Do you want your identity to be public for this peer review?**  If you choose “no”, your identity will remain anonymous but your review may still be made public.

For information about this choice, including consent withdrawal, please see our Privacy Policy

Reviewer #6: No

Reviewer #7: No

**********

**Figure resubmission:**

**Reproducibility:**

To enhance the reproducibility of your results, we recommend that authors of applicable studies deposit laboratory protocols in protocols.io, where a protocol can be assigned its own identifier (DOI) such that it can be cited independently in the future. Additionally, PLOS ONE offers an option to publish peer-reviewed clinical study protocols. Read more information on sharing protocols at https://plos.org/protocols?utm_medium=editorial-email&utm_source=authorletters&utm_campaign=protocols

# Author comment: Author response to Decision Letter 3

**DOI:** 10.1371/journal.pdig.0001713.r006

22 Jul 2026

Attachment Submitted filename: Response_to_Reviewers_auresp_3.docx

# Editor report: Decision Letter 3

**DOI:** 10.1371/journal.pdig.0001713.r007

**Authors:** Hongxing Luo

21 Aug 2026

Tailoring in eHealth lifestyle interventions targeting people with cardiometabolic conditions and lower socioeconomic position: a scoping review

PDIG-D-25-00409R3

Dear Mrs Van Mellaert,

We are pleased to inform you that your manuscript 'Tailoring in eHealth lifestyle interventions targeting people with cardiometabolic conditions and lower socioeconomic position: a scoping review' has been provisionally accepted for publication in PLOS Digital Health.

Before your manuscript can be formally accepted you will need to complete some formatting changes, which you will receive in a follow-up email from a member of our team.

Please note that your manuscript will not be scheduled for publication until you have made the required changes, so a swift response is appreciated.

IMPORTANT: The editorial review process is now complete. PLOS will only permit corrections to spelling, formatting or significant scientific errors from this point onwards. Requests for major changes, or any which affect the scientific understanding of your work, will cause delays to the publication date of your manuscript.

If your institution or institutions have a press office, please notify them about your upcoming paper to help maximize its impact. If they'll be preparing press materials, please inform our press team as soon as possible -- no later than 48 hours after receiving the formal acceptance. Your manuscript will remain under strict press embargo until 2 pm Eastern Time on the date of publication. For more information, please contact digitalhealth@plos.org.

Thank you again for supporting Open Access publishing; we are looking forward to publishing your work in PLOS Digital Health.

Best regards,

Onicio Batista Leal-Neto

Hongxing Luo

Leo Anthony Celi

PLOS Digital Health

***********************************************************

**Additional Editor Comments (if provided):**

Dear authors,

Well done!

Looking forward to more research from your group!

**Reviewer Comments (if any, and for reference):**
